# Prevalence of Depression and Anxiety Among Adults With Chronic Pain: A Systematic Review and Meta-Analysis

**DOI:** 10.1001/jamanetworkopen.2025.0268

**Published:** 2025-03-07

**Authors:** Rachel V. Aaron, Scott G. Ravyts, Nicolette D. Carnahan, Kavya Bhattiprolu, Nicole Harte, Claire C. McCaulley, Lauren Vitalicia, Alexandria B. Rogers, Stephen T. Wegener, Joanne Dudeney

**Affiliations:** 1 Department of Physical Medicine and Rehabilitation, Johns Hopkins University School of Medicine, Baltimore, Maryland; 2 Department of Psychology, University of North Carolina at Charlotte; 3 Department of Psychology, University of Delaware, Newark; 4 School of Psychological Science, Macquarie University, New South Wales, Australia; 5 Johns Hopkins University School of Arts and Sciences, Baltimore, Maryland; 6 New York University Langone Health, New York, New York

## Abstract

**Importance:**

Depression and anxiety are common among adults with chronic pain, but their prevalence is unclear.

**Objectives:**

To evaluate the prevalence of depression and anxiety among adults with chronic pain and identify factors that moderate prevalence.

**Data Sources:**

A literature search was conducted of MEDLINE, Embase, PsycINFO, and Cochrane Library from January 2013 to October 2023.

**Study Selection:**

Studies reporting the prevalence of depression or anxiety using a validated assessment tool among adults with chronic pain (excluding chronic headache disorders).

**Data Extraction and Synthesis:**

A total of 31 159 initial records were identified, and 5177 full texts were screened. Data were extracted per the Preferred Reporting Items for Systematic Reviews and Meta-analyses guideline using Covidence. Two independent reviewers completed abstract screening, full-text review, and data extraction and rated risk of bias. Random-effects meta-analyses were applied to pool prevalence, assess moderation, and compare prevalence of depression or anxiety among samples with chronic pain vs control samples.

**Main Outcomes and Measures:**

Prevalence of depression and anxiety based on clinically significant symptoms or diagnosis. The moderators of prevalence identified were pain condition, recruitment setting, continent, age, percentage female, and pain duration.

**Results:**

The search identified 376 studies from 50 countries comprising 347 468 individuals (mean [SD] age, 51.3 [9.5] years; 70.0% female) with chronic pain. Among adults with chronic pain, clinical symptoms of depression were present in 39.3% (95% CI, 37.3%-41.1%; *I*^2^ = 98.9%), and clinical symptoms of anxiety were present in 40.2% (95% CI, 38.0%-42.4%; *I*^2^ = 99.0%). Prevalence differed by pain condition (highest among samples of people with fibromyalgia [depression, 54.0% (95% CI, 48.5%-59.4%); anxiety, 55.5% (95% CI, 50.4%-60.4%)]; lowest among samples of people with arthritis conditions [eg, osteoarthritis: depression, 29.1% (95% CI, 20.3%-39.7%); anxiety, 17.5% (95% CI, 6.6%-38.8%)]) and was highest among younger people (depression, β = −0.02 [95% CI, −0.03 to −0.01]; anxiety, β = −0.02 [95% CI, –0.03 to –0.01]) and women (depression, β = 0.69 [95% CI, 0.31-1.08]; anxiety, β = 0.90 [95% CI, 0.48-1.33]). With regard to diagnoses, 36.7% (95% CI, 29.0%-45.1%) had a major depressive disorder, and 16.7% (95% CI, 11.8%-23.2%) had generalized anxiety disorder. Women, younger people, and people with nociplastic pain (ie, pain arising from altered nociception without tissue damage) were most likely to have depression and anxiety.

**Conclusions and Relevance:**

In this systematic review and meta-analysis of depression and anxiety among individuals with chronic pain, approximately 40% of adults had clinically significant depression and anxiety. Women, younger people, and people with nociplastic pain were most likely to have depression and anxiety. The co-occurrence of chronic pain with depression and anxiety is a significant public health concern necessitating routine screening in clinical settings, equitable access to specialty care, and innovative treatment development.

## Key Points

**Question:**

How prevalent are depression and anxiety among adults with chronic pain?

**Findings:**

This systematic review and meta-analysis identified 376 studies comprising 347 468 individuals with chronic pain from 50 countries, with a pooled prevalence of 39.3% for depression and 40.2% for anxiety; the prevalences were highest among samples of people with fibromyalgia and samples of people who were younger and predominantly female. The prevalences of depression and anxiety were significantly higher among individuals with chronic pain than in both clinical and nonclinical control groups.

**Meaning:**

This study suggests that the prevalences of depression and anxiety among adults with chronic pain are approximately 40%; to address this significant public health concern, it is essential to routinely screen for mental health symptoms in clinical settings where people with chronic pain are treated.

## Teaser

This systematic review and meta-analysis evaluates the prevalence of depression and anxiety among adults with chronic pain and examines factors that moderate depression and anxiety prevalence in this population.

## Introduction

Chronic pain, defined as pain that persists for more than 3 months,^1^ is a prevalent and disabling condition affecting 21% of adults.^2^ Chronic pain impairs myriad life domains and is frequently associated with psychological distress, including symptoms of depression and anxiety.^3^ After accounting for numerous biopsychosocial factors, depression and anxiety are among the best predictors of chronic pain.^4^ Depression and anxiety are leading causes of disability worldwide, contributing to poor quality of life and reduced life expectancy.^5^ Population studies indicate that 20% to 40% of adults with chronic pain have co-occurring depression and anxiety^6,7,8^; however, published prevalence rates of depression and anxiety vary widely, even within specific chronic pain conditions. Not only are few treatments available for these comorbidities, but people with mental health comorbidities are frequently rejected from chronic pain clinics and excluded from clinical trials.^9,10^ A comprehensive systematic review and meta-analysis of the prevalence of depression and anxiety is essential to clarify the extent of depression and anxiety among adults with chronic pain worldwide; addressing this gap will help guide public health initiatives and inform treatment development to promote positive health outcomes for people with chronic pain. As such, the aims of the present review were to (1) evaluate the overall pooled prevalence of depression and anxiety, including clinical symptoms and *Diagnostic and Statistical Manual of Mental Disorders* (Fifth Edition) (*DSM-5*) diagnoses, among adults with chronic pain; (2) characterize the moderating effect of pain condition, geographic location, recruitment setting (clinical vs community), age, gender, and pain duration on prevalence; and (3) compare the prevalence of depression and anxiety among adults with vs without chronic pain.

## Methods

This preregistered review (PROSPERO CRD42022370083) was conducted according to the Preferred Reporting Items for Systematic Reviews and Meta-analyses (PRISMA) reporting guideline.^11^ Data for chronic headache disorder will be reported separately, consistent with Cochrane reporting practices^12^ and the broader chronic pain field.^13^ The unique diagnostic and classification system for chronic headache disorders (The International Classification of Headache Disorders), and features within, requires a distinct approach to assessing eligibility and study quality for meta-analysis and requires extracting and analyzing unique information (eg, headache frequency) and reporting a fine-grained analysis by headache type.

### Search Strategy

We searched MEDLINE, Embase, PsycINFO, and Cochrane Library from January 2013 to October 2023. The year 2013 was selected to correspond with the publication of the *DSM-5*,^14^ which introduced changes to depressive and anxiety disorder diagnoses. We identified studies reporting the prevalence of depression and anxiety (symptoms exceeding a clinical cutoff or *DSM-5* diagnoses) among adults with any chronic pain condition. Covidence was used to store records and for study selection, data extraction, and quality assessment. See eAppendix 1 in Supplement 1 for search criteria and the Box for eligibility criteria.

Box. **Eligibility Criteria**

**Inclusion criteria**

Chronic pain (except chronic headache or migraine) clearly defined (ie, clinical diagnosis of chronic pain or a chronic pain condition; individual seen in chronic pain clinic)
Adult sample
(a) Assessment of depression and/or anxiety symptoms using a validated assessment (the cutoff score applied to evaluate clinical symptoms must be specified) or (b) assessment of any *DSM-5* depression and/or anxiety diagnosis using a standardized measure delivered systematically to the full sample
Reports data that allow the computation of the prevalence of clinical symptoms (ie, percentage who exceed a cutoff score) or diagnosis
Peer-reviewed, full-text publication

**Exclusion criteria**

1. Studies where data on anxiety or depression cannot be discerned from one another or other symptoms (eg, psychological distress, internalizing disorders)
2. Studies that require particular symptoms levels of depression or anxiety (high or low)
3. Studies that include or exclude based on current or historical psychiatric illness (unless it is specified that the disorders of exclusion are not depression or anxiety disorders) or treatment (eg, psychotherapy, SSRIs)
4. Studies where data on chronic pain cannot be discerned from other pain conditions (eg, acute pain)
5. Non-English studies
6. Nonempirical studies

Abbreviations: *DSM-5*, *Diagnostic and Statistical Manual of Mental Disorders* (Fifth Edition); SSRIs, selective serotonin reuptake inhibitors.

### Study Selection

Each abstract, and then full text, was screened by 2 independent reviewers to assess eligibility. Conflicts were identified and resolved between screeners or in consultation with the first author.

### Data Extraction

Two reviewers independently extracted data (sample size, study location, recruitment setting, chronic pain characteristics, sample characteristics, details of assessment, and prevalence rates) from included articles. Conflicts were resolved between extractors or in consultation with a senior reviewer.

### Assessment of Quality

Two reviewers independently rated the quality of included studies. Conflicts were resolved between extractors or in consultation with a senior reviewer (R.V.A., S.G.R., N.D.C., and J.D.). The Joanna Briggs Institute (JBI) Checklist^15^ for cross-sectional studies was used, adapted for condition (chronic pain) and symptoms (depression and anxiety), and designed to isolate items most relevant to all study designs. In brief, studies were evaluated based on eligibility criteria, the reporting of participants and setting characteristics, chronic pain assessment, and depression and anxiety assessment. An overall score was calculated, from which quality was deemed high, medium, or low. The full rating tool and scoring criteria are provided in eAppendix 2 in Supplement 2.

### Statistical Analysis

Pooled prevalence was determined by analyzing study-specific event rates implementing DerSimonian and Laird random-effects models^16,17^ using Comprehensive Meta-Analysis, version 4 (Biostat).^18^ Heterogeneity was assessed using *I^2^* (range, 0%-100%, with higher scores indicating more heterogeneity),^19^ Cochrane *Q*, and 95% prediction intervals. Publication bias was assessed with visual inspection of funnel plots and the Egger test of asymmetry^20^; when significant (*P* < .05), the Duval and Tweedie trim-and-fill method was used to adjust for missing studies.^21^ When control group data were available, the magnitude of difference was characterized by Hedges *g* (0.2 = small, 0.5 = medium, 0.8 = large).^22^

We characterized the moderating effects of pain condition, recruitment setting, continent, age, percentage female, and chronic pain duration using subgroup (categorical) or meta-regression (continuous) random-effects models, when more than 3 studies (K > 3) were available.^23^ When comparing prevalence between chronic pain and control samples, a subgroup analysis based on nonclinical (healthy or community controls) vs clinical (nonchronic pain diagnosis or treatment-seeking samples) control samples was performed. The results of moderation analyses are reported regardless of statistical significance. Because various depression and anxiety assessments were used, we conducted a post hoc sensitivity analysis to evaluate differences in prevalence by assessment type. All *P* values were from 2-sided tests, and results were deemed statistically significant at *P* < .05.

## Results

### Study Characteristics

A total of 31 159 initial records were identified, and we screened 30 777 titles and abstracts (Figure). Data from 376 studies (including 415 distinct samples of people with chronic pain) were included, representing 347 468 adults with chronic pain and 160 564 control participants. The mean (SD) age among people with chronic pain was 51.3 (9.5) years, and the participants were primarily female (70.0%). The most common chronic pain conditions were mixed (K = 142), fibromyalgia (K = 85), chronic low back pain (K = 37), and rheumatoid arthritis (K = 20). Studies spanned 50 countries; the most common country was the US (K = 74). Among included samples of people with chronic pain, 24.1% reported data on race or ethnicity, 57.1% on educational level, 40.0% on employment status, and 17.4% on income. Due to variability in reporting practices, these data were unable to be synthesized. Data from control samples were available for 94 studies (65 nonclinical, 28 clinical, 1 mixed). Individual study characteristics are reported in eTable 1 in Supplement 1, details about the assessment of depression and anxiety in eTable 2 in Supplement 1, and eReferences in Supplement 1.

**Figure.**
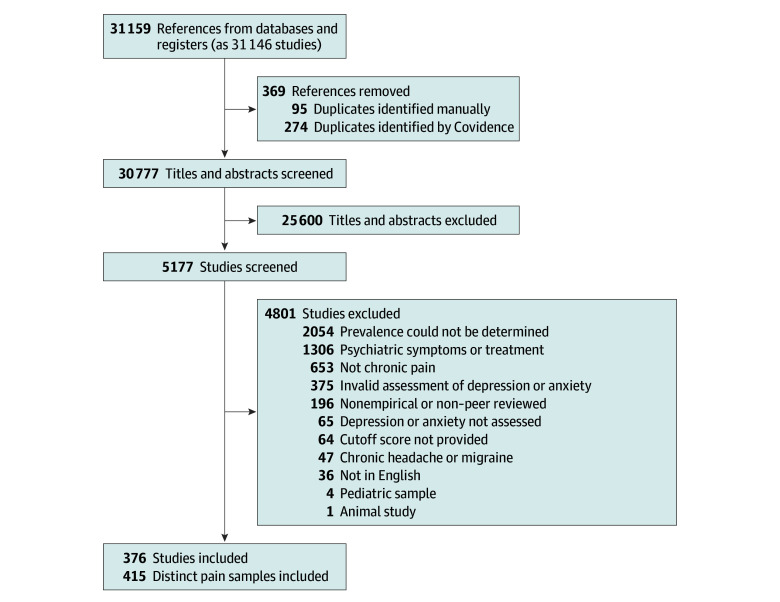
Flow Diagram

### Overall Prevalence Rates

Pooled prevalence is reported in Table 1, forest plots in eFigures 1 and 2 in Supplement 1, moderation analyses in Table 2 and Table 3, and individual study-level data in eTables 3 to 6 in Supplement 1. Diagnostic data refer to current or 12-month prevalence (most commonly reported).

**Table 1.** Overall Pooled Prevalence of Depression and Anxiety

Characteristic	K value	No.	Prevalence, % (95% CI)	Prediction interval, %	*Q* value	*I*^2^, %
Clinical depression symptoms						
Depression	355	230 684	39.3 (37.3-41.1)	0.12-0.76	27 776.21	98.9
Clinical anxiety symptoms						
Anxiety	185	256 556	40.2 (38.0-42.4)	0.17-0.69	17 621.50	99.0
Depressive disorders						
MDD	41	17 291	36.7 (29.0-45.1)	0.05-0.86	2734.26	98.7
PDD	10	4826	6.3 (3.0-12.5)	0.004-0.50	183.37	95.1
Anxiety disorders						
GAD	24	20 441	16.7 (11.8-23.2)	0.03-0.61	1035.49	97.8
PD	14	5700	7.5 (4.5-12.3)	0.01-0.43	240.10	97.8
SAD	8	4486	2.2 (1.0-5.8)	0.001-0.40	83.53	91.6

Abbreviations: GAD, generalized anxiety disorder; MDD, major depression disorder; PD, panic disorder; PDD, persistent depressive disorder; SAD, social anxiety disorder.

**Table 2.** Results of Random-Effects Subgroup Analysis

Moderator^a^	K value	Prevalence, % (95% CI)	Prediction interval, %	*Q* value	*P* value	*I*^2^, %
**Clinical symptoms of depression**						
Pain condition						
Fibromyalgia	70	54.0 (48.5-59.4)	16.13-87.73	2269.10	<.001	96.7
Complex regional pain syndrome	4	47.4 (22.1-74.1)	0.36-99.55	51.88	<.001	94.2
Not specified or mixed conditions	121	40.2 (36.9-43.6)	13.10-75.01	19 082.65	<.001	99.4
Temporal mandibular disorder	6	39.2 (19.6-63.1)	1.72-95.97	241.89	<.001	97.9
Chronic neuropathic pain	9	37.5 (28.7-47.1)	13.08-70.46	61.40	<.001	87.0
Chronic pelvic pain	15	36.3 (27.7-45.8)	9.84-74.84	181.41	<.001	92.3
Chronic low back pain	32	33.3 (27.4-39.9)	8.90-71.87	764.35	<.001	95.9
Chronic back pain	5	33.3 (21.9-47.2)	5.13-82.18	59.71	<.001	93.3
Chronic musculoskeletal pain	8	29.2 (18.5-42.8)	4.39-78.76	322.44	<.001	97.8
Osteoarthritis	12	29.1 (20.3-39.7)	6.13-71.99	207.73	<.001	94.7
Spondyloarthritis	9	29.0 (20.3-39.5)	7.34-67.77	63.83	<.001	87.5
Rheumatoid arthritis	18	27.2 (21.5-33.8)	8.67-59.58	218.66	<.001	92.2
Postsurgical pain	4	22.5 (10.2-42.5)	0.37-95.70	27.50	<.001	89.1
Other arthritis (mixed, other, not otherwise specified)	4	22.2 (10.8-40.3)	0.46-94.66	89.04	<.001	96.6
Total between-group variance	NA	NA	NA	60.61	<.001	NA
Recruitment setting						
Clinical	255	41.3 (39.1-43.6)	14.34-74.77	16 836.25	<.001	98.5
Mixed	18	34.9 (27.6-43.0)	10.62-70.76	351.70	<.001	95.2
Community	47	30.6 (25.6-36.0)	7.44-70.69	4942.12	<.001	99.1
Total between-group variance	NA	NA	NA	13.61	<.001	NA
Continent						
South America	23	45.3 (36.7-54.1)	12.55-82.63	364.71	<.001	94.0
Asia	65	40.9 (35.6-46.5)	10.32-80.68	2376.58	<.001	97.3
Europe	123	39.8 (36.9-42.8)	15.02-71.21	4154.25	<.001	97.1
North America	75	38.4 (34.0-42.9)	10.91-75.97	6223.47	<.001	98.8
Australia	15	36.5 (29.1-44.6)	12.97-68.98	1066.31	<.001	98.7
Africa	6	36.1 (13.9-66.4)	0.57-98.25	154.50	<.001	96.8
Mixed	7	28.1 (20.5-37.3)	8.04-63.68	365.77	<.001	98.4
Total between-group variance	NA	NA	NA	8.55	.20	NA
**Clinical symptoms of anxiety**						
Pain condition						
Fibromyalgia	37	55.5 (50.4-60.4)	27.32-80.51	549.70	<.001	93.5
Chronic pelvic pain	11	51.8 (38.9-64.5)	12.19-89.27	254.72	<.001	96.1
Not specified or mixed conditions	59	39.1 (35.4-42.9)	16.29-67.89	14 981.99	<.001	99.6
Chronic neuropathic pain	9	33.8 (28.5-39.6)	19.21-52.31	23.25	<.001	65.6
Temporal mandibular disorder	6	32.4 (17.3-52.3)	2.20-91.06	173.66	<.001	97.1
Chronic low back pain	15	32.1 (26.1-38.7)	12.60-60.76	130.26	<.001	89.3
Rheumatoid arthritis	10	26.3 (18.2-36.6)	6.00-66.70	75.88	<.001	88.1
Postsurgical pain	4	26.0 (10.1-52.4)	0.17-98.67	40.35	<.001	92.6
Osteoarthritis	4	17.5 (6.6-38.8)	0.11-97.51	44.23	<.001	93.2
Total between-group variance	NA	NA	NA	61.71	<.001	NA
Recruitment location						
Mixed	7	42.5 (24.5-62.8)	3.59-93.62	195.23	<.001	96.9
Clinical	146	41.0 (38.8-43.3)	20.02-65.92	10 891.99	<.001	98.7
Community	13	34.4 (25.2-44.9)	7.97-76.07	1740.88	<.001	99.3
Total between-group variance	NA	NA	NA	1.54	.46	NA
Continent						
South America	12	50.7 (44.6-56.8)	29.00-72.12	86.73	<.001	87.3
Asia	19	42.0 (35.8-48.5)	18.16-70.28	719.04	<.001	97.5
Europe	67	39.0 (36.2-42.0)	20.26-61.72	4329.40	<.001	98.5
North America	38	36.2 (31.5-41.3)	13.54-67.34	1176.75	<.001	96.9
Australia	12	36.2 (31.5-24.1)	29.00-72.12	132.22	<.001	91.7
Mixed	6	35.7 (29.0-43.0)	10.26-64.48	28.32	<.001	82.3
Total between-group variance	NA	NA	NA	20.26	<.001	NA
**Major depressive disorder**						
Pain condition						
Fibromyalgia	10	38.3 (27.9-49.9)	10.06-77.47	82.42	<.001	89.1
Not specified or mixed conditions	18	34.5 (23.4-47.7)	0.03-99.96	1803.79	<.001	99.1
Total between-group variance	NA	NA	NA	0.19	.66	NA
Recruitment location						
Mixed	4	45.2 (22.6-70.0)	0.60-99.11	46.03	<.001	93.5
Clinical	32	41.1 (35.4-46.9)	15.24-72.96	624.42	<.001	95.0
Community	5	13.7 (7.7-23.3)	1.26-66.48	332.24	<.001	98.8
Total between-group variance	NA	NA	NA	18.13	<.001	NA
Continent						
South America	5	46.5 (29.5-64.4)	4.94-93.55	77.41	<.001	94.8
North America	8	44.2 (29.9-59.4)	7.54-88.48	264.56	<.001	97.4
Asia	10	36.4 (18.2-59.5)	1.49-95.59	537.11	<.001	98.3
Europe	14	28.4 (18.0-41.6)	3.29-82.15	588.67	<.001	97.8
Total between-group variance	NA	NA	NA	3.69	.30	NA
**Generalized anxiety disorder**						
Pain condition						
Fibromyalgia	8	33.3 (15.4-57.9)	1.22-95.30	197.97	<.001	96.5
Not specified or mixed conditions	6	11.2 (5.2-22.4)	0.58-73.08	244.61	<.001	98.0
Total between-group variance	NA	NA	NA	4.27	.04	NA
Recruitment location						
Clinical	18	22.2 (15.6-30.4)	4.01-65.94	302.94	<.001	94.4
Community	4	3.9 (2.3-6.7)	0.28-37.54	180.54	<.001	98.3
Total between-group variance	NA	NA	NA	29.20	<.001	NA
Continent						
Europe	7	21.8 (9.0-44.0)	0.67-92.03	222.03	<.001	97.3
Asia	8	14.1 (4.3-37.9)	0.13-95.40	297.11	<001	97.6
North America	5	12.6 (7.1-21.3)	1.21-62.92	193.91	<.001	97.9
Total between-group variance	NA	NA	NA	1.14	.57	NA

Abbreviation: NA, not applicable.

^a^ All moderators in order of most to least prevalent. Subgroup analysis was only performed when more than 3 studies (K > 3) ere available at the individual moderator level.

**Table 3.** Results of Random-Effects Meta-Regression

Characteristic	β (95% CI)	*z* Score	*P* value
**Clinical symptoms of depression**			
Age (K = 303)			
Intercept	0.52 (0.01 to 1.03)	2.00	.05
Mean age	−0.02 (−0.03 to −0.01)	−3.72	<.001
Female (K = 346)			
Intercept	−0.91 (−1.19 to −0.63)	−6.38	<.001
% Female	0.69 (0.31 to 1.08)	3.53	<.001
Duration (K = 101)			
Intercept	−0.46 (−0.84 to −0.08)	−2.39	.02
Mean duration	0.00 (−0.002 to 0.004)	0.60	.55
**Clinical symptoms of anxiety**			
Age (K = 158)			
Intercept	0.44 (−0.06 to 0.95)	1.72	.09
Mean age	−0.02 (−0.03 to −0.01)	−3.33	<.001
Female (K = 181)			
Intercept	−1.10 (−1.41 to −0.79)	−6.93	<.001
% Female	0.90 (0.48 to 1.33)	4.19	<.001
Duration (K = 58)			
Intercept	−1.08 (−1.44 to −0.71)	−5.78	<.001
Mean duration	0.01 (0.002 to 0.004)	4.03	<.001
**Major depressive disorder**			
Age (K = 35)			
Intercept	0.21 (−1.89 to 2.31)	0.20	.85
Mean age	−0.02 (−0.06 to 0.03)	−0.66	.51
Female (K = 37)			
Intercept	−0.71 (−1.74 to 0.32)	−1.35	.18
% Female	0.20 (−1.15 to 1.55)	0.29	.77
Duration (K = 15)			
Intercept	−0.96 (−1.90 to −0.04)	−2.03	.04
Mean duration	0.01 (−0.003 to 0.02)	1.46	.14
**Persistent depressive disorder**			
Age (K = 8)			
Intercept	−1.81 (−9.09 to 5.48)	−0.49	.63
Mean age	−0.01 (−0.16 to 0.13)	−0.18	.85
Female (K = 10)			
Intercept	−4.92 (−7.60 to −2.24)	−3.60	<.001
% Female	2.93 (−0.44 to 6.30)	1.71	.09
**Generalized anxiety disorder**			
Age (K = 21)			
Intercept	−1.59 (−5.29 to 2.11)	−0.84	.40
Mean age	0.00 (−0.07 to 0.08)	0.09	.93
Female (K = 21)			
Intercept	−2.92 (−4.27 to −1.57)	−4.24	<.001
% Female	2.70 (1.24 to 4.16)	2.28	<.001
Duration (K = 8)			
Intercept	−1.35 (−2.49 to −0.21)	−2.33	.02
Mean duration	0.00 (−0.01 to 0.01)	−0.31	.76
**Panic disorder**			
Age (K = 12)			
Intercept	−3.94 (−5.83 to −2.05)	−4.08	<.001
Mean age	0.04 (−0.001 to 0.08)	1.89	.06
Female (K = 14)			
Intercept	−2.59 (−4.55 to −0.63)	−2.59	.001
% Female	0.00 (−0.03 to 0.03)	0.08	.94
Duration (K = 4)			
Intercept	−3.08 (−5.64 to −0.52)	−2.36	.02
Mean duration	0.02 (−0.01 to 0.04)	1.11	.27

#### Prevalence of Clinical Symptoms of Depression

The pooled prevalence of clinical symptoms of depression (K = 355; n = 230 684) was 39.3% (95% CI, 37.3%-41.1%), with considerable heterogeneity (*I*^2^ = 98.9%) (Table 1). The Egger test suggested evidence of possible publication bias (β = −1.27; *P* = .03); however, trim-and-fill analysis identified no missing studies. There was a significant association of pain condition (*Q* = 60.61; *P* < .001) and recruitment location (*Q* = 13.61; *P* < .001); prevalence was highest among samples of people with fibromyalgia (54.0% [95% CI, 48.5%-59.4%]) and those recruited from clinical settings (41.3% [95% CI, 39.1%-43.6%]) (Table 2). Prevalence was lowest among samples of people with osteoarthritis (29.1% [95% CI, 20.3%-39.7%]). There was an association of age and percentage of women; prevalence was higher among samples of younger individuals (β = −0.02 [95% CI, −0.03 to −0.01]; *P* < .001) and samples more predominantly comprising women (β = 0.69 [95% CI, 0.31-1.08]; *P* < .001) (Table 3). There was no association of continent or pain duration.

The prevalence of depression among control samples (K = 67; n = 111 998) was 13.9% (95% CI, 11.5%-16.7%); the prevalence was higher (moderate association) in groups with chronic pain vs control groups (*g* = 0.63 [95% CI, 0.54-0.71]; *I*^2^ = 86.3%). Prevalence was lower among nonclinical (11.1% [95% CI, 8.6%-14.3%]) vs clinical (19.0% [95% CI, 14.2%-25.0%]) controls; however, the overall association of control group type was not statistically significant (*Q* = 3.8; *P* = .05). Significant group differences with chronic pain remained for both nonclinical (*g* = 0.71 [95% CI, 0.60-0.82]; *I*^2^ = 86.4%) and clinical (*g* = 0.51 [95% CI, 0.33-0.68]; *I*^2^ = 86.0%) controls.

#### Prevalence of Clinical Symptoms of Anxiety

The pooled prevalence of clinical symptoms of anxiety (K = 185; n = 256 556) was 40.2% (95% CI, 38.0%-42.4%) with considerable heterogeneity (*I*^2^ = 99.0%) (Table 1). The Egger test suggested evidence of possible publication bias (β = −3.28; *P* < .001); however, trim-and-fill analysis identified no missing studies. There was a significant association of pain type (*Q* = 61.71; *P* < .001) and continent (*Q* = 20.26; *P* < .001); prevalence was highest among samples of people with fibromyalgia (55.5% [95% CI, 50.4%-60.4%]) and those from South America (50.7% [95% CI, 44.6%-56.8%]) (Table 2). Prevalence was lower among samples of people with osteoarthritis (17.5% [95% CI, 6.6%-38.8%]). Prevalence was higher among samples of people who were younger (β = −0.02 [95% CI, –0.03 to –0.01]; *P* < .001), more predominantly female (β = 0.90 [95% CI, 0.48-1.33]; *P* < .001), and had a longer pain duration (β = 0.01 [95% CI, 0.002-0.004]; *P* < .001) (Table 3). There was no significant association of recruitment location.

The prevalence of anxiety among control samples (K = 39; n = 9216) was 16.4% (95% CI, 11.6%-22.6%); the prevalence was higher (large association) in groups with chronic pain vs control groups (*g* = 0.82 [95% CI, 0.66-0.99]; *I*^2^ = 87.2%). Prevalence was lower among nonclinical (12.3% [95% CI, 6.8%-21.2%]) vs clinical (22.5% [95% CI, 13.8%-34.5%]) controls; however, the overall association of control group type was not statistically significant (*Q* = 3.7; *P* = .05). Significant differences with chronic pain remained for both nonclinical (*g* = 1.01 [95% CI, 0.75-1.27]; *I*^2^ = 87.0%) and clinical (*g* = 0.64 [95% CI, 0.37-0.91]; *I*^2^ = 89.0%) controls.

#### Prevalence of Diagnosed Depressive Disorders

##### Major Depressive Disorder

The pooled prevalence of major depressive disorder (MDD) (K = 41; n = 17 291) was 36.7% (95% CI, 29.0%-45.1%), with considerable heterogeneity (*I*^2^ = 98.7%) (Table 1). The Egger test suggested no evidence of publication bias (β = 2.48; *P* = .26). There was an association of recruitment location (*Q* = 18.13; *P* < .001); prevalence was highest among samples of individuals recruited from mixed (45.2% [95% CI, 22.6%-70.0%]) or clinical (41.1% [95% CI, 35.4%-46.9%]) settings (Table 2). There was no association of pain condition, continent, age, percentage female, or pain duration. Among 11 control samples, the prevalence of MDD was 10.1% (95% CI, 6.4%-15.6%), a moderately sized difference compared with the chronic pain sample (*g* = 0.50 [95% CI, 0.31-0.68]; *I*^2^ = 82.7%).

##### Persistent Depressive Disorder

The pooled prevalence of persistent depressive disorder (K = 10; n = 4826) was 6.3% (95% CI, 3.0%-12.5%), with high heterogeneity (*I*^2^ = 95.1%) (Table 1). The Egger test suggested no evidence of publication bias (β = 2.47; *P* = .59). There was no association of age or percentage female.

#### Prevalence of Diagnosed Anxiety Disorders

##### General Anxiety Disorder

The pooled prevalence of generalized anxiety disorder (GAD) (K = 24; n = 20 441) was 16.7% (95% CI, 11.8%-23.2%), with high heterogeneity (*I*^2^ = 97.8%) (Table 1). The Egger test suggested evidence of publication bias (β = 5.68; *P* = .001); however, no studies were identified in trim-and-fill analysis. There was a significant association of pain type (*Q* = 4.27; *P* = .04) and recruitment location (*Q* = 29.20; *P* < .001): GAD was highest among samples of people with fibromyalgia (33.3% [95% CI, 15.4%-57.9%]) and those recruited from clinical settings (22.2% [95% CI, 15.6%-30.4%]) (Table 2). Prevalence was higher among samples of people who were more predominantly female (β = 2.70 [95% CI, 0.27-3.63; *P* < .001) (Table 3). There was no association of continent, age, or pain duration. Among 4 control samples, the prevalence of GAD was 3.5% (95% CI, 3.0%-4.0%), a moderately sized difference compared with chronic pain (*g* = 0.56 [95% CI, 0.46-0.66]; *I*^2^ = 0.0%).

##### Panic Disorder

The pooled prevalence of panic disorder (K = 14; n = 5700) was 7.5% (95% CI, 4.5%-12.3%), with high heterogeneity (*I*^2^ = 97.8%) (Table 1). The Egger test suggested no evidence of publication bias (β = −2.68; *P* = .29). There was no association of age, percentage female, or pain duration. Comparison data were available for 4 control samples; among the 4 control samples, the prevalence of panic disorder was 4.3% (95% CI, 2.2%-8.3%), which was not statistically different than chronic pain (*g* = 0.45 [95% CI, −0.01 to 0.91]; *I*^2^ = 0.0%).

##### Social Anxiety Disorder

The pooled prevalence of social anxiety disorder (K = 8; n = 4486) was 2.2% (95% CI, 1.0%-5.8%), with high heterogeneity (*I*^2^ = 91.6%). The Egger test suggested no evidence of publication bias (β = 0.30; *P* = .91). There was no association of age or percentage female.

#### Sensitivity Analysis

There was an association of assessment type with the prevalence of depression symptoms, anxiety symptoms, GAD, and panic disorder (eTable 7 in Supplement 1).

### Study Quality

Overall, 57% of studies had low quality ratings, 21% had medium quality ratings, and 22% had high quality ratings (eFigure 3 and eTable 8 in Supplement 1). The largest source of bias was the description of study participants and settings, with 75% of studies deemed low quality.

## Discussion

Approximately 40% of adults with chronic pain had clinically significant depression and anxiety, based on data from 415 samples representing 347 468 adults with chronic pain from 50 countries. Overall, the prevalences of depression and anxiety statistically differed between chronic pain conditions and were highest among younger adults and women, although heterogeneity remained high across most subgroup analyses.

The prevalence of clinical symptoms of depression and anxiety was highest among individuals with pain conditions associated with nociplastic mechanisms (ie, pain due to altered nociception in the absence of tissue damage^24,25^), including fibromyalgia, complex regional pain syndrome, and temporal mandibular disorder. For example, 54.0% of people with fibromyalgia had clinical symptoms of depression, and 55.5% had clinical symptoms of anxiety. In contrast, people with pain conditions with greater nociceptive or neuropathic involvement, including various types of arthritis, had the lowest prevalence of depression (22.1%-29.1%) and anxiety (17.5%-26.3%). Although underlying pain mechanisms are not mutually exclusive, the overall pattern of findings aligns with evidence that psychological distress and adverse life experiences increase the risk for chronic nociplastic pain.^24,26,27,28^ Clinical symptoms of anxiety, but not depression, were greater among samples of people with longer pain duration. These findings cannot speak to the directionality of associations of chronic pain with depression and anxiety, but directionality may vary based on pain type; for example, depression and anxiety may be more likely to be associated with the development of nociceptive pain. Most chronic pain treatments do not address co-occurring depression and anxiety; the present findings underscore the need for innovative treatment development to address these prevalent comorbidities, particularly for nociplastic pain.

Among adults with chronic pain, 36.7% met diagnostic criteria for MDD and 16.7% for GAD, markedly higher than population norms (eg, 12-month prevalence of approximately 10% for MDD^29,30^ and 2% for GAD^30,31^), highlighting the prevalence of diagnosable conditions requiring targeted treatment. The prevalences of MDD and depressive symptoms were similar, suggesting good specificity of depression symptom inventories in chronic pain. The prevalences of clinical anxiety symptoms (40.2%) and GAD (16.7%) diverged, likely reflecting a disparity in symptom duration required by *DSM-5* criteria (6 months) and symptom inventories (1-2 weeks). It is also possible that general inventories are more sensitive to pain-focused anxiety presentations (eg, pain catastrophizing and kinesiophobia) that do not meet GAD criteria. Assessing both general and pain-specific anxiety in clinical settings is ideal, as they require distinct treatment approaches.

Adults with chronic pain were more likely than both the clinical and nonclinical control groups to have clinical symptoms of depression and anxiety; elevated depression and anxiety may be unique to chronic pain, rather than solely associated with having a medical condition. Understanding the mechanisms underlying chronic pain and depression and anxiety is vital to inform their treatment.

### Implications

Changes in chronic pain care are needed at the individual clinician and systems levels to address the co-occurrence of chronic pain and depression and anxiety. People with chronic pain and co-occurring mental health comorbidities are frequently rejected from specialty pain care^9^; mental health comorbidities are common among adults with chronic pain, and ensuring equitable access to specialty care is essential. For physicians treating individuals with chronic pain in primary care and specialty practice, systematic screening of depression and anxiety is critical,^32^ as is having a network of mental health referral sources when a positive screening result is detected. Short-term, cost-effective, and remotely delivered psychological treatments for chronic pain are becoming increasingly available and can be recommended to individuals with chronic pain.^33,34^

Although interdisciplinary pain treatment, including the integration of mental health care professionals, remains the criterion standard for treating chronic pain,^35^ most patients lack access to interdisciplinary care.^36,37^ At the systems level, broader implementation of interdisciplinary care is needed. Although the efficacy of psychological treatments for pain-related outcomes is clear, they do not result in large improvements in psychological distress,^12^ A large-scale population study found that 37% of US adults with chronic pain experienced depression and anxiety despite receiving mental health treatment.^38^ Few psychological treatments are available to treat chronic pain and co-occurring depression and anxiety,^39,40,41,42^ although treatments that adopt an embodied approach and incorporate affective targets of intervention may be more effective for people with heightened mental health symptoms.^43,44^ The present findings underscore the need for developing and scaling targeted treatments that address co-occurring chronic pain and depression and anxiety.

### Limitations

This study has some limitations. We followed rigorous PRISMA reporting standards; however, reporting standards have not been specifically tailored for systematic reviews of prevalence,^45^ and meta-analyses of prevalence can be misleading if the variability of included samples is high.^16,46^ To procure a richer pool of prevalence rates, we did not limit inclusion based on study type (eg, observational or clinical trial), but this may have introduced bias,^47^ and our inclusion of all study types required adapting a rigorous, but single, quality checklist.^15^ We took several steps to limit bias and address heterogeneity, including using random-effects (vs fixed-effects) meta-analysis, applying stringent eligibility criteria based on population (chronic pain) and condition (depression and anxiety), and systematically assessing study quality.^16^ For example, we excluded studies that excluded participants based on psychological diagnosis or treatment and studies that did not assess depression and anxiety using a validated assessment delivered uniformly to all participants (eg, unstandardized interviews and *International Classification of Diseases, Ninth Revision* codes). We also systematically assessed heterogeneity across 6 factors known to be associated with depression and anxiety in chronic pain. Nevertheless, heterogeneity in this study remains largely unexplained, with high heterogeneity across most subgroup analyses. It is possible that examining heterogeneity at intersecting moderator levels (eg, women from North America with fibromyalgia) would yield further insights into heterogeneity; however, this was beyond the scope of the present study.

Only a minority of studies (24.1%) reported race or ethnicity, and studies lacked gender diversity (k = 2 reported nonbinary gender identities), precluding investigating heterogeneity by race and ethnicity or gender (beyond percentage of female). Some studies did not adhere to recommended clinical cutoff scores, and the quality of approximately one-third of studies was rated poor for their assessment of depression and anxiety; a significant association of measure type with the prevalence of depression and anxiety suggests that some measures overestimated or underestimated true prevalence.

## Conclusions

In this meta-analysis study of depression and anxiety among indivduals with chronic pain, we found prevalence rates of approximately 40%. Adults who were younger, female, and with nociplastic pain were more likely to have co-occurring chronic pain and depression or anxiety. Systematically screening for depression and anxiety in clinical settings in which chronic pain is treated is critical. Offering equitable access to care and representation in clinical trials, as well as innovative treatment targeting chronic pain and co-occurring depression and anxiety, is essential to promote positive outcomes for adults with chronic pain.

## Notes

Supplement 1. **eAppendix 1.** Search Criteria **eAppendix 2.** Rating of Study Quality **eTable 1.** Study Characteristics **eTable 2.** Overview of Symptom Measures and Diagnostic Assessments Used **eFigure 1.** Prevalence of Clinical Symptoms of Depression, Forest Plots **eFigure 2.** Prevalence of Clinical Symptoms of Anxiety, Forest Plots **eTable 3.** Pooled Prevalence of Clinical Symptoms of Depression **eTable 4.** Pooled Prevalence of Clinical Symptoms of Anxiety **eTable 5.** Pooled Prevalence of Depressive Disorders **eTable 6.** Pooled Prevalence of Anxiety Disorders **eTable 7.** Sensitivity Analysis by Measure Type **eFigure 3.** Risk of Bias Overview **eTable 8.** Individual Study Risk of Bias Score
eReferences.

Supplement 2.
Data Sharing Statement
