# Supplementary material for: Prevalence of Depression and Anxiety Among Adults With Chronic Pain: A Systematic Review and Meta-Analysis
Source: JAMA Netw Open. 2025 Mar 7;8(3):e250268. doi: 10.1001/jamanetworkopen.2025.0268 (PMC11889470; doi:10.1001/jamanetworkopen.2025.0268)
Supplement: Supplement 1. — eAppendix 1. Search Criteria eAppendix 2. Rating of Study Quality eTable 1. Study Characteristics eTable 2. Overview of Symptom Measures and Diagnostic Assessments Used eFigure 1. Prevalence of Clinical Symptoms of Depression, Forest Plots eFigure 2. Prevalence of Clinical Symptoms of Anxiety, Forest Plots eTable 3. Pooled Prevalence of Clinical Symptoms of Depression eTable 4. Pooled Prevalence of Clinical Symptoms of Anxiety eTable 5. Pooled Prevalence of Depressive Disorders eTable 6. Pooled Prevalence of Anxiety Disorders eTable 7. Sensitivity Analysis by Measure Type eFigure 3. Risk of Bias Overview eTable 8. Individual Study Risk of Bias Score eReferences. [file jamanetwopen-e250268-s001.pdf]

Supplementary Online Content

Aaron RV, Ravyts SG, Carnahan ND, et al. Prevalence of depression and anxiety among adults with chronic pain: a systematic review and meta-analysis. *JAMA Netw Open.* 2025;8(3):e250268. doi:10.1001/jamanetworkopen.2025.0268

eAppendix 1. Search Criteria

eAppendix 2. Rating of Study Quality

eTable 1. Study Characteristics

eTable 2. Overview of Symptom Measures and Diagnostic Assessments Used

eFigure 1. Prevalence of Clinical Symptoms of Depression, Forest Plots

eFigure 2. Prevalence of Clinical Symptoms of Anxiety, Forest Plots

eTable 3. Pooled Prevalence of Clinical Symptoms of Depression

eTable 4. Pooled Prevalence of Clinical Symptoms of Anxiety

eTable 5. Pooled Prevalence of Depressive Disorders

eTable 6. Pooled Prevalence of Anxiety Disorders

eTable 7. Sensitivity Analysis by Measure Type

eFigure 3. Risk of Bias Overview

eTable 8. Individual Study Risk of Bias Score

eReferences.

This supplementary material has been provided by the authors to give readers additional information about their work.

© 2025 Aaron RV et al. *JAMA Network Open.*

eAppendix 1. Search Criteria

Ovid Medline

exp Chronic Pain/ or ((chronic* or recurrent* or persistent* or longterm or "long term") adj3 (pain* or headache or headaches or "head ache" or "head aches" or hemicrania or cephalodyni* or cephalalgi* or cephalgi* or discomfort or ache* or neuralgi* or dysmenorrhea)).tw. or (exp Chronic Pain/ or ((chronic* or recurrent* or persistent* or longterm or "long term").tw. and (exp Headache/ or headache disorders/ or headache disorders, primary/ or exp tension-type headache/ or trigeminal autonomic cephalalgias/ or exp cluster headache/ or exp paroxysmal hemicrania/ or headache disorders, secondary/ or exp post-dural puncture headache/ or exp post-traumatic headache/ or exp vascular headaches/ or exp neuralgia/ or exp dysmenorrhea/))) or (exp Fibromyalgia/ or migraine disorders/ or exp migraine with aura/ or exp migraine without aura/ or exp ophthalmoplegic migraine/ or (Hemicrania or migraine* or fibromyalgia* or "fibro-myalgia" or "fibro-myalgias" or cranialgi* or cephalea or migrainous).tw.)

exp Depression/ or exp Depressive Disorder/ or exp Anxiety/ or exp Anxiety Disorders/ or phobi*.tw. or depress*.tw. or depressed.tw. or depression.tw. or depressive.tw. or depressions.tw. or anxiet*.tw. or anxious*.tw. or agoraphobias.tw. or phobia.tw. or phobias.tw. or phobic.tw. or panic.tw. or dysthymic.tw. or dysthymia.tw. or dysthymias.tw. or "seasonal affective disorder".tw. or "seasonal affective disorders".tw. or "seasonal mood disorder".tw. or "seasonal mood disorders".tw. or distress.tw. or dysphori*.tw.

1 and 2

limit 3 to (english language and yr="2013 -Current")

Embase

'chronic pain'/exp OR (((chronic* OR recurrent* OR persistent* OR longterm OR 'long term') NEAR/3 (pain* OR headache OR headaches OR 'head ache' OR 'head aches' OR hemicrania OR cephalodyni* OR cephalalgi* OR cephalgi* OR discomfort OR ache* OR neuralgi* OR dysmenorrhea)):ti,ab,kw) OR ((chronic*:ti,ab,kw OR recurrent*:ti,ab,kw OR persistent*:ti,ab,kw OR longterm:ti,ab,kw OR 'long term':ti,ab,kw) AND ('chronic pain'/exp OR 'headache'/exp OR 'headache and facial pain'/de OR 'chronic daily headache'/exp OR 'cough headache'/exp OR 'exertional headache'/exp OR 'hypnic headache'/exp OR 'ictal headache'/exp OR 'postdural puncture headache'/exp OR 'postural headache'/exp OR 'primary headache'/exp OR 'secondary headache'/exp OR 'sexual headache'/exp OR 'sinus headache'/exp OR 'stabbing headache'/exp OR 'tension headache'/exp OR 'thunderclap headache'/exp OR 'trigeminal autonomic cephalalgia'/exp OR 'cluster headache'/exp OR 'paroxysmal hemicrania'/exp OR 'vascular headache'/exp OR 'neuralgia'/exp OR 'dysmenorrhea'/exp)) OR hemicrania:ti,ab,kw OR migraine*:ti,ab,kw OR fibromyalgia*:ti,ab,kw OR 'fibro-myalgia':ti,ab,kw OR 'fibro-myalgias':ti,ab,kw OR cranialgi*:ti,ab,kw OR cephalea:ti,ab,kw OR migrainous:ti,ab,kw OR 'fibromyalgia'/exp OR 'migraine'/de OR 'basilar type migraine'/exp OR 'complicated migraine'/exp OR 'episodic migraine'/exp OR 'hemiplegic migraine'/exp OR 'menstrual migraine'/exp OR 'migraine aura'/exp OR 'migraine with aura'/exp OR 'migraine without aura'/exp OR 'migrainous infarction'/exp OR 'migralepsy'/exp OR 'ophthalmoplegic migraine'/exp OR 'retinal migraine'/exp OR 'status migrainosus'/exp OR 'transformed migraine'/exp OR 'vestibular migraine'/exp

AND

'depression'/exp OR 'anxiety'/exp OR 'anxiety disorder'/exp OR phobi*:ti,ab,kw OR depress*:ti,ab,kw OR depressed:ti,ab,kw OR depression:ti,ab,kw OR depressive:ti,ab,kw OR depressions:ti,ab,kw OR anxiet*:ti,ab,kw OR anxious*:ti,ab,kw OR agoraphobia:ti,ab,kw OR agoraphobias:ti,ab,kw OR phobia:ti,ab,kw OR phobias:ti,ab,kw OR phobic:ti,ab,kw OR panic:ti,ab,kw OR dysthymic:ti,ab,kw OR dysthymia:ti,ab,kw OR dysthymias:ti,ab,kw OR "seasonal affective disorder":ti,ab,kw OR "seasonal affective disorders":ti,ab,kw OR

© 2025 Aaron RV et al. *JAMA Network Open.*

"seasonal mood disorder":ti,ab,kw OR "seasonal mood disorders":ti,ab,kw OR distress:ti,ab,kw OR dysphori*:ti,ab,kw

((#1 AND #2 AND [2013-2023]/py) NOT 'conference abstract'/it) AND [english]/lim

Cochrane

([mh "Chronic Pain"] OR ((chronic* OR recurrent* OR persistent* OR longterm OR "long term") NEAR/3 (pain* OR headache OR headaches OR "head ache" OR "head aches" OR hemicrania OR cephalodyni* OR cephalalgi* OR cephalgi* OR discomfort OR ache* OR neuralgi* OR dysmenorrhea)):ti,ab,kw) OR ([mh "Chronic Pain"] OR ((chronic* OR recurrent* OR persistent* OR longterm OR "long term"):ti,ab,kw AND ([mh Headache] OR [mh "Headache Disorders"] OR [mh Neuralgia] OR [mh Dysmenorrhea]))) OR [mh "Fibromyalgia"] OR [mh "Migraine Disorders"] OR (migraine* OR fibromyalgia* OR "fibro-myalgia" OR "fibro-myalgias" OR cranialgi* OR cephalea OR migrainous):ti,ab,kw

AND

([mh "Depression"] OR [mh "Depressive Disorder"] OR [mh "Anxiety"] OR [mh "Anxiety Disorders"] OR [mh "Manifest Anxiety Scale"] OR [mh "Test Anxiety Scale"] OR phobi*:ti,ab,kw OR depress*:ti,ab,kw OR depressed:ti,ab,kw OR depression:ti,ab,kw OR depressive:ti,ab,kw OR depressions:ti,ab,kw OR anxiet*:ti,ab,kw OR anxious*:ti,ab,kw OR agoraphobia:ti,ab,kw OR agoraphobias:ti,ab,kw OR phobia:ti,ab,kw OR phobias:ti,ab,kw OR phobic:ti,ab,kw OR panic:ti,ab,kw OR dysthymic:ti,ab,kw OR dysthymia:ti,ab,kw OR dysthymias:ti,ab,kw OR "seasonal affective disorder":ti,ab,kw OR "seasonal affective disorders":ti,ab,kw OR "seasonal mood disorder":ti,ab,kw OR "seasonal mood disorders":ti,ab,kw OR distress:ti,ab,kw OR dysphori*:ti,ab,kw)

#1 AND #2

Limits: Since 2013

PsycINFO

(DE "Chronic Pain" OR TI(((chronic* OR recurrent* OR persistent* OR longterm OR "long term") N3 (pain* OR headache OR headaches OR "head ache" OR "head aches" OR hemicrania OR cephalodyni* OR cephalalgi* OR cephalgi* OR discomfort OR ache* OR neuralgi* OR dysmenorrhea))) OR AB(((chronic* OR recurrent* OR persistent* OR longterm OR "long term") N3 (pain* OR headache OR headaches OR "head ache" OR "head aches" OR hemicrania OR cephalodyni* OR cephalalgi* OR cephalgi* OR discomfort OR ache* OR neuralgi* OR dysmenorrhea)))) OR (DE "Chronic Pain" OR ((TI (chronic* OR recurrent* OR persistent* OR longterm OR "long term") OR AB (chronic* OR recurrent* OR persistent* OR longterm OR "long term")) AND (DE "Headache" OR DE "Neuralgia" OR DE "Dysmenorrhea")) OR DE "Migraine Headache" OR DE "Fibromyalgia" OR TI (migraine* OR fibromyalgia* OR "fibro-myalgia" OR "fibro-myalgias" OR cranialgi* OR cephalea OR "migrainous") OR AB (migraine* OR fibromyalgia* OR "fibro-myalgia" OR "fibro-myalgias" OR cranialgi* OR cephalea OR "migrainous")

AND

(DE "Cyclothymic Disorder" OR DE "Major Depression" OR DE "Dysthymic Disorder" OR DE "Depression (Emotion)" OR DE "Koro" OR DE "Phobias" OR DE "Acrophobia" OR DE "Agoraphobia" OR DE "Claustrophobia" OR DE "Ophidiophobia" OR DE "Social Phobia" OR DE "Anxiety" OR DE "Health Anxiety" OR DE "Social Anxiety" OR DE "Anxiety Disorders" OR DE "Generalized Anxiety Disorder" OR DE "Panic Attack" OR DE "Panic Disorder" OR DE "Separation Anxiety Disorder" OR DE "Trichotillomania" OR TI (phobi* OR depress* OR depressed OR depression OR depressive OR depressions OR anxiet* OR anxious* OR agoraphobia OR agoraphobias OR phobia OR phobias OR phobic OR panic OR dysthymic OR dysthymia OR dysthymias OR "seasonal affective disorder" OR "seasonal affective disorders" OR "seasonal mood disorder"

© 2025 Aaron RV et al. *JAMA Network Open.*

OR "seasonal mood disorders" OR distress OR dysphori*) OR AB (phobi* OR depress* OR depressed OR depression OR depressive OR depressions OR anxiet* OR anxious* OR agoraphobia OR agoraphobias OR phobia OR phobias OR phobic OR panic OR dysthymic OR dysthymia OR dysthymias OR "seasonal affective disorder" OR "seasonal affective disorders" OR "seasonal mood disorder" OR "seasonal mood disorders" OR distress OR dysphori*))

AND

DT 01012013-12312023

Limits: English

PubMed

("Chronic Pain"[Mesh] OR ((chronic*[tw] OR recurrent*[tw] OR persistent*[tw] OR longterm[tw] OR "long term"[tw]) AND ("Headache"[Mesh] OR "Headache Disorders"[Mesh] OR pain*[tw] OR headache[tw] OR headaches[tw] OR "head ache"[tw] OR "head aches"[tw] OR hemicrania[tw] OR cephalodyni*[tw] OR cephalalgi*[tw] OR cephalgi*[tw] OR discomfort OR ache* OR "Neuralgia"[Mesh] OR neuralgi* OR "Dysmenorrhea"[Mesh] OR dysmenorrhea))) OR "Fibromyalgia"[Mesh] OR "Migraine Disorders"[Mesh] OR migraine*[tw] OR fibromyalgia*[tw] OR "fibro-myalgia"[tw] OR "fibro-myalgias"[tw] OR cranialgi*[tw] OR cephalea[tw] OR "migrainous"[tw]

AND

("Depression"[Mesh] OR "Depressive Disorder"[Mesh] OR "Anxiety"[Mesh] OR "Anxiety Disorders"[Mesh] OR phobi*[tw] OR depress*[tw] OR depressed[tw] OR depression[tw] OR depressive[tw] OR depressions[tw] OR anxiet*[tw] OR anxious*[tw] OR agoraphobia[tw] OR agoraphobias[tw] OR "neurotic disorder"[tw] OR phobia[tw] OR phobias[tw] OR phobic[tw] OR panic[tw] OR dysthymic[tw] OR dysthymia[tw] OR dysthymias[tw] OR "seasonal affective disorder"[tw] OR "seasonal affective disorders"[tw] OR "seasonal mood disorder"[tw] OR "seasonal mood disorders"[tw] OR distress[tw] OR dysphori*[tw])

(#1 AND #2 AND ("2013/01/01"[Date - Publication] : "2023/12/31"[Date - Publication])) NOT (Medline[sb])

Limits: English

© 2025 Aaron RV et al. *JAMA Network Open.*

eAppendix 2. Rating of Study Quality

The rating of study quality for the current study was adapted from the Joanna Briggs Institute Checklist for Analytical Cross-Sectional Studies. The full study-quality scoring manual developed for this study is available at request.

1. Were the criteria for inclusion in the sample clearly defined?
2. Were the study subjects and the setting described in detail?
3. Were objective, standard criteria used for the measurement of chronic pain?
4. Was clinical depressive symptomology measured in a valid and reliable way?
5. Was clinical anxiety symptomology measured in a valid and reliable way?
6. Were depression diagnoses assessed in a valid and reliable way?
7. Were anxiety diagnoses assessed in a valid and reliable way?

Response options: 1 (yes), 0 (no or unclear)

Scoring: The overall study quality rating was determined by summing the total score (0-7), dividing by the number of available ratings, and converting to a percentage. This allowed standardization of scores where different studies had different numbers of items rated (i.e., not all studies had ratings for items 4-7). Higher scores indicated lower rating bias. Studies were deemed to have high (0-49%), medium (50-74%), or low (75-100%) reporting bias.

© 2025 Aaron RV et al. *JAMA Network Open.*

eTable 1. Study Characteristics

Study ID	Country	N (Pain)	Pain Demographics A=Age; F=% Female; R=Race, Ethnicity or Nationality	Pain Condition	N (Control)	Control Demographics A=Age; F=% Female; R=Race, Ethnicity or Nationality
Aagaard 2023	Denmark	33	A: 48.9; F: 75.8%; R: NR	Mixed		
Abrams 2013	USA	252	A: 48.6; F: 74.1%; R: NR	Mixed		
Ahmad 2023	Pakistan	96	A: 51.6; F: 59.4%; R: NR	Fibromyalgia		
Ajo 2017	Spain	125	A: 57; F: 0%; R: NR	Mixed		
Åkerblom 2020	Sweden	159	A: 41.5; F: 84.9%; R: 75% Swedish	Mixed		
Akui 2022	Japan	20	A: 63; F: 30%; R: NR	Post Cancer Pain		
Alamam 2019	Saudi Arabia	115	A: 40; F: 63.0%; R: 100% Saudi Arabian	Chronic LBP		
Alberts 2020	USA	65	A: 44.1; F: 53.8%; R: NR	Post Cancer Pain		
Alciati 2018	Italy	87	A: 46.95; F: 100%; R: 100% Caucasian	Fibromyalgia		
Alciati 2020	Italy	118	A: 45.7; F: 90.7%; R: 100% Caucasian	Fibromyalgia		
Alhalal 2021	Saudi Arabia	233	A: 51.32; F: 67.0%; R: NR	Mixed		
Allaire 2018	Canada	268	A: 34.3; F: 100%; R: NR	Chronic Pelvic Pain		
Al-Maharbi 2018	Saudi Arabia	200	A: NR; F: 64.0%; R: NR	Mixed		
Aloush 2021	Israel	231	A: NR; F: 90.9%; R: NR	Fibromyalgia		
AlShukaili 2022	Oman	287	A: 47.89; F: 67.2%; R: NR	Mixed		
AlvesRodrigues 2022	Brazil	46	A: 54.63; F: 56.5%; R: 13% White; 71.74% Multiracial; 15.21% Black	Persistent Spinal Pain Syndrome		
Amatsu 2022	Japan	74	A: 71.03; F: 0%; R: NR	Mixed	933	A: 68.6; F: 0%; R: NR
Amital 2014	NR	23	A: 53.7; F: 100%; R: NR	Fibromyalgia		
Ammitzbøll 2021	Denmark	199	A: 62.5; F: 91.3%; R: 100% Caucasian	RA	215	A: 49; F: 71.4%; R: 96.1% Caucasian
Andersen 2016	Denmark	125	A: 44.12; F: 78.4%; R: NR	Mixed		
Annagür 2014	Turkey	108	A: 47.8; F: 70.4%; R: NR	Mixed	54	A: 45.3; F: 79.6%; R: NR
Antaky 2017	Canada	251	A: 61.5; F: 66.5%; R: NR	Mixed		
Antunes 2013	NR	193	A: 43.8; F: 72.5%; R: NR	Chronic LBP		
Ardigo 2016	NR	53	A: 80.64; F: 73.6%; R: NR	Mixed		
Areias 2023	USA	559	A: 47.3; F: 51.6%; R: NR	Chronic LBP		
Argoff 2016	Multi-Continent	2062	A: 49.56; F: 92.8%; R: NR	Fibromyalgia		
Asada 2022	Japan	376	A: 72.7; F: 60.2%; R: NR	Chronic LBP	270.5	A: 71.6; F: 52.7%; R: NR
Asada 2022_B	Japan	189	A: 72.4; F: 54.9%; R: NR	Chronic Musculoskeletal Pain	270.5	A: 71.6; F: 52.7%; R: NR
Aşçıbaşı 2022	NR	62	A: 42.44; F: 90.3%; R: NR	Fibromyalgia		
Asih 2014	NR	324	A: 45.86; F: 32.5%; R: 59% Caucasian; 26% African American; 12% Hispanic/Latino; 1% Other	Chronic Musculoskeletal Pain		

© 2025 Aaron RV et al. *JAMA Network Open.*

Asih 2016	NR	542	A: 47.2; F: 43.5%; R: 51% White; 26% African American; 21% Hispanic; 3% Other	Occupational Musculoskeletal Disorder		
Asseyer 2021	Germany	22	A: 42.4; F: 72.7%; R: NR	Myelin oligodendrocyte glycoprotein antibody-associated disease	21	A: 35.9; F: 61.9%; R: NR
Ataoglu 2013	Turkey	109	A: 36.2; F: 25.7%; R: NR	Spinal Cord Injury	31	A: 36.2; F: 25.7%; R: NR
Avluk 2014	Turkey	44	A: 33.9; F: 25.0%; R: NR	Spinal Cord Injury		
Bagnato 2014	Italy	96	A: 50.39; F: 69.8%; R: NR	Mixed		
Bailey 2020	USA	10,264	A: 43.57; F: 50%; R: NR	Mixed		
Bapir 2023	UK	1254	A: 45.75; F: 45.9%; R: NR	Mixed		
Barbosa 2016	Brazil	52	A: 33; F: 100%; R: NR	Chronic Pelvic Pain		
Barjandi 2021	Sweden	161	A: 47.5; F: 100%; R: 76.4% Scandinavian	TMD		
Barjandi 2021_B	Sweden	81	A: 47.5; F: 100%; R: 76.4% Scandinavian	Fibromyalgia		
Barth 2014	USA	307	A: 51; F: 39.0%; R: NR	Chronic Pancreatic Pain		
Barton 2021	USA	204	A: 57.2; F: 58.3%; R: 68% White; 3% Black; 22% Latinx/Hispanic; 7% Other	RA		
Bayrak 2020	Turkey	310	A: 37.9; F: 49.4%; R: NR	Irritable Bowel Syndrome	304	A: 37.7; F: 49.7%; R: NR
Bayram 2014	NR	30	A: 39.1; F: 100%; R: NR	Fibromyalgia	15	A: 40.8; F: 100%; R: NR
Bayram 2014_B	NR	30	A: 39.1; F: 100%; R: NR	RA	15	A: 40.8; F: 100%; R: NR
Bell 2016	USA	6679	A: 53.8; F: 52.1%; R: 76% Non-Hispanic white; 11% Non-Hispanic Black; 7% Hispanic; 6% other	Mixed	68,321	
Bilen 2022	NR	110	A: 51.9; F: 47.3%; R: NR	Mixed		
Birch 2022	England	16	A: 50.23; F: 75.0%; R: NR	Mixed		
Blakey 2018	USA	667	A: 37.82; F: 19.0%; R: NR	Mixed		
Bohn 2013	Germany	117	A: 49; F: 83.8%; R: 100% Caucasian	Fibromyalgia		
Botros 2022	Canada	369	A: 42.36; F: 78.1%; R: NR	TMD		
Boukhira 2021	Morocco	321	A: 57.04; F: 33.3%; R: 100% Moroccan	Mixed	120	A: 52.4; F: 10.2%; R: 100% Moroccan
Bravo 2019	NR	39	A: 52.31; F: 97.4%; R: NR	Fibromyalgia		
BromleyMilton 2013	Sweden	4069	A: 46; F: 69.1%; R: NR	Mixed		
Bruce 2021	USA	698	A: 48.6; F: 90.3%; R: NR	Fibromyalgia		
Brunner 2023	USA	218	A: 57.2; F: 83.6%; R: NR	Mixed		
Bryant 2016	Australia	175	A: 36; F: 100%; R: NR	Chronic Pelvic Pain		
Bucourt 2021	France	48	A: 47.98; F: 100%; R: NR	Fibromyalgia		
Bucourt 2021_B	France	47	A: 50.89; F: 100%; R: NR	RA		
Bucourt 2021_C	France	47	A: 42.3; F: 100%; R: NR	Spondyloarthritis		

© 2025 Aaron RV et al. *JAMA Network Open.*

Calandre 2022	Spain	287	A: 49.17; F: 94.8%; R: NR	Fibromyalgia		
Calderón 2016	Spain	56	A: 58.3; F: 60.7%; R: NR	Mixed		
Calpin 2017	Ireland	100	A: 54; F: 54.0%; R: NR	Mixed		
Campbell 2015	Australia	3585	A: NR; F: 56.6%; R: NR	Mixed	5256	A: NR; F: 46.4%; R: NR
Campbell 2015b	Australia	1514	A: 58; F: 55.6%; R: 80% Australia; 3% Aboriginal/Torres Strait Islander	Mixed		
Campos 2021	Portugal	115	A: 48.02; F: 100%; R: NR	Fibromyalgia		
Cappe 2021	France	73	A: 43.15; F: 100%; R: NR	Fibromyalgia	73	A: 41; F: 100%; R: NR
Carleton 2022	Canada	2025	A: NR; F: 39.8%; R: NR	Mixed	3023	A: NR; F: 60.2%; R: NR
Carrillo-de-la-Peña 2015	Spain	52	A: 46.16; F: 100%; R: NR	Fibromyalgia	56	A: 45.11; F: 100%; R: NR
Carrillo-Izquierdo 2018	Spain	226	A: 43.8; F: 100%; R: NR	Fibromyalgia	222	A: 42.4; F: 100%; R: NR
Carta 2018	Italy	71	A: 51.12; F: 100%; R: N/A	Fibromyalgia	284	A: 51.06; F: 100%; R: NR
CasteloAzevedo 2021	Brazil	652	A: 55.9; F: 66.0%; R: NR	Chronic Knee Pain	2246	A: NR; F: 49.0%; R: NR
Caumo 2022	Brazil	48	A: 49.07; F: 100%; R: NR	Fibromyalgia		
Chaabouni 2022	Tunisia	49	A: 68.2; F: 0%; R: NR	Chronic Obstructive Pulmonary Disease	19	A: 67.3; F: 0%; R: NR
Chahal-Kummen 2019	Norway	58	A: 46; F: 73.7%; R: NR	Chronic Abdominal Pain	138	
Chahal-Kummen 2023	Norway	110	A: 44.4; F: 80.9%; R: NR	Post Surgical Pain	280	A: 45.2; F: 68.9%; R: NR
Challa 2017	USA	103	A: 63.5 ; F: 75.0%; R: NR	Other Arthritis (Mixed, Other, NOS)		
Chana 2021	England	36	A: 55.1; F: 86.1%; R: 47.2% White; 2.8% African Caribbean; 2.8% South American	Burning mouth syndrome		
Chang 2018	USA	130	A: 60.4; F: 54.6%; R: 50% White; 39% Black; 11% Other	Mixed		
Cheatle 2023	USA	609	A: 38.43; F: 60.6%; R: 100% White	Mixed		
Chen 2022	USA	297	A:*; F: 66.3%; R: NR	Chronic Back Pain		
Cheng 2018	China	664	A: 74.79; F: 85.1%; R: 100% Hong Kong Chinese	Mixed		
Cheng 2022	USA	1193	A: 56.3; F: 70.7%; R: NR	Mixed		
Cho 2017	South Korea	100	A: 49.9; F: 84.8%; R: NR	Mixed		
Choi 2018	South Korea	105	A: 24.9; F: 23.8%; R: NR	Spondyloarthritis		
Chytas 2023	Switzerland	140	A: 54.5; F: 60.7%; R: NR	Mixed		
Ciaramella 2015	Italy	416	A: 60.97; F: 60.7%; R: NR	Chronic LBP		
Ciaramella 2015_B	Italy	463	A: 52.66; F: 68.0%; R: NR	Mixed		
Civelek 2022	Turkey	66	A: 70; F: 100%; R: NR	Chronic LBP		
Clark 2018	New Zealand, UK	21	A: 43; F: 76.2%; R: NR	Chronic LBP		

© 2025 Aaron RV et al. *JAMA Network Open.*

Clark 2019	England, Ireland, New Zealand	165	A: 45; F: 76.4%; R: NR	Chronic LBP		
Cody 2019	USA	483	A: 49.8; F: 21.0%; R: 29% White; 41% Black; 2% Asian; 1% Native Hawaiian; 2% American Indian; 8% Multiple Races; 18% Other	Mixed	610	A: 46.8; F: 19.0%; R: 32% White; 39% Black; 4.0% Asian; 0.1% Native Hawaiian; 2.2% American Indian; 6.2% Multiple Races; 17% Other
Conway 2020	UK	238	A: 33; F: 16.4%; R: 85% UK National; 13% Foreign Commonwealth; 1% Gurkha	Chronic LBP		
Corriger 2022	France	245	A: 50.7; F: 75.8%; R: NR	Mixed		
Costa 2023	Brazil	103	A: NR; F: 72.8%; R: NR	Mixed		
Csupak 2018	Canada	5465	A: 48.5; F: 50.7%; R: 77% Caucaisan; 3% Aboriginal; 8% Asian	Other Arthritis (Mixed, Other, NOS)		
Csupak 2018_B	Canada	5205	A: 48.5; F: 50.7%; R: 77% Caucasian; 3% Aboriginal; 8% Asian	Chronic Back Pain		
DaLuz 2018	Brazil	100	A: 37.8; F: 100%; R: 33% White	Chronic Pelvic Pain	100	A: 37.2; F: 100%; R: 27% White; 73% Non-White
Damci 2022	Netherlands	1310	A: 54.71; F: 69.5%; R: NR	Small Fiber Neuropathy		
Davis 2014	USA	38	A: 57; F: 100%; R: 91% Caucasian; 9% Other	Osteoarthritis		
Davis 2014_B	USA	72	A: 57; F: 100%; R: 91% Caucasian; 9% Other	Fibromyalgia		
Dear 2023	Australia	1249	A:*; F: 77.0%; R: 97.7% Other Australian; 2.3% Aboriginal or Terres Strait Islander	Mixed		
deHeer 2017	Netherlands	280	A: 42.61; F: 95.4%; R: NR	Fibromyalgia		
DeLaRosa 2023	USA	7158	A: NR; F: 0%; R: NR	Mixed		
DelPozo-Cruz 2017	Spain	105	A: 55.26; F: 100%; R: NR	Fibromyalgia		
deMoraesVieira 2014	Brazil	215	A:*; F: 65.1%; R: NR	Chronic LBP		
Depintor 2016	Brazil	182	A: 51.4; F: 69.0%; R: NR	Chronic Back Pain		
DeRoa 2018	France	44	A: 45; F: 100%; R: NR	Fibromyalgia		
Doherty 2017	NR	104	A: 49.29; F: 63.4%; R: NR	Mixed		
Domenech 2013	NR	97	A: 32; F: 83.0%; R: NR	Chronic Knee Pain		
DosSantosProença 2023	Brazil	104	A: 37.21; F: 81.7%; R: NR	TMD	25	A: 34.2; F: 80%; R: NR
Dougados 2017	Asia, Europe, South America	192	A: 31.9; F: 60.1%; R: 73% White; 22% Asian; 5% Other	Spondyloarthritis		
Drummond 2013	Australia	23	A: 43.6; F: 91.3%; R: NR	Fibromyalgia		
Drummond 2013_B	Australia	16	A: 42.7; F: 62.5%; R: NR	RA		

© 2025 Aaron RV et al. *JAMA Network Open.*

D'Souza 2020	USA	593	A: 46.9; F: 90.6%; R: 90% Caucasian; 10% Non-Caucasian	Fibromyalgia		
Dubois-Mendes 2021	Brazil	79	A: NR; F: 91.1%; R: 13% White; 4% Indigenous; 43% Black; 41% Mixed	RA		
Dumolard 2023	France	265	A: 47; F: 91.0%; R: NR	Fibromyalgia		
Durán 2021	Chile	948	A: 49.6; F: 64.2%; R: NR	Mixed	3097	A: 41.5; F: 47.7%; R: NR
Ecija 2022	Spain	231	A: 56.91; F: 100%; R: NR	Fibromyalgia		
Edit 2013	Hungary	102	A: 56.7; F: 83.3%; R: NR	Chronic LBP		
Elrashidi 2018	USA	785	A: 61.95; F: 65.9%; R: NR	Mixed		
Emery 2014	Canada	60	A: 46; F: 73.3%; R: NR	Mixed		
Englbrecht 2019	Germany	975	A: 61; F: 75.1%; R: NR	RA		
Eriksen 2021	Denmark	28	A: 51.3; F: 62.3%; R: NR	CRPS		
Falling 2021	USA	67	A: 37.64; F: 55.0%; R: 94% White; 1.5% African American; 4.5% Asian American	Mixed	67	A: 37.73; F: 55.0%; R: 94% White; 1.5% African American; 4.5% Asian American
Farin 2015	Germany	262	A: 52.2; F: 62.1%; R: NR	Chronic LBP		A: 48.5; F: 47.2%; R: NR
Farzad 2021	NR	83	A: 53.9; F: 68.7%; R: NR	CRPS		
Feingold 2017	Israel	890	A:*; F: 43.3%; R: 69% Israeli	Mixed		
Feingold 2018	Israel	540	A:*; F: 49.3%; R: 60% Israeli	Mixed		
Feingold 2020	Israel	209	A: NR; F: 25.4%; R: NR	Mixed		
Feliu-Soler 2017	Spain	160	A: 57.28; F: 97.5%; R: NR	Fibromyalgia		
Fiegl 2019	Germany	496	A: 48.47; F: 51.2%; R: NR	Mixed		
Filippon 2013	Brazil	114	A: 49.5; F: 100%; R: 83% Caucasian; 17% Non-Caucasian	Fibromyalgia		
Finn 2018	NR	269	A: 36.79; F: 79.2%; R: 51% USA; 17% Ireland; 14% Uk; 7% Canada; 6% Australia; 5% Other	Mixed		
Fischer-Jbali 2022	Austria	26	A: 50.36; F: 100%; R: NR	Fibromyalgia	26	A: 45.65; F: 100%; R: NR
Flores-Curiel 2023	Mexico	98	A: NR; F: 81.6%; R: NR	Mixed		
Foti 2022	Italy	171	A: 57; F: 0%; R: NR	RA		
Foti 2022_B	Italy	129	A: 56.2; F: 0%; R: NR	Spondyloarthritis		
Frede 2023	Germany	150	A: 48.99; F: 40.7%; R: NR	Spondyloarthritis		
Freo 2021	Italy	152	A: 68.45; F: 34.1%; R: NR	Chronic LBP		
Garaigordobil 2016	Spain	88	A:*; F: 96.6%; R: NR	Fibromyalgia		
Garbi 2014	Brazil	60	A: 54.8; F: 63.3%; R: NR	Chronic Back Pain		
GarciaCarrasco 2013	Mexico	51	A: 44.12; F: 100%; R: NR	Irritable Bowel Syndrome	54	A: 43.15; F: 100%; R: NR
Garnæs 2022	Norway	517	A: 45.6; F: 68.5%; R: NR	Chronic Muskuloskeletal Pain	452	A: 42.6; F: 65.7%; R: NR
Gavilan-Carrera 2022	Spain	386	A: 51.2; F: 100%; R: NR	Fibromyalgia		

© 2025 Aaron RV et al. *JAMA Network Open.*

Gebauer 2019	USA	327	A: NR; F: 73.4%; R: 43.4% White, Non-Hispanic; 56.6% Other	Chronic LBP		
Gelonch 2017	Spain	105	A: 45.2; F: 100%; R: NR	Fibromyalgia		
Gelonch 2018	Spain	110	A: 45.4; F: 100%; R: NR	Fibromyalgia		
Gentili 2019	Sweden	252	A: 47.4; F: 81.0%; R: 90% Swedish	Mixed		
Gerdle 2019	Sweden	39916	A: 43.3; F: 72.0%; R: NR	Mixed		
Gerra 2021	Spain	380	A: 51; F: 99.6%; R: 100% Caucasian	Fibromyalgia		
Gisev 2019	Australia	1166	A: 59; F: 57.3%; R: NR	Mixed		
Giummarra 2020	Australia	43789	A: 52; F: 57.9%; R: 74% Oceania/Antarctica; 26% Other	Mixed		
Goesling 2015	NR	1015	A: 50.8; F: 55.5%; R: NR	Mixed		
Goesling 2015_B	NR	551	A: 46.6; F: 66.4%; R: NR	Fibromyalgia		
Goesling 2015b	USA	2104	A: 48.97; F: 59.0%; R: 89.83% White	Mixed		
Goesling 2018	USA	150	A: 48.01; F: 58.7%; R: 88% Caucasian	Mixed		
Gomes 2023	Portugal	634	A: 61.6; F: 78.4%; R: NR	Chronic LBP		
Gorpynchenko 2021	Finland	74	A: NR; F: 0%; R: NR	Chronic Pelvic Pain		
Gota 2017	USA	305	A: 43.5; F: 82.7%; R: 86% White; 9% African American; 1% Hispanic; 1% Asian	Fibromyalgia		
Govind 2020	NR	79	A: NR; F: 100%; R: NR	Chronic Pelvic Pain		
Greenberg 2022	USA	303	A: 59.11; F: 86.1%; R: 5% Hispanic/Latino; 90% Non-Hispanic	Nonmalignant Facial Pain		
Gündüz 2018	Turkey	52	A: 42.65; F: 100%; R: NR	Fibromyalgia	24.5	A: 37.86; F: 100%; R: NR
Gündüz 2018_B	Turkey	35	A: 41.97; F: 100%; R: NR	Myofascial Pain Syndrome	24.5	A: 37.86; F: 100%; R: NR
Gündüz 2019	Turkey	65	A: 33.5; F: 100%; R: NR	Fibromyalgia	52	A: 33.5; F: 100%; R: NR
Gündüz 2019b	Turkey	68	A: 43; F: 100%; R: NR	Fibromyalgia	68	A: 38.5; F: 100%; R: NR
Gupta 2014	India	15	A: 42.8; F: 80%; R: NR	Myofascial Pain Syndrome		
Gupta 2016	India	73	A: 42.29; F: 71.2%; R: NR	Myofascial Pain Syndrome		
Hållstam 2017	Sweden	318	A: 48; F: 64.0%; R: 82.7% Swedish; 6.9% European; 9.1% Non-European; 1.3% Missing	Mixed		
Hamdi 2021	Tunisia	70	A: 51.9; F: 77.0%; R: NR	RA		
Hamdi 2021_B	Tunisia	40	A: 57.5; F: 70%; R: NR	Osteoarthritis		
Han 2019	South Korea	527	A: 66.1; F: 77.8%; R: NR	Chronic Knee Pain	2131	A: 61.2; F: 53.0%; R: NR
Hansdorfer-Korzon 2016	Poland	53	A: 62.73; F: 100%; R: NR	Post Surgical Pain		
Hardy 2022	NR	266	A: 71.8; F: 65.0%; R: NR	Osteoarthritis		
Harrison 2016	UK	221	A: 51; F: 59.0%; R: 94% White; 2% Black; 3% Asian; 1% Other	Mixed		
Hasuo 2020	Japan	152	A: 61.8; F: 80.3%; R: NR	Mixed		

© 2025 Aaron RV et al. *JAMA Network Open.*

Häuser 2014	Germany, USA	167	A: 50.16; F: 15.7%; R: NR	Fibromyalgia		
Häuser 2015	Germany	156	A: 50.26; F: 89.1%; R: 100% Caucasian	Fibromyalgia		
Häuser 2015b	Germany, USA	142	A: 50.95; F: 95.8%; R: 89% Caucasian; 4% Black; 1% Asian; 1% Hispanic; 4% Pacific	Fibromyalgia		
Hoban 2015	Australia	962	A: 59; F: 56.5%; R: 81% Australia; 2% Aboriginal/Torres Strait Islander	Mixed		
Hochman 2013	NR	36	A: 60.7; F: 83.3%; R: 83% Caucasian	Osteoarthritis		
Hogestol 2017	Norway	45	A: 46.6; F: 81.5%; R: 91% Norwegian	Post Surgical Pain	106	
Holloway 2017	NR	491	A: 53.62; F: 95.5%; R: 86% Caucasian; 3% African American; 1% Asian American; 7% Latin/Hispanic/Mexican American; 1% Native American	Fibromyalgia		
Honda 2022	Japan	47	A: 86; F: 71.7%; R: NR	Mixed	45	
Hong 2018	NR	175	A: NR; F: 0%; R: NR	Mixed		
Hudak 2022	USA	149	A: 54.4; F: 61.7%; R: 91% Caucasian; 4% Hispanic; 1% African American; 1% American Indian/Alaska Native; 1% Asian; 1% Native Hawaiian/Pacific Islander; 3% NR	Mixed		
Hughes 2019	USA	313	A: 46.83; F: 63.9%; R: 84% Caucasian; 16% African American; 1% Other	Mixed		
Iannuccelli 2021	Italy	72	A:*; F: 95.8%; R: NR	Fibromyalgia	20	A: NR; F: 85.0%; R: NR
Iannuccelli 2021_B	Italy	82	A:*; F: 84.2%; R: NR	RA	20	A: NR; F: 85.0%; R: NR
Inanc 2014	Turkey	55	A: 50.29; F: 63.6%; R: NR	RA		
Ingegnoli 2022	Italy	124	A: 49; F: 85.2%; R: NR	Rheumatic Muskuloskeletal Disease		
Islam 2023	Australia	126	A: 59.6; F: 77.0%; R: NR	RA		
Islam 2023_B	Australia	57	A: 52.3; F: 43.9%; R: NR	Spondyloarthritis		
Jain 2013	India	100	A: 33; F: 100%; R: NR	RA	100	A: 33.7; F: 100%; R: NR
Jaiswal 2016	NR	61	A: 48.8; F: 49.2%; R: NR	Chronic LBP		
Jaiswal 2016_B	NR	61	A: 50.4; F: 63.9%; R: NR	Mixed		
Janela 2022	USA	296	A: 50.9; F: 52.7%; R: NR	Chronic Shoulder Pain		
Järemo 2022	Sweden	149	A: 46.3; F: 91.0%; R: 88% Sweden; 9% Other Europe; 3% Other	Fibromyalgia		
Jedel 2020	Sweden	56	A: 67.8; F: 100%; R: NR	Burning mouth syndrome	56	A: 67.7; F: 100%; R: NR
Jegan 2017	Germany	423	A: 56.6; F: 57.7%; R: 97% German	Chronic LBP		
Jensen 2016	Denmark	921	A: 46.7; F: 64.0%; R: NR	Mixed		
Jiao 2021	China	124	A: 49.4; F: 86.3%; R: 90% Han	Fibromyalgia		

© 2025 Aaron RV et al. *JAMA Network Open.*

Jimenez-Rodríguez 2014	Spain	44	A: 54.5; F: 93.2%; R: NR	Fibromyalgia	25	A: 50.5; F: 72.0%; R: NR
Jimenez-Rodríguez 2014_B	Spain	32	A: 50.1; F: 56.3%; R: NR	Chronic LBP	25	A: 50.5; F: 72.0%; R: NR
Jin 2021	Ireland	1840	A: 61.72; F: 54.0%; R: NR	Other Arthritis (Mixed, Other, NOS)		
Jin 2021_B	Ireland	2377	A: 61.72; F: 54.0%; R: NR	Chronic Muskuloskeletal Pain		
Jindal 2021	NR	96	A: 49.3; F: 84.4%; R: NR	Chronic LBP		
John 2022	South Korea	612	A: 57.9; F: 54.0%; R: NR	Chronic Spinal Pain		
John 2022_B	South Korea	147	A: 48.3; F: 32.0%; R: NR	CRPS		
John 2022_C	South Korea	113	A: 65.5; F: 44.3%; R: NR	Postherpatic Neuralgia		
Jouini 2014	Canada	486	A: 58.4; F: 67.7%; R: NR	Mixed		
Joyce 2021	USA	320	A: 46; F: 64.0%; R: NR	Chronic LBP		
Kadimpati 2015	USA	595	A: 46.6; F: 70.9%; R: 96% Caucasian	Mixed		
Kallusky 2023	Germany	31	A: 22.8; F: 74.2%; R: NR	Mixed	31	A: 23.1; F: 77.4%; R: NR
Karafin 2018	USA	65	A: 31; F: 66.1%; R: NR	Sickle Cell Disease		
Karas 2020	Turkey	64	A: 39.75; F: 100%; R: NR	Fibromyalgia	58	A: 39.6; F: 100%; R: NR
Kazemi 2013	Iran	16	A: 48.88; F: 0%; R: NR	Phantom Limb Pain		
Kazemi 2013_B	Iran	24	A: 48.63; F: 0%; R: NR	Chronic Neuropathic Pain		
Kec 2022	Czech Republic, Slovakia	347	A: 63.4; F: 44.1%; R: 100% Caucasian	Chronic Neuropathic Pain	432	A: 63.32; F: 46.4%; R: 100% Caucasian
Kha 2020	Denmark	99	A: 47.3; F: 74.8%; R: NR	Mixed		
Khazen 2021	USA	69	A: 59.71; F: 34.8%; R: NR	Mixed		
KibuneNagasako 2016	Brazil	90	A: 48; F: 83.0%; R: NR	Irritable Bowel Syndrome		
Kim 2021	South Korea	65	A: 56.2; F: 20%; R: NR	Post Surgical Pain	35	A: 55; F: 14.3%; R: NR
Knaster 2016	Finland	100	A: 47.9; F: 62.0%; R: NR	Mixed		
Koh 2014	South Korea	80	A: 50.1; F: 0%; R: NR	Chronic Pelvic Pain		
Kosson 2018	Poland	1025	A: 62.15; F: 63.7%; R: NR	Mixed		
Kroenke 2020	USA	153	A: 58.1; F: 8.5%; R: 73% White; 24% Black; 3% Other	Chronic LBP	129	A: 61.7; F: 19.0%; R: 64.3% White; 30.2% Black; 5.4% Other
Kroenke 2020_B	USA	240	A: 58.3; F: 13.3%; R: 86% White; 8% Black; 6% Other	Mixed	129	A: 61.7; F: 19.0%; R: 64.3% White; 30.2% Black; 5.4% Other
Lamerato 2023	USA	194	A: 61.09; F: 64.9%; R: 12% White; 88% Black; 1% Hispanic	Mixed	96	A: 61.09; F: 0%; R: NR
Landefeld 2017	USA	163	A: NR; F: 24.5%; R: NR	Mixed	185	A: NR; F: 21.1%; R: 77.3% Black

© 2025 Aaron RV et al. *JAMA Network Open.*

Larance 2019	Australia	1086	A: 59; F: 66.0%; R: NR	Mixed		
Laurent 2014	France	84	A: 73.5; F: 58.0%; R: NR	Postherpetic Neuralgia		
Laurent 2022	France	49	A: 37; F: 59.7%; R: NR	Mixed	23	
Lee 2014	South Korea	39	A: 37.87; F: 38.5%; R: NR	CRPS		
Lee 2020	USA	51	A: 44.5; F: 63.0%; R: 63% White/European Decent; 8% Black/African American; 16% Latino; 14% Asian/Pacific Islander	Mixed		
León-Suárez 2023	Spain	172	A: NR; F: 0%; R: NR	Other Arthritis (Mixed, Other, NOS)	172	A: 49.6; F: 93.0%; R: NR
Lerman 2015	Israel	428	A: 54.84; F: 56.0%; R: NR	Mixed		
Lin 2017	Taiwan	210	A: 50; F: 32.4%; R: NR	Mixed		
Lindemann 2023	NR	102	A: 64.1; F: 56.0%; R: NR	Lumbar Radiculopathy		
Liu 2018	USA	2976	A: 72; F: 66.2%; R: NR	Osteoarthritis		
Longo 2022	NR	86	A: 74; F: 45.9%; R: NR	Osteoarthritis		
Lopez 2013	USA	345	A: 53.26; F: 0%; R: 49% Caucasian; 11% African American; 9% Hispanic; 2% Other; 29% Unknown	Mixed		
Luciano 2014	Spain	269	A: 52.13; F: 95.5%; R: NR	Fibromyalgia		
Luque-Reca 2021	Spain	230	A: 56.89; F: 100%; R: 100% Spanish	Fibromyalgia		
Mahendru 2021	NR	100	A: 43; F: 90%; R: NR	RA		
Mahlich 2019	Japan	500	A: 54.28; F: 67.0%; R: NR	RA		
Mameli 2014	Italy	14	A: 51.9; F: 100%; R: NR	Fibromyalgia		
Mann 2017	Canada	710	A: 58.81; F: 48.6%; R: NR	Mixed		
Mantyselka 2017	Finland	76	A: 59; F: 54.0%; R: NR	Mixed	285	A: 54.67; F: 48.8%; R: NR
Marcum 2014	USA	190	A: 66.6; F: 15.3%; R: 30% African American; 71% Caucasian	Osteoarthritis		
Marino 2021	Italy	31	A: 42.8; F: 100%; R: NR	Fibromyalgia	31	A: 50; F: 100%; R: NR
Mattila-Rautiainen 2023	Finland	22	A: NR; F: 81.8%; R: NR	Chronic LBP		
Mayer 2013	NR	320	A: 46.5; F: 42.2%; R: 25% African American; 48% Caucasian; 23% Hispanic; 1% Asian; 3% Other/Unknown	Lower Extremity Disorder		
Mayer 2013_B	NR	1844	A: 45.2; F: 37.9%; R: 22% African-American; 53% Caucasian; 20% Hispanic; 2% Asian; 3% Other/Unknown	Chronic LBP		
McCracken 2023	Sweden	560	A: 48.7; F: 93.2%; R: 88% Sweden; 3% Other Scandinavian; 7% Other European; 2% Other	Mixed		
McWilliams 2017	Canada	216	A: 47.06; F: 62.0%; R: NR	Mixed		
Mehraban 2014	Iran	93	A: 46.37; F: 68.8%; R: NR	Mixed		

© 2025 Aaron RV et al. *JAMA Network Open.*

Merlin 2015	USA	30	A: 45; F: 25.0%; R: 63% African American	Mixed	70	
Midbari 2016	Israel	38	A: 33.5; F: 31.6%; R: NR	CRPS		
Miller-Matero 2016	NR	107	A: 37.77; F: 100%; R: 49% Caucasian; 39% African American; 12% Other	Chronic Pelvic Pain		
Moraes 2021	Brazil	81	A: 44.9; F: 50.6%; R: NR	Chronic LBP		
Morlà 2021_B	Spain	68	A: 55.7; F: 47.1%; R: NR	Spondyloarthritis		
Morlà 2021	Spain	102	A: 58.5; F: 82.4%; R: NR	RA		
Muharam 2022	Indonesia	160	A: NR; F: 100%; R: NR	Chronic Pelvic Pain		
Murata 2019	Japan	578	A: 72.8; F: 47.5%; R: NR	Chronic Muskuloskeletal Pain	620	A: 72.8; F: 47.5%; R: NR
MurMartí 2017	Spain	235	A: 54.64; F: 97.8%; R: 99% Caucasian	Fibromyalgia		
Mustonen 2019	Finland	134	A: 60.4; F: 100%; R: NR	Chronic Neuropathic Pain	65	
Nacak 2021	Germany	65	A: 47.5; F: 69.2%; R: 92% German; 5% Other; 3% NR	Mixed	65	A: 43.9; F: 75.4%; R: 96.9% German; 3.1% Other
Neblett 2016	NR	912	A: 45.7; F: 37.0%; R: 57% Caucasian; 23% African American; 18% Hispanic; 2% Other	Post Injury Pain		
Neikrug 2017	USA	292	A: 45.1; F: 93.2%; R: 1.4% Asian; 94.5% White; 0.3% American Indian/Alaskan Native; 3.4% Other; 0.3% Missing	Fibromyalgia		
Nguyen 2021	Vietnam	921	A: 72.6; F: 66.7%; R: NR	Mixed		
Norman-Nott 2022	Wales	3	A: 47.67; F: 100%; R: NR	Mixed		
Offenbaecher 2013	Germany	140	A: 57.8; F: 95.0%; R: NR	Fibromyalgia		
Ojeda 2018	Spain	104	A: 45.6; F: 52.9%; R: NR	Chronic Neuropathic Pain	24	A: 40; F: 56.9%; R: NR
Ojeda 2018_B	Spain	99	A: 47.6; F: 58.6%; R: NR	Chronic Muskuloskeletal Pain	24	A: 40; F: 56.9%; R: NR
Ojeda 2018_C	Spain	51	A: 50.8; F: 96.1%; R: NR	Fibromyalgia	24	A: 40; F: 56.9%; R: NR
Osório 2016	Brazil	50	A: 37.44; F: 100%; R: NR	Chronic Pelvic Pain	50	A: 37.9; F: 100%; R: NR
Parisi 2022	USA	243	A: 53.2; F: 62.6%; R: NR	Mixed		
Pasin 2023	NR	80	A: 42.57; F: 83.8%; R: NR	Myofascial Pain Syndrome		
Peilot 2018	Sweden	42	A: 44; F: 77.8%; R: NR	Mixed		
Penacoba 2023	Spain	268	A: 53.89; F: 100%; R: NR	Fibromyalgia		
Phillips 2014	NR	96	A: 70.6; F: 56.0%; R: NR	Osteoarthritis		
PicchiantiDiamanti 2020	Italy	100	A: 57.7; F: 72.0%; R: NR	Other Arthritis (Mixed, Other, NOS)	100	A: 50.5; F: 59.0%; R: NR
Pinto 2022	NR	78	A: 46; F: 59.0%; R: NR	Chronic LBP	73	A: NR; F: 64.3%; R: NR
Plinsinga 2020	Australia	40	A: 51; F: 95.0%; R: NR	Greater Trochanteric Pain Syndrome	58	A: 53; F: 95.0%; R: NR

© 2025 Aaron RV et al. *JAMA Network Open.*

Plinsinga 2023	Australia, Denmark	693	A: 47; F: 68.0%; R: NR	Mixed		
Porru 2023	NR	42	A: 49.4; F: 100%; R: 99% Caucasian; 1% Asiatic	Chronic Pelvic Pain		
Porru 2023_B	NR	27	A: 49.4; F: 100%; R: 99% Caucasian; 1% Asiatic	Fibromyalgia		
Poulin 2016	Canada	58	A: 46.5; F: 65.5%; R: 79% Caucasian, 2% First Nations, 3% Asian, 5% African; 10% Other	Mixed		
Prateepavanich 2018	Thailand	71	A: 44.83; F: 97.2%; R: 100% Thai	Fibromyalgia		
Preti 2019	Italy	51	A: 48; F: 92.0%; R: NR	Fibromyalgia		
Preti 2019_B	Italy	84	A: 64; F: 68.0%; R: NR	Chronic LBP		
Preti 2019_C	Italy	87	A: 65; F: 76.0%; R: NR	Mixed		
Priol 2023	France	65	A: 73.6; F: 72.3%; R: NR	Osteoarthritis		
Proctor 2013	USA	216	A: 47.8; F: 51.9%; R: 79% White; 17% African American; 2% Hispanic; 1% Middle Eastern; 1% Native American; 1% Other	Mixed		
Puto 2023	Poland	181	A: 77.1; F: 61.9%; R: NR	Mixed		
Quidé 2022	Australia	26	A: 51.64; F: 57.7%; R: NR	Mixed	32	A: 45.29; F: 50%; R: NR
Quinlan 2021	England	60	A: 52.5; F: 52.0%; R: NR	Mixed		
Racine 2014	Canada	88	A: 53.3; F: 66.0%; R: NR	Mixed		
Racine 2017	Canada	728	A: 50.8; F: 61.0%; R: NR	Mixed		
Radat 2013	France	182	A: 59.5; F: 52.2%; R: NR	Chronic Neuropathic Pain		
Rapariz-González 2014	NR	426	A: 58; F: 91.3%; R: NR	Chronic Pelvic Pain	104	A: 69.1; F: 26.0%; R: NR
Rayner 2016	UK	1204	A: 47; F: 66.6%; R: NR	Mixed		
Reiter 2017	Israel	142	A: 37.97; F: 79.0%; R: 100% Israeli	TMD		
Reiter 2017_B	Israel	157	A: 36.88; F: 73.0%; R: 100% Israeli	TMD		
Reiter 2018	Israel	163	A: 36.1; F: 75.0%; R: 100% Israeli	TMD		
Rexelius 2020	Sweden	46	A: 62.5; F: 100%; R: NR	Chronic Pelvic Pain	39	A: NR; F: 100%; R: NR
Rezaei 2014	Iran	100	A: 45.46; F: 72.0%; R: NR	RA		
Rice 2016	Canada	167	A: 44.49; F: 64.2%; R: NR	Mixed		
Rice 2016_B	Canada	163	A: 55.48; F: 76.2%; R: NR	RA		
Robinson 2013	USA	92	A: 48.8; F: 60%; R: 79% Hispanic; 31% Non-Hispanic	Mixed		
Rogal 2015	USA	53	A: 55.2; F: 51.0%; R: 0% Latino; 9% Non-white	Fibromyalgia	140	A: 58.9; F: 36.0%; R: 3% Latino; 9% Non-White
Rogers 2021	Australia	220	A: 52.2; F: 59.6%; R: NR	Chronic plantar heel pain	100	A: 54.75; F: 60%; R: NR
Rojas 2021	USA	27	A: 56.3; F: 48.0%; R: NR	Mixed		

© 2025 Aaron RV et al. *JAMA Network Open.*

Rometsch 2022	Germany	42	A: 52.91; F: 53.2%; R: 96% German; 2% Greek; 2% Italian	Mixed		
Rometsch 2023	Germany	95	A: 46.4; F: 78.1%; R: NR	Mixed		
Rouch 2021	France	234	A: 78.51; F: 72.0%; R: NR	Mixed	459	A: 76.74; F: 53.0%; R: NR
Rouch 2023	Switzerland	1841	A: 60.5; F: 62.9%; R: NR	Mixed	2280	A: 57.4; F: 50.9%; R: NR
Rovner 2017	Sweden	1371	A: 47; F: 68.4%; R: 80% Swedish	Mixed		
Roy 2022	Canada	367	A: 54.61; F: 58.9%; R: 89% Canadian; 12% Other	Mixed		
RusMakovec 2015	Slovenia	81	A: 52.7; F: 70.9%; R: NR	Mixed		
Rusu 2016	UK	78	A: 45.26; F: 70.5%; R: NR	Chronic Back Pain		
Saariaho 2015	Finland	271	A: 47; F: 53.1%; R: NR	Mixed		
Sachau 2023	Germany	60	A: 58.1; F: 43.3%; R: NR	Chronic Neuropathic Pain		
Sagheer 2013	Pakistan	140	A: 43.02; F: 47.1%; R: NR	Chronic LBP		
Saglam 2022	NR	46	A: 39.35; F: 78.3%; R: NR	Lateral epicondylitis	46	A: 38.33; F: 76.1%; R: NR
Santos 2017	Brazil	78	A: 46.65; F: 100%; R: 67% White/Caucasian; 9% Black; 23% Mixed Race	Fibromyalgia	78	A: 46.21; F: 100%; R: 71.8% Caucasian; 15.4% Black; 5.1% Asian; 7.7% Mixed Race
Scherrer 2015	USA	348	A: NR; F: 72.4%; R: NR	Chronic LBP		
Schmukler 2023	Australia, Spain, USA	393	A: 58.9; F: 51.4%; R: NR	RA		
Schmukler 2023_B	Australia, Spain, USA	111	A: 54.1; F: 51.4%; R: NR	Spondyloarthritis		
Schmukler 2023_C	Australia, Spain, USA	64	A: 49.3; F: 43.8%; R: NR	Spondyloarthritis		
Schroeter 2015	Germany	85	A: 53.55; F: 59.8%; R: NR	Chronic Muskuloskeletal Pain		
Schroeter 2015_B	Germany	89	A: 53.55; F: 59.8%; R: NR	Osteoarthritis		
Schwab 2022	Germany	274	A:*; F: 100%; R: NR	Chronic Pelvic Pain		
Schwarm 2021	NR	39	A: 61; F: 46.2%; R: NR	Chronic LBP		
Seed 2015	Malaysia	83	A: 50.4; F: 56.6%; R: 32.5% Malay; 12.1% Chinese; 54.2% Indian; 1.2% Other	Mixed		
Seekatz 2016	Germany	360	A: 49.86; F: 64.2%; R: NR	Chronic Back Pain		
Sener 2013	Turkey	39	A: 42; F: 100%; R: NR	Fibromyalgia	40	A: 38.3; F: 100%; R: NR
Sengupta 2023	India	145	A: NR; F: 60%; R: NR	Chronic Muskuloskeletal Pain		
Shamji 2016	Canada	150	A: 54; F: 57.0%; R: NR	Chronic Neuropathic Pain		
Sharma 2022	England	129	A: NR; F: 0%; R: NR	Fibromyalgia		
Shebeshi 2023	Australia	84829	A: 51.84; F: 58.1%; R: 71% Australian; 29% Other	Mixed		

© 2025 Aaron RV et al. *JAMA Network Open.*

Shmagel 2016	USA	700	A:*; F: 55.8%; R: 10% African American; 75% White; 12% Hispanic; 3% Other	Chronic LBP	4403	A: NR; F: 50.1%; R: 12.1% African American; 64.9% White; 14.9% Hispanic; 8.1% Other
Si 2019	China	501	A: 69.56; F: 75.6%; R: NR	Mixed	718	A: 68.89; F: 64.8%; R: NR
Silva 2021	Portugal	106	A: NR; F: 0%; R: NR	Fibromyalgia		
Siqueira-Campos 2019	Brazil	100	A: NR; F: 100%; R: NR	Chronic Pelvic Pain	100	A: NR; F: 100%; R: NR
Siqueira-Campos 2022	Brazil	123	A: 37; F: 100%; R: 25.2% White; 74.8% Non-White	Chronic Pelvic Pain	123	A: 31.9; F: 100%; R: 20.3% White; 79.7% Non-White
Sitges 2018	Spain	35	A: 52.37; F: 100%; R: NR	Fibromyalgia	18	A: 50.89; F: 100%; R: NR
Slawek 2021	USA	185	A: 53.9; F: 55.1%; R: 37% White; 26% Hispanic/Latinx; 30% Black; 7% Other	Mixed		
Sleurs 2020	USA	731	A:*; F: 87.5%; R: 80% White; 5% Black; 4% Native American; 3% Asian; 7% Hispanic	Fibromyalgia	35,578	A: NR; F: 51.2%; R: 65.9% White; 11.9% Black; 1.5% Native American; 5.8% Asian; 14.9% Hispanic
Sohn 2016	South Korea	32	A: 36.88; F: 40.6%; R: NR	CRPS		
Song 2015	USA	143	A: 65.5; F: 58.0%; R: 87% Caucasian	Osteoarthritis		
Soriano-Maldonado 2015	Spain	451	A: 52; F: 100%; R: NR	Fibromyalgia		
Souza 2021	Brazil	30	A: 49; F: 43.3%; R: NR	Spondyloarthritis		
Sparkes 2015	UK	56	A: 47.4; F: 55.4%; R: NR	Chronic Neuropathic Pain		
Stefani 2019	Brazil	88	A: 64.55; F: 100%; R: NR	Osteoarthritis		
Stefani 2019_B	Brazil	36	A: 37.22; F: 100%; R: NR	Chronic Pelvic Pain		
Stefani 2019_C	Brazil	117	A: 49.34; F: 100%; R: NR	Fibromyalgia		
Stehlik 2018	Sweden	31	A: 57; F: 100%; R: NR	Fibromyalgia	23	A: 57; F: 100%; R: NR
Stein 2015	USA	159	A: 40.68; F: 43.4%; R: NR	Mixed	169	A: 36.9; F: 43.2%; R: NR
Steiner 2017	NR	216	A: 45.81; F: 95.8%; R: 88% Caucasian; 9% African American; 1% American Indian; 1% Pacific Islander; 1% Other	Fibromyalgia		
Stokholm 2022	Denmark	51	A: 50; F: 55.4%; R: NR	Limb-Girdle Muscular Dystrophy		A: 50; F: 0%; R: NR
Subramaniam 2013	Singapore	282	A:*; F: 18.0%; R: 17% Indian; 15% Chinese; 13% Malay; 24% Other	Other Arthritis (Mixed, Other, NOS)		
SundaraRajan 2017	Canada	54	A: 46.11; F: 48.2%; R: NR	Chronic Neuropathic Pain		
Sundstrom 2023	Sweden	404	A: 47.75; F: 93.8%; R: NR	Mixed		
Tanaka 2022	Japan	91	A: 79.36; F: 40.7%; R: NR	Mixed		
Tardif 2023	Australia, New Zealand	23915	A: 48.3; F: 55.6%; R: 75% Australia; 25% Other	Mixed		
Tatebe 2016	NR	38	A: 43; F: 52.6%; R: NR	Chronic Wrist Pain		

© 2025 Aaron RV et al. *JAMA Network Open.*

Taylor 2021	NR	941	A: 47.7; F: 94.8%; R: 95.9% White; 1.6% Mixed; 1.0% Asian; 0.6% Black; 0.1% Other; 0.4% NR	Fibromyalgia		
Teixido-Abiol 2022	Spain	64	A: 60.35; F: 70%; R: NR	Chronic Neuropathic Pain		
Teixido-Abiol 2022_B	Spain	64	A: 60.35; F: 70%; R: NR	Mixed		
Terassi 2020	Brazil	186	A: 68.9; F: 80.6%; R: NR	Mixed		
Tetsunaga 2013	NR	122	A: 58; F: 63.1%; R: NR	Chronic LBP	64	A: 60; F: 20.3%; R: NR
Teychenne 2019	Australia	40	A: 34.7; F: 47.5%; R: NR	Chronic LBP		
Thakral 2018	USA	1570	A: 62; F: 63.5%; R: NR	Mixed		
ThiNguy 2022	Taiwan	22	A: 49.5; F: 95.5%; R: NR	Fibromyalgia	22	A: 47.6; F: 100%; R: NR
Thomas 2022	USA	96	A: 35.34; F: 100%; R: NR	Chronic Pelvic Pain		
Thompson 2023	USA	501	A: 54.27; F: 95.4%; R: NR	Fibromyalgia		
Tocchetto 2023	Brazil	83	A: 48.67; F: 100%; R: NR	Fibromyalgia	18	A: 46.06; F: 100%; R: NR
Tsuji 2016	Japan	425	A: 53.9; F: 44.0%; R: NR	Chronic LBP		
Uebelacker 2015	USA	131	A: 50.82; F: 42.0%; R: 59% Caucasian; 27% Black/African American; 2% Native American; 4% Other; 15% Hispanic/Latino	Mixed	107	A: 51; F: 32.0%; R: 62% Caucasian; 31% Black; 3% Native American; 5% Other; 20% Hispanic/Latino
Uniyal 2017_B	India	55	A: 31.56; F: 41.8%; R: 100% South-Asian	Chronic LBP	27.5	A: 29.67; F: 40%; R: 100% South-Asian
Upadhyaya 2023	India	138	A: 47.17; F: 73.9%; R: NR	RA		
Upadhyaya 2023_B	India	62	A: 51.37; F: 90.3%; R: NR	Mixed		
Vance 2018	USA	143	A: 50.2; F: 100%; R: NR	Fibromyalgia		
vandenBerk-Clark 2017	USA	41	A: 52.9; F: 61.0%; R: 51% White; 49% African American	Mixed		
vanEeden 2023	Canada	30	A: 44.3; F: 100%; R: NR	Fibromyalgia	30	A: 44.7; F: 66.7%; R: NR
VanOvermeire 2022	Belgium	103	A: NR; F: 100%; R: NR	Fibromyalgia		
VanRyckeghem 2013	Belgium	73	A: 49.95; F: 65.8%; R: NR	Mixed		
Varinen 2019	Finland	515	A: NR; F: 85.0%; R: NR	Fibromyalgia	11409	A: NR; F: 61.9%; R: NR
Vilalta-Abella 2015	NR	19	A: NR; F: 0%; R: NR	Fibromyalgia		
Villafaina 2019	Spain	28	A: 54.96; F: 100%; R: NR	Fibromyalgia		
Voute 2023	France	329	A: 51.4; F: 75.7%; R: NR	Mixed		
Vukojević 2022	Bosnia and Herzegovina	64	A: 46.9; F: 45.3%; R: NR	Chronic LBP		
Wadley 2019	South Africa	50	A: 45; F: 88.0%; R: 100% South African	Post Injury Pain		
Wadley 2020	South Africa	63	A: NR; F: 0%; R: 100% South African	Mixed	551	
Wahlman 2014	Finland	232	A: 61.67; F: 68.0%; R: NR	Mixed		
Weingarten 2016	USA	48	A: NR; F: 100%; R: NR	Fibromyalgia		

© 2025 Aaron RV et al. *JAMA Network Open.*

Whitlock 2017	USA	1120	A: 73.8; F: 76.0%; R: 86% White; 8% Black; 5% Hispanic; 1% Other	Mixed	8945	A: 73.6; F: 58.1%; R: 85.6% White; 8% Black; 4.5% Hispanic; 1.9% Other
Widenka 2021	NR	60	A: 81.6; F: 78.3%; R: NR	Osteoarthritis		
Wildes 2020	USA	150	A: NR; F: 68.7%; R: 80% White; 8% American Indian/Alaska Native; 4% Black; 1% Asian American; 7% Multiracial/Other	Mixed		
Wilson 2015	NR	92	A: 49.3; F: 78.0%; R: NR	Mixed		
Wilson 2022	USA	93	A: 40.58; F: 80%; R: 81.7% White Non-Hispanic; 18.3% Non-White and/or Hispanic	Mixed		
Wolfe 2013	Germany	52	A: 59.8; F: 60.8%; R: NR	Fibromyalgia	2393	A: 50.2; F: 53.5%; R: NR
Wong 2017	USA	131	A: 55; F: 67.9%; R: NR	Mixed		
Wright 2017	UK	21	A: 53.62; F: 79.2%; R: NR	Mixed		
Xu 2020	China	3039	A:*; F: 49.5%; R: NR	Mixed		
Yadav 2023	USA	223	A: 52.13; F: 50.9%; R: 5% Asian; 7.95% Black; 4.4% Other; 82.6% White	Chronic Pancreatitis	519	A: 52.13; F: 0%; R: NR
Yamada 2022	Brazil	108	A: 46.2; F: 56.5%; R: 20% White; 18% Black	Chronic LBP		
Yin 2023	China	78	A: 59.21; F: 88.5%; R: NR	Chronic Musculoskeletal Pain		
Yu 2021	UK	555	A: 40; F: 86.3%; R: 92% White; 4% Mixed; 2% Asian; 1% Black; 1% Other	Mixed		
Zaidel 2021	USA	4201	A: NR; F: 67.3%; R: NR	Mixed		
Zakrzewska 2017	UK	225	A: 60.9; F: 63.6%; R: 11% Asian; 84% Anglo-American; 6% Other	Trigeminal Neuralgia		
Zambelli 2021	UK	1059	A: 42.88; F: 88.0%; R: 94% White; 1% Black/Black British; 1% Asian/Asian British; 2% Mixed; 2% Other	Mixed		
Zubatsky 2020	USA	320	A: NR; F: 71.3%; R: NR	Chronic LBP		

Note. *Indicates data reported categorically versus mean or median. Study IDs that end in _B or _C indicate distinct pain groups within a larger study. Mixed conditions also includes studies where pain condition was not reported. RA = Rheumatoid Arthritis; TMD = Temporal mandibular disorder; CRPS = complex regional pain syndrome; MOGAD = Myelin oligodendrocyte glycoprotein antibody-associated disease; Chronic LBP = Chronic Low Back Pain

© 2025 Aaron RV et al. *JAMA Network Open.*

eTable 2. Overview of Symptom Measures and Diagnostic Assessments Used

	Depression Symptoms		Anxiety Symptoms		Depression Diagnosis	Anxiety Diagnosis
Study ID	Measure	Cutoff	Measure	Cutoff		
Aagaard 2023	PHQ-9	10			PRIME-MD	
Abrams 2013	CES-D	16				
Ahmad 2023	HADS-D	11				
Ajo 2017	HADS-D	11	HADS-A	11		
Åkerblom 2020	HADS-D	11	HADS-A	11		
Akui 2022	Zung SDS	16	STAI-T	40		
Alamam 2019	DASS-21	14	DASS-21	10		
Alberts 2020	PHQ-8	10	GAD-7	10		
Alciati 2018					SCID-I-CV	
Alciati 2020					SCID-I	SCID-I
Alhalal 2021	CES-D	16				
Allaire 2018	PHQ-9	10	GAD-7	10		
Al-Maharbi 2018	PHQ-9	6				
Aloush 2021	PHQ-9	10	GAD-7	10		
AlShukaili 2022	PHQ-9	12				
AlvesRodrigues 2022	BDI-II	20	BAI	11		
Amatsu 2022	GDS-15	5				
Amital 2014	BDI-II	20				
Ammitzbøll 2021	PHQ-9	10				
Andersen 2016	HADS-D	8	HADS-A	8		
Annagür 2014					SCID-I	SCID-I
Antaky 2017	HADS-D	11				
Antunes 2013	BDI	16.5				
Ardigo 2016	HADS-D	8	HADS-A	8		
Areias 2023	PHQ-9	5	GAD-7	5		
Argoff 2016	HADS-D	11	HADS-A	11		
Asada 2022	GDS-15	6				
Asada 2022_B		6				
Aşçıbaşı 2022	HADS-D	8	HADS-A	11		
Asih 2014	BDI-II	20				
Asih 2016					SCID-I	SCID-I
Asseyer 2021	BDI-II	14				
Ataoglu 2013	BDI	20				
Avluk 2014	HAM-D	16				
Bagnato 2014	HAM-D	18				
Bailey 2020	PHQ-9	5	GAD-7	5		
Bapir 2023			GAD-7	5		
Barbosa 2016	HADS-D	8	HADS-A	8		
Barjandi 2021	PHQ-9	10	GAD-7	10		

© 2025 Aaron RV et al. *JAMA Network Open.*

Barjandi 2021_B	PHQ-9	10	GAD-7	10		
Barth 2014	CES-D	10				
Barton 2021	PHQ-8	10				
Bayrak 2020	HDRS	7	BAI	8		
Bayram 2014	HADS-D	8	HADS-A	11		
Bayram 2014_B	HADS-D	8	HADS-A	11		
Bell 2016	PHQ-9	10				
Bilen 2022	BDI	19	BAI	16		
Birch 2022	HADS-D	11	HADS-A	11		
Blakey 2018					SCID-I	
Bohn 2013	PHQ-2	3				
Botros 2022	PHQ-8	5	GAD-7	5		
Boukhira 2021	HADS-D	11	HADS-A	11		
Bravo 2019	HADS-D	11	HADS-A	11		
BromleyMilton 2013	HADS-D	11	HADS-A	11		
Bruce 2021	CES-D	16				
Brunner 2023	PHQ-8	10				
Bryant 2016	HADS-D	11	HADS-A	11		
Bucourt 2021					MINI	MINI
Bucourt 2021_B					MINI	MINI
Bucourt 2021_C					MINI	MINI
Calandre 2022	PHQ-9	10				
Calderón 2016	HADS-D	8	HADS-A	8		
Calpin 2017	HADS-D	11	HADS-A	11		
Campbell 2015					CIDI	CIDI
Campbell 2015b	PHQ-9	10	GAD-7	10		
Campos 2021	HADS-D	8	HADS-A	8		
Cappe 2021	HADS-D	10	HADS-A	10		
Carleton 2022	PHQ-9	10	GAD-7	10		
Carrillo-de-la-Peña 2015	BDI	18				
Carrillo-Izquierdo 2018	PHQ-9	10				
Carta 2018					ANTAS	ANTAS
CasteloAzevedo 2021	CIS-R	2				
Caumo 2022					MINI	MINI
Chaabouni 2022	HADS-D	10	HADS-A	10		
Chahal-Kummen 2019	HADS-D	8	HADS-A	8		
Chahal-Kummen 2023	HADS-D	8	HADS-A	8		
Challa 2017	PHQ-9	10				
Chana 2021	PHQ-9	10	GAD-7	10		
Chang 2018	GDS-30	10	HAM-A	18		
Cheatle 2023					MINI	
Chen 2022	PHQ-8	10	GAD-2	3		
Cheng 2018	CES-D Brief	12				

© 2025 Aaron RV et al. *JAMA Network Open.*

Cheng 2022	PROMIS CAT	59.9	PROMIS CAT	62.3		
Cho 2017	HADS-D	8	HADS-A	8		
Choi 2018	BDI	20				
Chytas 2023	BDI-II	21				
Ciaramella 2015					MINI Plus	MINI Plus
Ciaramella 2015_B					MINI Plus	MINI Plus
Civelek 2022	GDS-30	11				
Clark 2018			STAI-T	39		
Clark 2019			STAI-T	39		
Cody 2019	PHQ-2	3				
Conway 2020	PHQ-9	11	GAD-7	11		
Corriger 2022	HADS-D	11	HADS-A	11		
Costa 2023	HADS-D	8	HADS-A	8		
Csupak 2018						CIDI
Csupak 2018_B						CIDI
DaLuz 2018	HADS-D	8	HADS-A	8		
Damci 2022	HADS-D	11	HADS-A	11		
Davis 2014	CES-D	27				
Davis 2014_B	CES-D	27				
Dear 2023	PHQ-9	10	GAD-7	10		
deHeer 2017	HADS-D	8	HADS-A	8		
DeLaRosa 2023	PHQ-8	10	GAD-7	10		
DelPozo-Cruz 2017	BDI	17				
deMoraesVieira 2014	BDI	16				
Depintor 2016	HADS-D	9	HADS-A	9		
DeRoa 2018	HADS-D	11	HADS-A	11		
Doherty 2017	BDI-PC	5				
Domenech 2013	HADS-D	11	HADS-A	11		
DosSantosProença 2023	PHQ-9	11	GAD-7	10		
Dougados 2017	HADS-D	11	HADS-A			
Drummond 2013	DASS-21	88th Percentile	DASS-21	88th Percentile		
Drummond 2013_B	DASS-21	24	DASS-21	88th Percentile		
D'Souza 2020	PHQ-9	10	GAD-7	10		
Dubois-Mendes 2021	HADS-D	9	HADS-A	8		
Dumolard 2023	HADS-D	11	HADS-A	11		
Durán 2021					CIDI Short Form	
Ecija 2022	HADS-D	13	HADS-A	13		
Edit 2013	BDI	19				
Elrashidi 2018	PHQ-9	10	GAD-7	10		
Emery 2014					PRIME-MD	PRIME-MD
Englbrecht 2019	PHQ-9	10				
Eriksen 2021	MDI	25				
Falling 2021	HADS-D	8	HADS-A	8		

© 2025 Aaron RV et al. *JAMA Network Open.*

Farin 2015	HADS-D	12	HADS-A	12		
Farzad 2021	DASS-21	28	DASS-21	20		
Feingold 2017	PHQ-9	10	GAD-7	10		
Feingold 2018	PHQ-9	10	GAD-7	10		
Feingold 2020	PHQ-9	10	GAD-7	10		
Feliu-Soler 2017	CES-D	20				
Fiegl 2019	CES-D	22			ICF Checklist	
Filippon 2013					MINI	
Finn 2018	HADS-D	9	HADS-A	9		
Fischer-Jbali 2022					SCID-I	SCID
Flores-Curiel 2023	BDI	17				
Foti 2022	BDI-II	14				
Foti 2022_B	BDI-II	14				
Frede 2023	PHQ-9	10				
Freo 2021	HADS-D	8	HADS-A	8		
Garaigordobil 2016	BDI	18				
Garbi 2014	BDI	21				
GarciaCarrasco 2013	CES-D	16				
Garnæs 2022	HADS-D	8	HADS-A	8		
Gavilan-Carrera 2022	BDI-II	29				
Gebauer 2019	PHQ-2	3				
Gelonch 2017	BDI-II	20				
Gelonch 2018	BDI-II	14				
Gentili 2019			GAD-7	10		
Gerdle 2019	HADS-D	11	HADS-A	11		
Gerra 2021	BDI	14				
Gisev 2019	PHQ-9	10	GAD-7	10		
Giummarra 2020	DASS-21	14	DASS-21	10		
Goesling 2015	HADS-D	11				
Goesling 2015_B	HADS-D	11				
Goesling 2015b	HADS-D	11				
Goesling 2018	HADS-D	11				
Gomes 2023	HADS-D	15				
Gorpynchenko 2021	PHQ-9	10	GAD-7	10		
Gota 2017	PHQ-9	10				
Govind 2020	PHQ-8	10				
Greenberg 2022	PROMIS (8 item)	55	PROMIS (8 item)	55		
Gündüz 2018					SCID-I	SCID-I
Gündüz 2018_B					SCID-I	SCID-I
Gündüz 2019					SCID-I	SCID-I
Gündüz 2019b					SCID-I	SCID-I
Gupta 2014	BDI	17				
Gupta 2016	BDI	17				

© 2025 Aaron RV et al. *JAMA Network Open.*

Hållstam 2017	HADS-D	8	HADS-A	8		
Hamdi 2021	BDI	16	BAI	25		
Hamdi 2021_B	BDI	16	BAI	25		
Han 2019	PHQ-9	10				
Hansdorfer-Korzon 2016	HADS-D	8	HADS-A	8		
Hardy 2022	BDI	8	STAI-T	57		
Harrison 2016	PHQ-9	11				
Hasuo 2020	HADS-D	8	HADS-A	8		
Häuser 2014	PHQ-2	3				
Häuser 2015					ICF Checklist	ICF Checklist
Häuser 2015b	PHQ-2	3	GAD-2	3		
Hoban 2015	PHQ-9	11	GAD-7	11		
Hochman 2013	CES-D	16				
Hogestol 2017	HADS-D	12	HADS-A	12		
Holloway 2017	CES-D	16				
Honda 2022	GDS-15	6				
Hong 2018	PHQ-9	10				
Hudak 2022					MINI	
Hughes 2019	BDI-II	20				
Iannuccelli 2021	Zung SDS	48	Zung SAS	60		
Iannuccelli 2021_B	Zung SDS	48	Zung SAS	60		
Inanc 2014	HADS-D	7	HADS-A	10		
Ingegnoli 2022	PHQ-9	15				
Islam 2023	HADS-D	8	HADS-A	8		
Islam 2023_B	HADS-D	8	HADS-A	8		
Jain 2013					MADRS	
Jaiswal 2016	PHQ-9	15				
Jaiswal 2016_B	PHQ-9	15				
Janela 2022	PHQ-9	5	GAD-7	5		
Järemo 2022	HADS-D	11	HADS-A	11		
Jedel 2020	HADS-D	7	HADS-A	7		
Jegan 2017	HADS-D	11				
Jensen 2016	HADS-D	11	HADS-A	11		
Jiao 2021	BDI-II	8				
Jimenez-Rodríguez 2014	BDI	18				
Jimenez-Rodríguez 2014_B	BDI	18				
Jin 2021	CES-D	16				
Jin 2021_B	CES-D	16				
Jindal 2021	Zung SDS (Modified)	33				
John 2022	PHQ-9	10				
John 2022_B	PHQ-9	10				
John 2022_C	PHQ-9	10				
Jouini 2014	HADS-D	11	HADS-A	11		

© 2025 Aaron RV et al. *JAMA Network Open.*

Joyce 2021	PHQ-8	10	GAD-7	10		
Kadimpati 2015	CES-D	20				
Kallusky 2023	BDI-II	20	HADS-A	8		
Karafin 2018	PHQ-9	10	GAD-7	10		
Karas 2020	BDI	14	BAI	16		
Kazemi 2013	HADS-D	11	HADS-A	11		
Kazemi 2013_B	HADS-D	11	HADS-A	11		
Kec 2022	HADS-D	8	HADS-A	8		
Kha 2020					PRIME-MD	
Khazen 2021	BDI	19				
KibuneNagasako 2016	HADS-D	9	HADS-A	9		
Kim 2021	HADS-D	11	HADS-A	11		
Knaster 2016					SCID-I	
Koh 2014	PHQ-9	10				
Kosson 2018	HADS-D	11	HADS-A	11		
Kroenke 2020					SCID (version NR)	
Kroenke 2020_B					SCID (version NR)	
Lamerato 2023	PHQ-2	3				
Landefeld 2017	CES-D	16				
Larance 2019	PHQ-9	10	GAD-7	10		
Laurent 2014	HADS-D	10	HADS-A	10		
Laurent 2022	HADS-D	8	HADS-A	8		
Lee 2014					SCID-I	SCID-1
Lee 2020	PHQ-9	11	GAD-7	11		
León-Suárez 2023	HADS-D	9	HADS-A	9		
Lerman 2015	CES-D	19				
Lin 2017	BDI	19				
Lindemann 2023	HADS-D	8	HADS-A	8		
Liu 2018	PHQ-8	10				
Longo 2022	GDS-30	10				
Lopez 2013	BDI-II	20				
Luciano 2014	HADS-D	11	HADS-A	11		
Luque-Reca 2021	HADS-D	11	HADS-A	11		
Mahendru 2021	DASS-42	10	DASS-42	8		
Mahlich 2019	PHQ-9	10				
Mameli 2014					SCID-I	SCID-I
Mann 2017	PHQ-9	10				
Mantyselka 2017	BDI	10				
Marcum 2014	CES-D	16				
Marino 2021	HAM-D	22	HAM-A	18		
Mattila-Rautiainen 2023	BDI	20				
Mayer 2013					SCID-I	
Mayer 2013_B					SCID-I	

© 2025 Aaron RV et al. *JAMA Network Open.*

McCracken 2023	PHQ-9	10	GAD-7	10		
McWilliams 2017	PHQ-9	10				
Mehraban 2014					SCID-I	SCID-I
Merlin 2015	PHQ-9	10				
Midbari 2016	BDI	22				
Miller-Matero 2016	HADS-D	9	HADS-A	9		
Moraes 2021	HADS-D	9	HADS-A	9		
Morlà 2021_B	HADS-D	8				
Morlà 2021	HADS-D	8				
Muharam 2022					MINI	MINI
Murata 2019	GDS-15	6				
MurMartí 2017	HADS-D	11	HADS-A	11		
Mustonen 2019	BDI-II	10	HADS-A	8		
Nacak 2021					SCID-I	
Neblett 2016	BDI	10				
Neikrug 2017					SCID (version NR)	SCID (version NR)
Nguyen 2021	GDS-15	6				
Norman-Nott 2022	BDI	19				
Offenbaecher 2013	HADS-D	9	HADS-A	9		
Ojeda 2018	HADS-D	10	HADS-A	10		
Ojeda 2018_B	HADS-D	10	HADS-A	10		
Ojeda 2018_C	HADS-D	10	HADS-A	10		
Osório 2016					SCID-I-CV	SCID-I-CV
Parisi 2022					MINI	MINI
Pasin 2023			BAI	16		
Peilot 2018	BDI	19	BAI	16		
Penacoba 2023	HADS-D	12	HADS-A	12		
Phillips 2014	HADS-D	11	HADS-A	11		
PicchiantiDiamanti 2020	DASS-21	10	DASS-21	8		
Pinto 2022	HADS-D	9	HADS-A	9		
Plinsinga 2020	HADS-D	8	HADS-A	8		
Plinsinga 2023	PHQ-9	15	GAD-7	10		
Porru 2023	PHQ-9	15				
Porru 2023_B	PHQ-9	15				
Poulin 2016	PHQ-9	10	GAD-7	10		
Prateepavanich 2018	HAM-D	14				
Preti 2019	PHQ-9	10				
Preti 2019_B	PHQ-9	10				
Preti 2019_C	PHQ-9	10				
Priol 2023	BDI	8	HADS-A	11		
Proctor 2013					CAPPE	CAPPE
Puto 2023	GDS-15	6				
Quidé 2022	BDI	19				

© 2025 Aaron RV et al. *JAMA Network Open.*

Quinlan 2021	PHQ-9	11				
Racine 2014						
Racine 2017	BDI	19				
Radat 2013					MINI	MINI
Rapariz-González 2014			Goldberg Anxiety Scale	4		
Rayner 2016	PHQ-9	15				
Reiter 2017	PHQ-9	10	GAD-7	10		
Reiter 2017_B	PHQ-9	10	GAD-7	10		
Reiter 2018	PHQ-9	10	GAD-7	10		
Rexelius 2020	MADRS	13				
Rezaei 2014	HADS-D	11				
Rice 2016	DASS-21	7	DASS-21	8		
Rice 2016_B	DASS-21	7	DASS-21	8		
Robinson 2013	BDI-II	14				
Rogal 2015	HADS-D	8	HADS-A	8		
Rogers 2021	PHQ-9	11				
Rojas 2021	CES-D	19				
Rometsch 2022	DASS-21	10	DASS-21	6		
Rometsch 2023	PHQ-9	11	GAD-7	11		
Rouch 2021	CES-D	17 men, 23 women				
Rouch 2023					DIGS	DIGS
Rovner 2017	HADS-D	11	HADS-A	11		
Roy 2022	PHQ-9	15				
RusMakovec 2015	Zung SDS	60	Zung SAS	60		
Rusu 2016	HADS-D	11			SCID-I	
Saariaho 2015	BDI-II	20				
Sachau 2023	HADS-D	8	HADS-A	8		
Sagheer 2013	HADS-D	11	HADS-A	11		
Saglam 2022	BDI	17				
Santos 2017					MINI	MINI
Scherrer 2015	PHQ-2	3				
Schmukler 2023	MDHAQ-Dep	1				
Schmukler 2023_B	MDHAQ-Dep	1				
Schmukler 2023_C	MDHAQ-Dep	1				
Schroeter 2015					SCID-I	SCID-I
Schroeter 2015_B					SCID-I	SCID-I
Schwab 2022	PHQ-2	3	GAD-2	3		
Schwarm 2021	HADS-D	8	HADS-A	8		
Seed 2015					MINI	
Seekatz 2016	PHQ-9	10				
Sener 2013	BDI	17	BAI	16		
Sengupta 2023	GDS-15	5				
Shamji 2016	BDI	10	BAI	8		

© 2025 Aaron RV et al. *JAMA Network Open.*

Sharma 2022	PHQ-9	11	GAD-7	11		
Shebeshi 2023			DASS-21	14		
Shmagel 2016	PHQ-9	10				
Si 2019	GDS-5	2				
Silva 2021	HADS-D	11	HADS-A	11		
Siqueira-Campos 2019	HADS-D	8	HADS-A	8		
Siqueira-Campos 2022	PHQ-9	10	GAD-7	10		
Sitges 2018	BDI-II	29				
Slawek 2021	PHQ-9	10	GAD-7	10		
Sleurs 2020					AUDADIS-5	AUDADIS-5
Sohn 2016					SCID-I	SCID-I
Song 2015	CES-D Brief	10				
Soriano-Maldonado 2015	BDI-II	20				
Souza 2021	RDC/TMD	0.535				
Sparkes 2015	HADS-D	11	HADS-A	11		
Stefani 2019	BDI	19				
Stefani 2019_B	HDRS	18				
Stefani 2019_C	HDRS	18				
Stehlik 2018	HADS-D	10	HADS-A	10		
Stein 2015	PHQ-2	3				
Steiner 2017	PHQ-8	5				
Stokholm 2022	HADS-D	8	HADS-A	8		
Subramaniam 2013					CIDI	CIDI
SundaraRajan 2017	HADS-D	8	HADS-A	8		
Sundstrom 2023	PHQ-9	8				
Tanaka 2022	HADS-D	8	HADS-A	8		
Tardif 2023	DASS-21	14	DASS-21	10		
Tatebe 2016	Zung SDS	40				
Taylor 2021	HADS-D	11	HADS-A	11		
Teixido-Abiol 2022	HAM-D	13	HAM-A	15		
Teixido-Abiol 2022_B	HAM-D	13	HAM-A	15		
Terassi 2020	GDS-15	11				
Tetsunaga 2013	Zung SDS	40				
Teychenne 2019	CES-D Brief	10				
Thakral 2018	PHQ-8	10				
ThiNguy 2022	BDI-II	20	BAI	19		
Thomas 2022	PHQ-9	10	GAD-7	10		
Thompson 2023	CES-D	19				
Tocchetto 2023	HDRS	18			MINI	MINI
Tsuji 2016	PHQ-9	10				
Uebelacker 2015	CES-D Brief	10				
Uniyal 2017_B	PHQ-9	10	GAD-7	10		
Upadhyaya 2023	HADS-D	11	HADS-A	11		

© 2025 Aaron RV et al. *JAMA Network Open.*

Upadhyaya 2023_B	HADS-D	11	HADS-A	11		
Vance 2018	PROMIS (version NR)	61	PROMIS (version NR)	61		
vandenBerk-Clark 2017	PHQ-9	15	BAI-PC	5		
vanEeden 2023	HADS-D	11	HADS-A	11		
VanOvermeire 2022	PHQ-2	3				
VanRyckeghem 2013	HADS-D	13				
Varinen 2019	BDI	19				
Vilalta-Abella 2015	HADS-D	12				
Villafaina 2019	GDS-15	6				
Voute 2023	HADS-D	11	HADS-A	11		
Vukojević 2022	HAM-D	9	HAM-A	19		
Wadley 2019	BDI-II	20				
Wadley 2020	PHQ-9	10				
Wahlman 2014	DEPS	12				
Weingarten 2016	PHQ-9	15	GAD-7	10		
Whitlock 2017	CES-D	4				
Widenka 2021	GDS-15	11				
Wildes 2020	PHQ-8	10	GAD-7	10		
Wilson 2015	PHQ-8	15				
Wilson 2022	PROMIS (8 item)	23				
Wolfe 2013	PHQ-2	3	GAD-2	3		
Wong 2017	Zung SDS	60				
Wright 2017	PHQ-9	15				
Xu 2020					CIDI	CIDI
Yadav 2023	PROMIS-29	61	PROMIS-29	61		
Yamada 2022	HADS-D	9	HADS-A	9		
Yin 2023	GDS-15	6				
Yu 2021	PHQ-9	10				
Zaidel 2021	PHQ-2	3				
Zakrzewska 2017	HADS-D	9	HADS-A	9		
Zambelli 2021	HADS-D	11				
Zubatsky 2020	PHQ-2	3				

Note: BDI = Beck Depression Inventory; BDI-PC = BDI-Primary Care; CES-D = Center for Epidemiological Studies Depression Scale; CIS-R = Clinical Interview Schedule-Revised; DASS = Depression, Anxiety, and Stress Scale; DEPS = Depression Scale; GDS = Geriatric Depression Scale; HADS-D = Hospital Anxiety and Depression Scale-Depression Subscale; HAM-D = Hamilton Depression Rating Scale; HDRS = Hamilton Depression Rating Scale; MADRS = Montgomery-Åsberg Depression Rating Scale; MDHAQ-Dep = Multidimensional Health Assessment Questionnaire-Depression; MDI = Major Depression Inventory; PHQ = Patient Health Questionnaire; PROMIS = Patient-Reported Outcomes Measurement Information System; PROMIS CAT = PROMIS Computerized Adaptive Test; RDC/TMD = Research Diagnostic Criteria for Temporomandibular Disorders; Zung SDS = Zung Self-Rating Depression Scale; BAI = Beck Anxiety Inventory; BAI-PC = BAI-Primary Care; CAPPE = Community Assessment of Psychic Experiences-Positive Scale; CIDI = Composite International Diagnostic Interview; GAD = Generalized Anxiety Disorder Assessment; HADS-A = Hospital Anxiety and Depression Scale-Anxiety Subscale; HAM-A = Hamilton Anxiety Rating Scale; STAI-T = State-Trait Anxiety Inventory-Trait Version; Zung SAS = Zung Self-Rating Anxiety Scale; ANTAS = Advanced Neuropsychiatric Tools and

© 2025 Aaron RV et al. *JAMA Network Open.*

Assessment Schedule; AUDADIS = Alcohol Use Disorders and Associated Disabilities Interview Schedule; DIGS = Diagnostic Interview for Genetic Studies; ICF = International Classification of Functioning, Disability, and Health; MINI = Mini International Neuropsychiatric Interview; PRIME-MD = Primary Care Evaluation of Mental Disorders; SCID = Structured Clinical Interview for DSM Disorders; SCID-I-CV = Structured Clinical Interview for DSM Disorders-Clinical Version.

© 2025 Aaron RV et al. *JAMA Network Open.*

eFigure 1. Prevalence of Clinical Symptoms of Depression, Forest Plots

Study name	Event rate	Lower limit	Upper limit
Plinsinga 2020	0.034	0.006	0.163
Terassi 2020	0.054	0.029	0.097
Bilen 2022	0.055	0.025	0.117
Rogers 2021	0.064	0.038	0.105
Hogestol 2017	0.073	0.025	0.195
Porru 2023_B	0.074	0.019	0.252
Bayrak 2020	0.081	0.055	0.117
Zaidel 2021	0.083	0.075	0.092
Varinen 2019	0.088	0.066	0.116
Gomes 2023	0.096	0.075	0.121
Amital 2014	0.100	0.028	0.303
Widenka 2021	0.100	0.046	0.205
Chaabouni 2022	0.102	0.043	0.223
Challa 2017	0.108	0.061	0.184
Mahlich 2019	0.110	0.085	0.141
Wadley 2020	0.110	0.053	0.214
Koh 2014	0.113	0.060	0.202
Garnæs 2022	0.113	0.088	0.143
Rice 2016_B	0.117	0.076	0.176
Chahal-Kummen 2019	0.120	0.058	0.231
Quidé 2022	0.120	0.040	0.308
Sagheer 2013	0.121	0.076	0.186
Prateepavanich 2018	0.127	0.067	0.226
Davis 2014	0.130	0.055	0.278
Jin 2021	0.131	0.116	0.147
Foti 2022_B	0.132	0.084	0.202
Falling 2021	0.136	0.044	0.348
Ammitzbøll 2021	0.138	0.097	0.193
Hochman 2013	0.139	0.059	0.293
Choi 2018	0.150	0.094	0.232
Ardigo 2016	0.151	0.077	0.274
Elrashidi 2018	0.154	0.130	0.181
Chahal-Kummen 2023	0.155	0.099	0.235
Jin 2021_B	0.158	0.144	0.173
Asada 2022	0.160	0.126	0.201
Domenech 2013	0.160	0.100	0.247
Rouch 2021	0.160	0.118	0.213
Gorpynchenko 2021	0.162	0.094	0.264
Dougados 2017	0.163	0.118	0.223
Tsuji 2016	0.165	0.132	0.203
Chen 2022	0.165	0.127	0.212
Si 2019	0.168	0.138	0.203
Farin 2015	0.170	0.129	0.220
Farzad 2021	0.170	0.104	0.266
Iannuccelli 2021_B	0.171	0.104	0.268
Han 2019	0.173	0.143	0.208
Cheng 2022	0.175	0.154	0.198
Hardy 2022	0.177	0.136	0.228
Kosson 2018	0.179	0.156	0.203
Reiter 2017_B	0.180	0.127	0.248
Stehlik 2018	0.185	0.084	0.360
DeLaRosa 2023	0.186	0.177	0.195
Birch 2022	0.188	0.062	0.447
Porru 2023	0.190	0.098	0.336
Rojas 2021	0.190	0.082	0.380
SundaraRajan 2017	0.190	0.106	0.316
Penacoba 2023	0.192	0.149	0.244
Murata 2019	0.192	0.162	0.227
Kallusky 2023	0.194	0.090	0.369
Mann 2017	0.196	0.168	0.227

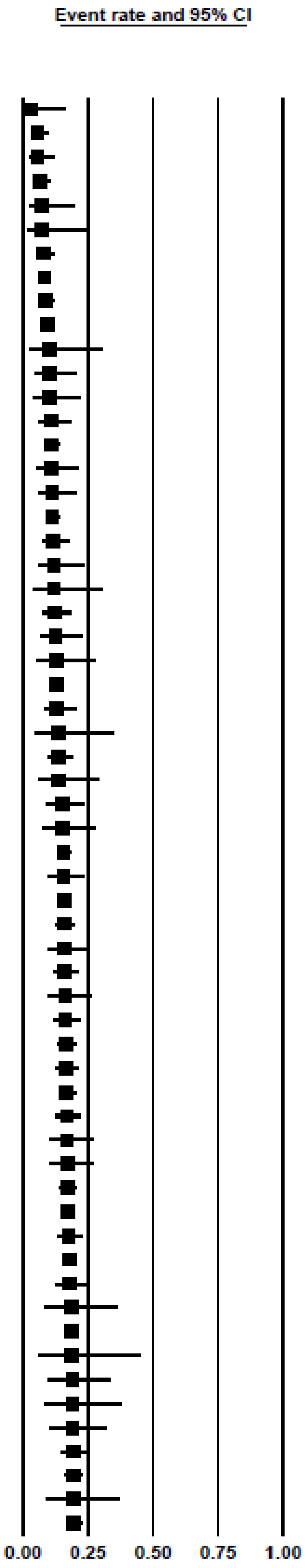

© 2025 Aaron RV et al. *JAMA Network Open.*

Study name	Event rate	Lower limit	Upper limit
Asada 2022_B	0.199	0.148	0.262
Priol 2023	0.200	0.120	0.315
Feingold 2020	0.201	0.152	0.261
Reiter 2018	0.202	0.148	0.271
Barton 2021	0.208	0.158	0.269
Englbrecht 2019	0.209	0.185	0.236
Liu 2018	0.209	0.195	0.224
Calpin 2017	0.210	0.141	0.301
Bailey 2020	0.211	0.203	0.219
Phillips 2014	0.213	0.142	0.306
Song 2015	0.217	0.157	0.292
Shmagel 2016	0.218	0.189	0.250
Goesling 2015	0.221	0.196	0.247
Cheng 2018	0.221	0.191	0.254
Jegan 2017	0.222	0.185	0.264
Laurent 2022	0.224	0.129	0.361
Civelek 2022	0.227	0.142	0.343
Damci 2022	0.230	0.208	0.254
Plinsinga 2023	0.230	0.200	0.263
Hamdi 2021	0.233	0.149	0.346
Areias 2023	0.234	0.201	0.271
VanRyckeghem 2013	0.234	0.151	0.344
CasteloAzevedo 2021	0.235	0.204	0.269
Pinto 2022	0.237	0.155	0.343
Jouini 2014	0.239	0.203	0.279
Foti 2022	0.240	0.182	0.310
vandenBerk-Clark 2017	0.244	0.137	0.397
Khazen 2021	0.246	0.159	0.361
Frede 2023	0.250	0.187	0.325
Jedel 2020	0.250	0.154	0.379
Sachau 2023	0.250	0.157	0.374
Antaky 2017	0.251	0.201	0.308
Luciano 2014	0.253	0.205	0.308
Ojeda 2018_B	0.253	0.177	0.348
Argoff 2016	0.256	0.237	0.275
Rusu 2016	0.256	0.172	0.364
Miller-Matero 2016	0.257	0.183	0.348
AlShukaili 2022	0.260	0.213	0.314
John 2022	0.260	0.227	0.296
Mantyselka 2017	0.260	0.174	0.370
Alamam 2019	0.261	0.189	0.349
Rexelius 2020	0.261	0.155	0.405
Schmukler 2023	0.262	0.221	0.308
Ecija 2022	0.263	0.210	0.324
Freo 2021	0.266	0.201	0.341
Janela 2022	0.267	0.220	0.320
Sparkes 2015	0.268	0.169	0.398
Bryant 2016	0.269	0.209	0.339
DosSantosProença 2023	0.269	0.193	0.362
Wilson 2022	0.270	0.190	0.369
BromleyMilton 2013	0.271	0.258	0.285
John 2022_C	0.274	0.200	0.363
Stein 2015	0.277	0.213	0.351
Ojeda 2018	0.279	0.201	0.373
Dubois-Mendes 2021	0.280	0.192	0.389
Edit 2013	0.280	0.202	0.375
Vukojevi? 2022	0.281	0.185	0.403
AlvesRodrigues 2022	0.283	0.172	0.428
Hansdorfer-Korzon 2016	0.283	0.178	0.418
Bell 2016	0.291	0.280	0.302

Event rate and 95% CI

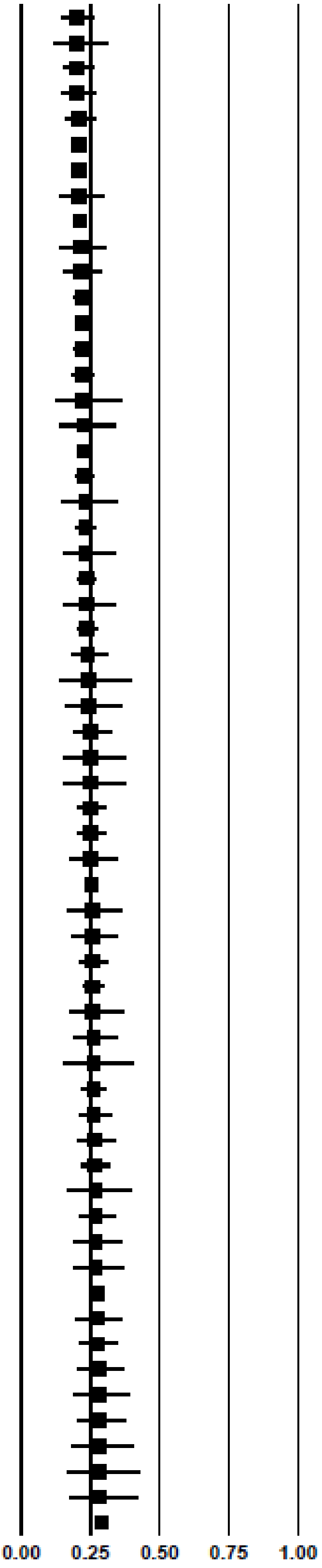

© 2025 Aaron RV et al. *JAMA Network Open.*

Study name	Event rate	Lower limit	Upper limit
Jensen 2016	0.293	0.265	0.323
Merlin 2015	0.300	0.164	0.483
Stokholm 2022	0.300	0.191	0.438
Ataoglu 2013	0.302	0.223	0.394
Govind 2020	0.304	0.213	0.414
Vance 2018	0.310	0.240	0.390
Zakrzewska 2017	0.311	0.254	0.375
Rovner 2017	0.313	0.289	0.338
Saariaho 2015	0.314	0.261	0.371
Yamada 2022	0.315	0.234	0.408
Marcum 2014	0.316	0.254	0.385
Thomas 2022	0.316	0.231	0.415
Schmukler 2023_C	0.317	0.215	0.440
ThiNguy 2022	0.318	0.160	0.534
Mattila-Rautiainen 2023	0.318	0.160	0.534
Ajo 2017	0.319	0.243	0.406
Goesling 2018	0.320	0.250	0.399
Järemo 2022	0.320	0.250	0.399
Upadhyaya 2023	0.320	0.248	0.402
Antunes 2013	0.321	0.259	0.390
Chana 2021	0.324	0.193	0.491
Morlà 2021	0.324	0.240	0.420
Stefani 2019_C	0.325	0.246	0.415
deMoraesVieira 2014	0.326	0.267	0.391
León-Suárez 2023	0.326	0.260	0.400
Allaire 2018	0.328	0.275	0.387
RusMakovec 2015	0.330	0.237	0.439
Depintor 2016	0.333	0.268	0.405
Wildes 2020	0.333	0.262	0.412
Norman-Nott 2022	0.333	0.043	0.846
Thakral 2018	0.337	0.314	0.361
Wahlman 2014	0.340	0.282	0.403
Lin 2017	0.342	0.281	0.409
Jindal 2021	0.344	0.256	0.444
Jaiswal 2016_B	0.344	0.236	0.471
Barbosa 2016	0.346	0.230	0.484
Inanc 2014	0.350	0.236	0.484
Islam 2023_B	0.351	0.239	0.482
Roy 2022	0.352	0.304	0.402
Tanaka 2022	0.352	0.261	0.455
Gerdle 2019	0.352	0.347	0.357
Joyce 2021	0.353	0.303	0.407
Karafin 2018	0.356	0.249	0.479
Islam 2023	0.357	0.278	0.444
Sener 2013	0.359	0.225	0.519
Yadav 2023	0.359	0.299	0.424
Alhalal 2021	0.360	0.301	0.424
Zambelli 2021	0.360	0.332	0.389
Jaiswal 2016	0.361	0.251	0.488
Goesling 2015b	0.362	0.342	0.383
Kec 2022	0.363	0.314	0.415
Bagnato 2014	0.365	0.275	0.465
Bayram 2014_B	0.367	0.216	0.549
Schmukler 2023_B	0.367	0.283	0.460
Marino 2021	0.368	0.219	0.548
Davis 2014_B	0.370	0.267	0.487
Wilson 2015	0.370	0.278	0.473
Calderón 2016	0.375	0.259	0.508
Kazemi 2013	0.375	0.179	0.623
Corriger 2022	0.376	0.318	0.438

Event rate and 95% CI

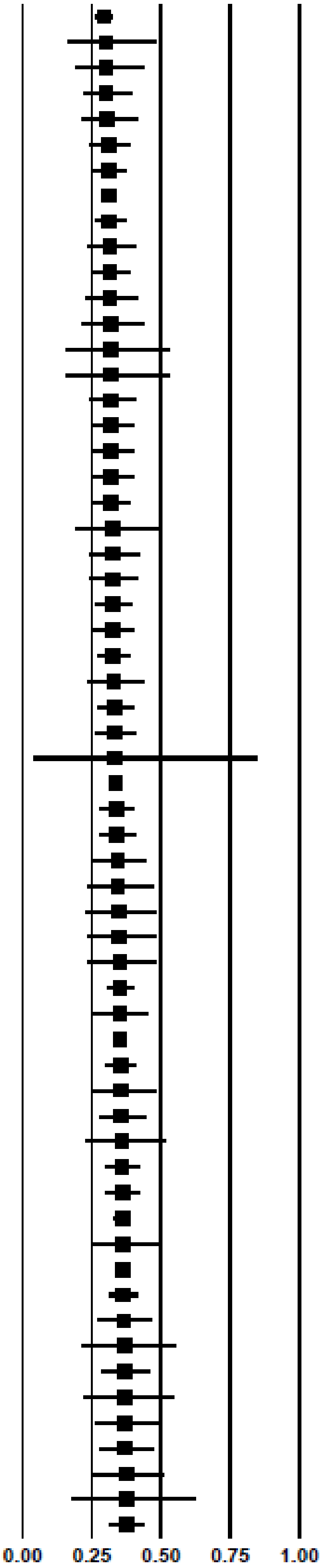

© 2025 Aaron RV et al. *JAMA Network Open.*

Study name	Event rate	Lower limit	Upper limit
Drummond 2013_B	0.380	0.183	0.627
Seekatz 2016	0.380	0.331	0.431
Voute 2023	0.381	0.330	0.435
Flores-Curiel 2023	0.388	0.297	0.487
Asih 2014	0.392	0.340	0.446
Ojeda 2018_C	0.392	0.269	0.531
Vilalta-Abella 2015	0.395	0.207	0.621
Luque-Reca 2021	0.400	0.339	0.465
Weingarten 2016	0.400	0.272	0.543
Holloway 2017	0.403	0.360	0.447
Gavilan-Carrera 2022	0.409	0.361	0.459
Dumolard 2023	0.410	0.352	0.470
Jimenez-Rodríguez 2014_B	0.410	0.256	0.584
GarciaCarrasco 2013	0.411	0.285	0.549
Saglam 2022	0.413	0.281	0.559
Barjandi 2021	0.416	0.342	0.494
Laurent 2014	0.417	0.316	0.524
PicchiantiDiamanti 2020	0.420	0.327	0.519
Tardif 2023	0.421	0.414	0.427
deHeer 2017	0.421	0.364	0.480
Feingold 2017	0.422	0.390	0.455
Teychenne 2019	0.425	0.283	0.580
Morlà 2021_B	0.426	0.315	0.545
Conway 2020	0.430	0.369	0.494
Drummond 2013	0.430	0.248	0.633
Lee 2020	0.430	0.302	0.568
Teixido-Abiol 2022_B	0.430	0.315	0.553
McCracken 2023	0.431	0.391	0.472
Lamerato 2023	0.433	0.365	0.504
Larance 2019	0.437	0.408	0.467
Hoban 2015	0.440	0.409	0.472
Lindemann 2023	0.440	0.347	0.537
Offenbaecher 2013	0.440	0.360	0.523
Gisev 2019	0.444	0.416	0.473
VanOvermeire 2022	0.447	0.354	0.544
Mustonen 2019	0.450	0.368	0.535
Peilot 2018	0.450	0.308	0.600
Hållstam 2017	0.456	0.402	0.511
Robinson 2013	0.456	0.357	0.558
Cody 2019	0.460	0.416	0.505
Slawek 2021	0.460	0.389	0.532
Hughes 2019	0.463	0.409	0.519
Eriksen 2021	0.464	0.292	0.645
Häuser 2015b	0.465	0.384	0.547
Brunner 2023	0.465	0.400	0.531
Campbell 2015b	0.466	0.441	0.491
vanEeden 2023	0.466	0.299	0.641
Hasuo 2020	0.467	0.389	0.546
Schwab 2022	0.467	0.409	0.526
Åkerblom 2020	0.468	0.392	0.546
Amatsu 2022	0.473	0.363	0.586
Thompson 2023	0.473	0.430	0.517
Kim 2021	0.476	0.358	0.596
Teixido-Abiol 2022	0.480	0.361	0.601
Wadley 2019	0.480	0.346	0.617
Ingegnoli 2022	0.485	0.398	0.573
Costa 2023	0.485	0.391	0.581
Honda 2022	0.489	0.351	0.629
Preti 2019_B	0.490	0.385	0.596
Preti 2019_C	0.490	0.387	0.594

Event rate and 95% CI

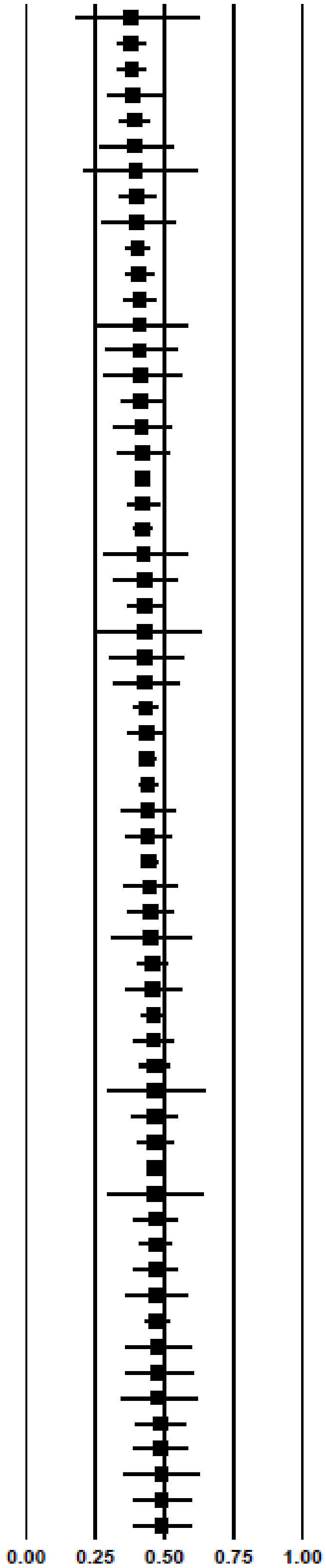

© 2025 Aaron RV et al. *JAMA Network Open.*

Study name	Event rate	Lower limit	Upper limit
Alberts 2020	0.492	0.373	0.612
Andersen 2016	0.496	0.409	0.583
Akui 2022	0.500	0.294	0.706
Iannuccelli 2021	0.500	0.387	0.613
Kazemi 2013_B	0.500	0.310	0.690
Racine 2017	0.500	0.464	0.536
Rometsch 2022	0.500	0.353	0.647
Tatebe 2016	0.500	0.346	0.654
Whitlock 2017	0.500	0.471	0.529
Wong 2017	0.500	0.415	0.585
Taylor 2021	0.506	0.474	0.538
Bravo 2019	0.513	0.360	0.664
Yin 2023	0.513	0.403	0.622
Sitges 2018	0.514	0.353	0.672
Abrams 2013	0.520	0.458	0.581
Avluk 2014	0.523	0.377	0.664
Cappe 2021	0.530	0.416	0.641
MurMartí 2017	0.532	0.468	0.595
Bayram 2014	0.533	0.358	0.700
Jiao 2021	0.533	0.445	0.619
Schwarm 2021	0.538	0.383	0.686
Chang 2018	0.538	0.452	0.622
Mahendru 2021	0.540	0.442	0.635
Reiter 2017	0.540	0.458	0.620
Silva 2021	0.540	0.445	0.632
Uniyal 2017_B	0.546	0.414	0.672
A?ç?ba?? 2022	0.548	0.424	0.667
Hong 2018	0.549	0.474	0.621
Barth 2014	0.550	0.494	0.605
Rayner 2016	0.551	0.523	0.579
Carrillo-de-la-Peña 2015	0.558	0.422	0.686
Gebauer 2019	0.560	0.505	0.613
Harrison 2016	0.560	0.494	0.624
Feingold 2018	0.563	0.521	0.604
Rice 2016	0.563	0.487	0.636
Nguyen 2021	0.565	0.533	0.597
Lopez 2013	0.568	0.515	0.619
Poulin 2016	0.569	0.440	0.689
Doherty 2017	0.570	0.473	0.662
DeRoa 2018	0.571	0.423	0.707
Villafaina 2019	0.571	0.386	0.738
KibuneNagasako 2016	0.577	0.473	0.675
Scherrer 2015	0.578	0.525	0.628
Zubatsky 2020	0.578	0.523	0.631
Tocchetto 2023	0.578	0.470	0.679
Carleton 2022	0.579	0.557	0.600
Chytas 2023	0.586	0.502	0.664
Goesling 2015_B	0.586	0.545	0.627
Rometsch 2023	0.588	0.487	0.682
Asseyer 2021	0.591	0.382	0.772
Gota 2017	0.597	0.541	0.651
Dear 2023	0.598	0.571	0.625
Midbari 2016	0.605	0.444	0.746
Longo 2022	0.607	0.500	0.704
Stefani 2019_B	0.611	0.446	0.754
Landefeld 2017	0.615	0.538	0.687
Garbi 2014	0.617	0.489	0.730
Wright 2017	0.619	0.402	0.797
DelPozo-Cruz 2017	0.620	0.524	0.708
Finn 2018	0.625	0.565	0.680

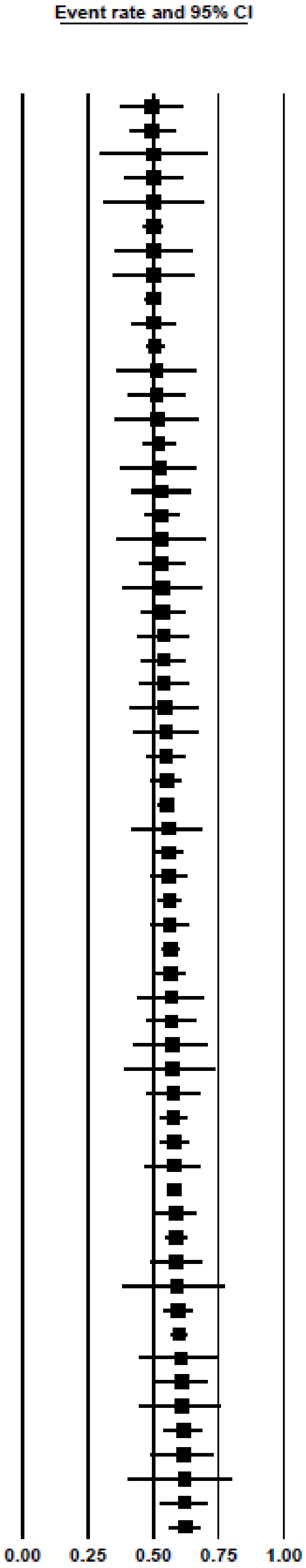

© 2025 Aaron RV et al. *JAMA Network Open.*

Study name	Event rate	Lower limit	Upper limit
DaLuz 2018	0.630	0.532	0.719
Hamdi 2021_B	0.630	0.473	0.764
Moraes 2021	0.630	0.520	0.728
Shamji 2016	0.630	0.550	0.703
Siqueira-Campos 2019	0.630	0.532	0.719
Wolfe 2013	0.635	0.497	0.754
Garaigordobil 2016	0.636	0.531	0.730
Puto 2023	0.640	0.568	0.707
Giummarra 2020	0.653	0.649	0.657
Lerman 2015	0.660	0.614	0.703
Rezaei 2014	0.660	0.562	0.746
Rogal 2015	0.660	0.524	0.774
Fiegl 2019	0.663	0.621	0.704
Greenberg 2022	0.670	0.615	0.721
Gelonch 2017	0.676	0.581	0.758
Uebelacker 2015	0.679	0.595	0.754
Kadimpati 2015	0.690	0.652	0.726
Ahmad 2023	0.698	0.599	0.781
Soriano-Maldonado 2015	0.701	0.657	0.741
John 2022_B	0.702	0.623	0.770
Sundstrom 2023	0.705	0.659	0.748
D'Souza 2020	0.709	0.671	0.744
Al-Maharbi 2018	0.710	0.643	0.769
Aagaard 2023	0.714	0.540	0.842
Karas 2020	0.719	0.597	0.815
Häuser 2014	0.731	0.658	0.792
Siqueira-Campos 2022	0.732	0.647	0.803
Bohn 2013	0.735	0.648	0.807
Sengupta 2023	0.738	0.660	0.803
Tetsunaga 2013	0.770	0.687	0.836
McWilliams 2017	0.773	0.712	0.824
Barjandi 2021_B	0.778	0.675	0.855
Bruce 2021	0.790	0.758	0.819
Neblett 2016	0.792	0.764	0.817
Gerra 2021	0.792	0.748	0.830
Aloush 2021	0.800	0.743	0.847
Yu 2021	0.800	0.765	0.831
Botros 2022	0.802	0.758	0.840
Boukhira 2021	0.804	0.757	0.844
Carrillo-Izquierdo 2018	0.805	0.748	0.852
Stefani 2019	0.807	0.711	0.876
Cho 2017	0.820	0.732	0.884
Preti 2019	0.820	0.690	0.903
Quinlan 2021	0.820	0.702	0.898
Sharma 2022	0.822	0.746	0.879
Campos 2021	0.826	0.746	0.885
Feliu-Soler 2017	0.844	0.779	0.892
Calandre 2022	0.854	0.809	0.891
Gelonch 2018	0.860	0.782	0.913
Gupta 2014	0.867	0.595	0.967
Jimenez-Rodríguez 2014	0.910	0.783	0.966
Gupta 2016	0.918	0.829	0.963
Upadhyaya 2023_B	0.950	0.858	0.983
Souza 2021	0.967	0.798	0.995
Steiner 2017	0.968	0.934	0.985
Pooled estimate	0.393	0.373	0.414
Prediction interval	0.393	0.119	0.756

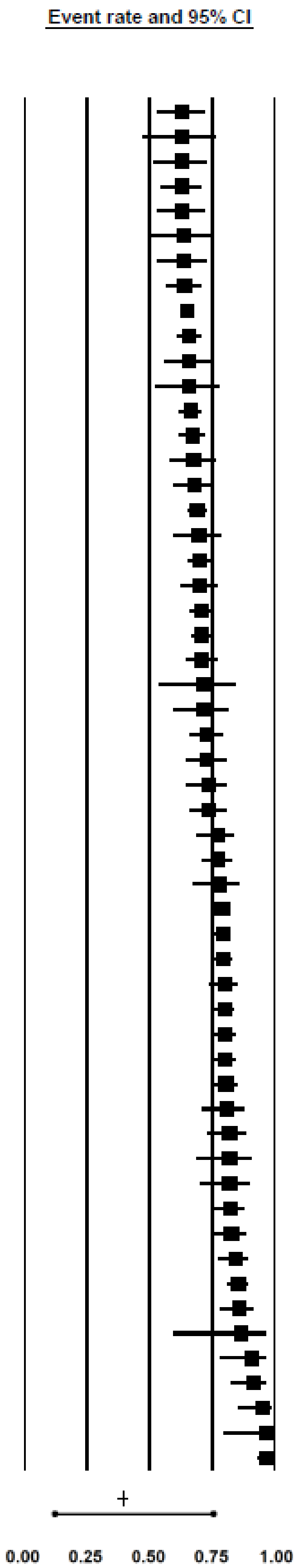

© 2025 Aaron RV et al. *JAMA Network Open.*

eFigure 2. Prevalence of Clinical Symptoms of Anxiety, Forest Plots

Study name	Event rate	Lower limit	Upper limit
Chaabouni 2022	0.020	0.003	0.131
Hardy 2022	0.038	0.021	0.069
Hogestol 2017	0.063	0.020	0.183
Reiter 2017_B	0.100	0.062	0.158
Freo 2021	0.101	0.062	0.160
Bilen 2022	0.109	0.063	0.182
Reiter 2018	0.129	0.085	0.189
Elrashidi 2018	0.144	0.121	0.170
Iannuccelli 2021_B	0.146	0.085	0.240
Rice 2016_B	0.147	0.100	0.210
DeLaRosa 2023	0.151	0.143	0.160
Lindemann 2023	0.160	0.101	0.244
Hamdi 2021	0.162	0.093	0.267
Sagheer 2013	0.164	0.111	0.235
Upadhyaya 2023	0.170	0.116	0.242
Chen 2022	0.172	0.133	0.219
Drummond 2013_B	0.190	0.063	0.450
Wildes 2020	0.193	0.138	0.264
RusMakovec 2015	0.197	0.124	0.298
Hamdi 2021_B	0.200	0.103	0.352
Gorpynchenko 2021	0.203	0.126	0.309
Plinsinga 2023	0.210	0.181	0.242
Larance 2019	0.214	0.191	0.239
Gisev 2019	0.215	0.192	0.240
Kim 2021	0.215	0.132	0.331
Chana 2021	0.219	0.113	0.382
Hoban 2015	0.219	0.194	0.246
Feingold 2020	0.225	0.173	0.286
Campbell 2015b	0.228	0.208	0.250
Shamji 2016	0.230	0.170	0.304
Allaire 2018	0.235	0.188	0.290
Pinto 2022	0.237	0.155	0.343
Chahal-Kummen 2023	0.240	0.169	0.328
Inanc 2014	0.240	0.145	0.370
Mustonen 2019	0.250	0.184	0.330
Plinsinga 2020	0.250	0.140	0.405
Gentili 2019	0.250	0.200	0.307
Vukojevi? 2022	0.250	0.159	0.370
Kazemi 2013	0.250	0.097	0.508
Tanaka 2022	0.253	0.174	0.352
Cheng 2022	0.253	0.229	0.278
Areias 2023	0.259	0.224	0.297
Clark 2019	0.260	0.199	0.332
Farin 2015	0.260	0.210	0.316
McCracken 2023	0.264	0.229	0.302
Damci 2022	0.265	0.242	0.290
Barjandi 2021_B	0.267	0.182	0.374
Garnæs 2022	0.279	0.242	0.319
SundaraRajan 2017	0.280	0.177	0.413
Bailey 2020	0.283	0.274	0.292
Calpin 2017	0.290	0.210	0.386
Karafin 2018	0.292	0.195	0.413
Janela 2022	0.294	0.245	0.348
Domenech 2013	0.300	0.217	0.398
Joyce 2021	0.303	0.255	0.356
Phillips 2014	0.306	0.222	0.405
BromleyMilton 2013	0.307	0.293	0.321
Priol 2023	0.308	0.208	0.430
Teixido-Abiol 2022_B	0.310	0.209	0.433
Lee 2020	0.310	0.199	0.449
Penacoba 2023	0.318	0.265	0.376
Farzad 2021	0.320	0.229	0.427

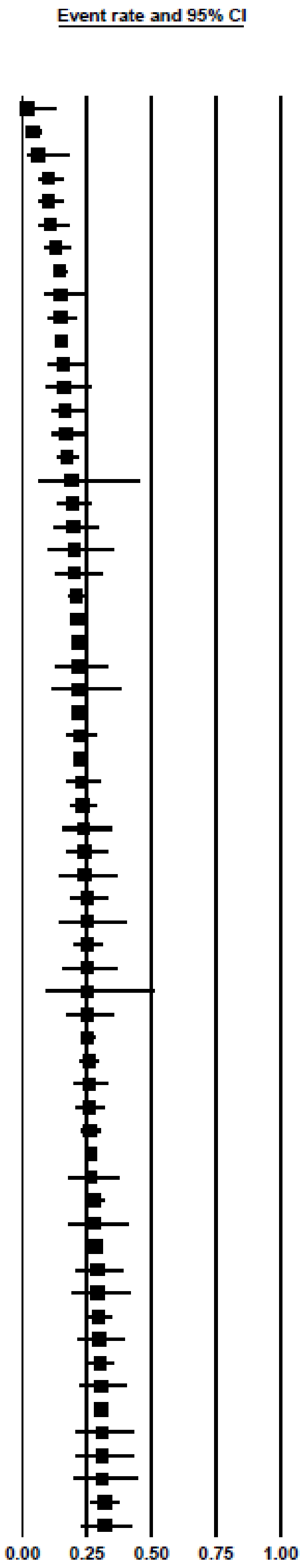

© 2025 Aaron RV et al. *JAMA Network Open.*

Study name	Event rate	Lower limit	Upper limit
Ardigo 2016	0.321	0.210	0.457
Kosson 2018	0.324	0.296	0.353
Stehlik 2018	0.330	0.189	0.510
Järemo 2022	0.330	0.259	0.409
Chahal-Kummen 2019	0.330	0.222	0.460
Conway 2020	0.330	0.273	0.392
Sachau 2023	0.333	0.226	0.461
A?ç?ba?? 2022	0.339	0.233	0.465
Thomas 2022	0.348	0.260	0.448
Drummond 2013	0.350	0.186	0.559
Slawek 2021	0.350	0.285	0.421
Yadav 2023	0.350	0.290	0.415
Rovner 2017	0.353	0.328	0.379
Chang 2018	0.354	0.276	0.440
DosSantosProença 2023	0.356	0.270	0.452
Sparkes 2015	0.357	0.243	0.490
Rometsch 2023	0.366	0.276	0.467
Bayram 2014_B	0.367	0.216	0.549
Iannuccelli 2021	0.367	0.264	0.484
Ecija 2022	0.368	0.308	0.432
Islam 2023_B	0.368	0.254	0.499
Feingold 2017	0.370	0.339	0.402
Argoff 2016	0.372	0.351	0.393
Birch 2022	0.375	0.179	0.623
Calderón 2016	0.375	0.259	0.508
Ojeda 2018	0.375	0.287	0.472
Kec 2022	0.375	0.326	0.427
Depintor 2016	0.376	0.309	0.449
PicchiantiDiamanti 2020	0.380	0.290	0.479
Stokholm 2022	0.380	0.258	0.519
Laurent 2014	0.381	0.284	0.489
Barjandi 2021	0.383	0.311	0.460
Miller-Matero 2016	0.386	0.299	0.481
Jensen 2016	0.390	0.359	0.421
Vance 2018	0.390	0.314	0.472
Alamam 2019	0.390	0.305	0.482
Ajo 2017	0.391	0.310	0.479
Ojeda 2018_B	0.394	0.303	0.493
Gerdle 2019	0.395	0.390	0.400
Bayrak 2020	0.397	0.344	0.453
Jouini 2014	0.399	0.356	0.443
Uniyal 2017_B	0.400	0.280	0.533
Dear 2023	0.406	0.379	0.433
Rometsch 2022	0.409	0.272	0.561
Teixido-Abiol 2022	0.410	0.297	0.533
Islam 2023	0.413	0.330	0.501
Alberts 2020	0.415	0.302	0.537
Dubois-Mendes 2021	0.427	0.323	0.538
Poulin 2016	0.431	0.311	0.560
Tardif 2023	0.433	0.427	0.440
Schwarm 2021	0.436	0.291	0.593
Weingarten 2016	0.440	0.308	0.581
Peilot 2018	0.440	0.299	0.591
Hasuo 2020	0.447	0.370	0.527
Häuser 2015b	0.451	0.371	0.533
Zakrzewska 2017	0.453	0.389	0.519
D'Souza 2020	0.454	0.414	0.494
Falling 2021	0.454	0.264	0.658
ThiNguy 2022	0.455	0.265	0.659
Corriger 2022	0.461	0.400	0.524
Jedel 2020	0.464	0.339	0.594
Laurent 2022	0.469	0.335	0.608

Event rate and 95% CI

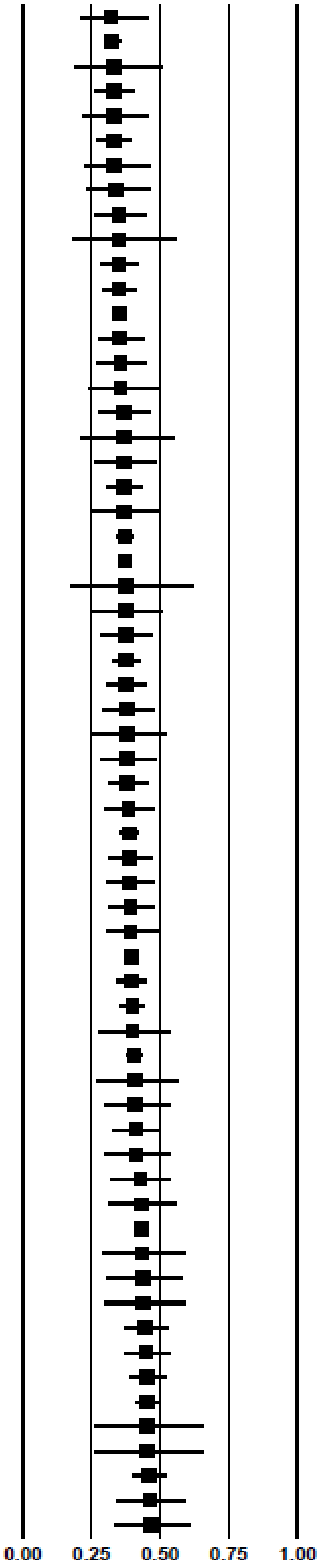

© 2025 [illegible] JAMA Network Open.

Study name	Event rate	Lower limit	Upper limit
Feingold 2018	0.472	0.430	0.514
Schwab 2022	0.482	0.423	0.541
Åkerblom 2020	0.491	0.414	0.568
deHeer 2017	0.500	0.442	0.558
Mahendru 2021	0.500	0.403	0.597
Pasin 2023	0.500	0.392	0.608
Wolfe 2013	0.500	0.367	0.633
Voute 2023	0.502	0.448	0.556
Hållstam 2017	0.509	0.454	0.564
Reiter 2017	0.510	0.428	0.591
Costa 2023	0.515	0.419	0.609
Kallusky 2023	0.516	0.345	0.683
Clark 2018	0.524	0.318	0.721
Bryant 2016	0.531	0.457	0.604
vanEeden 2023	0.533	0.358	0.700
Luciano 2014	0.539	0.479	0.598
León-Suárez 2023	0.551	0.476	0.624
Greenberg 2022	0.560	0.504	0.615
AlvesRodrigues 2022	0.565	0.421	0.700
Sharma 2022	0.566	0.479	0.649
Bapir 2023	0.567	0.539	0.594
Yamada 2022	0.574	0.479	0.664
Rice 2016	0.575	0.499	0.648
Offenbaecher 2013	0.580	0.497	0.659
Taylor 2021	0.580	0.548	0.611
Carleton 2022	0.581	0.559	0.602
Kazemi 2013_B	0.583	0.383	0.759
vandenBerk-Clark 2017	0.585	0.431	0.724
Shebeshi 2023	0.594	0.590	0.597
Giummarra 2020	0.594	0.589	0.599
Dumolard 2023	0.600	0.540	0.657
Andersen 2016	0.600	0.512	0.682
Moraes 2021	0.617	0.507	0.716
Ojeda 2018_C	0.627	0.488	0.748
Marino 2021	0.632	0.452	0.781
Bayram 2014	0.633	0.451	0.784
Barbosa 2016	0.634	0.496	0.753
MurMartí 2017	0.638	0.575	0.697
Sener 2013	0.641	0.481	0.775
Botros 2022	0.648	0.598	0.695
Karas 2020	0.656	0.533	0.762
Siqueira-Campos 2019	0.660	0.562	0.746
DaLuz 2018	0.660	0.562	0.746
Hansdorfer-Korzon 2016	0.661	0.525	0.775
Luque-Reca 2021	0.674	0.611	0.731
Gelonch 2018	0.680	0.587	0.760
Finn 2018	0.703	0.645	0.754
Cappe 2021	0.730	0.617	0.819
Akui 2022	0.750	0.522	0.892
Rapariz-González 2014	0.751	0.708	0.790
Silva 2021	0.760	0.670	0.832
Aloush 2021	0.769	0.710	0.819
Rogal 2015	0.770	0.638	0.864
KibuneNagasako 2016	0.777	0.680	0.851
Siqueira-Campos 2022	0.797	0.717	0.859
DeRoa 2018	0.818	0.676	0.906
Bravo 2019	0.821	0.669	0.912
Boukhira 2021	0.832	0.787	0.869
Upadhyaya 2023_B	0.850	0.738	0.919
Cho 2017	0.879	0.799	0.930
Campos 2021	0.896	0.825	0.940
Pooled estimate	0.402	0.402	0.402
Prediction interval	0.402	0.402	0.402

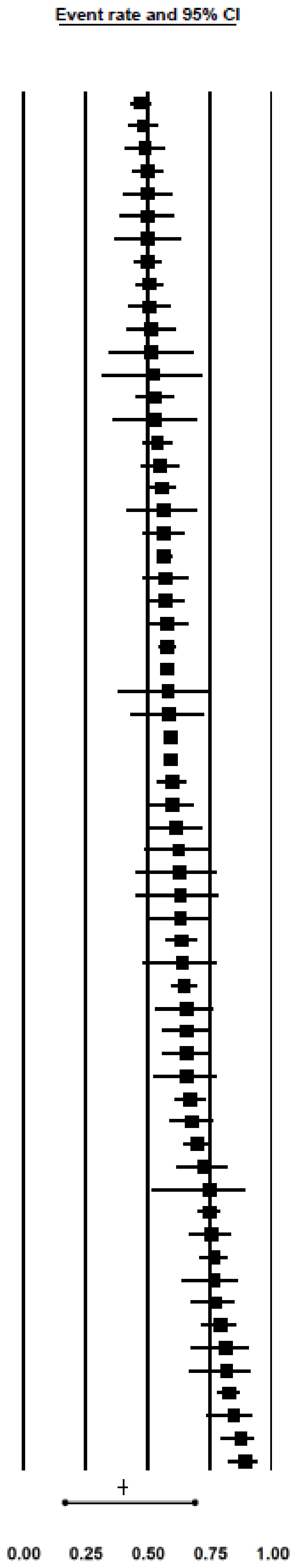

A Network Open.

eTable 3. Pooled Prevalence of Clinical Symptoms of Depression

Study ID	Event Rate	95% CI	Weight
Aşçıbaşı 2022	0.55	(0.42, 0.67)	0.28
Aagaard 2023	0.71	(0.54, 0.84)	0.25
Abrams 2013	0.52	(0.46, 0.58)	0.30
Ahmad 2023	0.70	(0.60, 0.78)	0.29
Ajo 2017	0.32	(0.24, 0.41)	0.29
Åkerblom 2020	0.47	(0.39, 0.55)	0.30
Akui 2022	0.50	(0.29, 0.71)	0.23
Alamam 2019	0.26	(0.19, 0.35)	0.29
Alberts 2020	0.49	(0.37, 0.61)	0.28
Alhalal 2021	0.36	(0.30, 0.42)	0.30
Allaire 2018	0.33	(0.27, 0.39)	0.30
Al-Maharbi 2018	0.71	(0.64, 0.77)	0.30
Aloush 2021	0.80	(0.74, 0.85)	0.30
AlShukaili 2022	0.26	(0.21, 0.31)	0.30
AlvesRodrigues 2022	0.28	(0.17, 0.43)	0.26
Amatsu 2022	0.47	(0.36, 0.59)	0.28
Amital 2014	0.10	(0.03, 0.30)	0.17
Ammitzbøll 2021	0.14	(0.10, 0.19)	0.29
Andersen 2016	0.50	(0.41, 0.58)	0.29
Antaky 2017	0.25	(0.20, 0.31)	0.30
Antunes 2013	0.32	(0.26, 0.39)	0.30
Ardigo 2016	0.15	(0.08, 0.27)	0.25
Areias 2023	0.23	(0.20, 0.27)	0.30
Argoff 2016	0.26	(0.24, 0.27)	0.31
Asada 2022	0.16	(0.13, 0.20)	0.30
Asada 2022_B	0.20	(0.15, 0.26)	0.29
Asih 2014	0.39	(0.34, 0.45)	0.30
Asseyer 2021	0.59	(0.38, 0.77)	0.24
Ataoglu 2013	0.30	(0.22, 0.39)	0.29
Avluk 2014	0.52	(0.38, 0.66)	0.27
Bagnato 2014	0.37	(0.28, 0.47)	0.29
Bailey 2020	0.21	(0.20, 0.22)	0.31
Barbosa 2016	0.35	(0.23, 0.48)	0.27
Barjandi 2021	0.42	(0.34, 0.49)	0.30
Barjandi 2021_B	0.78	(0.67, 0.86)	0.28
Barth 2014	0.55	(0.49, 0.60)	0.30
Barton 2021	0.21	(0.16, 0.27)	0.29
Bayrak 2020	0.08	(0.06, 0.12)	0.29
Bayram 2014	0.53	(0.36, 0.70)	0.25
Bayram 2014_B	0.37	(0.22, 0.55)	0.25
Bell 2016	0.29	(0.28, 0.30)	0.31
Bilen 2022	0.06	(0.03, 0.12)	0.24
Birch 2022	0.19	(0.06, 0.45)	0.19
Bohn 2013	0.73	(0.65, 0.81)	0.29
Botros 2022	0.80	(0.76, 0.84)	0.30
Boukhira 2021	0.80	(0.76, 0.84)	0.30
Bravo 2019	0.51	(0.36, 0.66)	0.27
BromleyMilton 2013	0.27	(0.26, 0.28)	0.31
Bruce 2021	0.79	(0.76, 0.82)	0.30
Brunner 2023	0.47	(0.40, 0.53)	0.30
Bryant 2016	0.27	(0.21, 0.34)	0.30
Calandre 2022	0.85	(0.81, 0.89)	0.30
Calderón 2016	0.38	(0.26, 0.51)	0.28

Study ID	Event Rate	95% CI	Weight
Calpin 2017	0.21	(0.14, 0.30)	0.28
Campbell 2015b	0.47	(0.44, 0.49)	0.31
Campos 2021	0.83	(0.75, 0.88)	0.28
Cappe 2021	0.53	(0.42, 0.64)	0.28
Carleton 2022	0.58	(0.56, 0.60)	0.31
Carrillo-de-la-Peña 2015	0.56	(0.42, 0.69)	0.27
Carrillo-Izquierdo 2018	0.81	(0.75, 0.85)	0.30
CasteloAzevedo 2021	0.23	(0.20, 0.27)	0.30
Chaabouni 2022	0.10	(0.04, 0.22)	0.23
Chahal-Kummen 2019	0.12	(0.06, 0.23)	0.25
Chahal-Kummen 2023	0.16	(0.10, 0.24)	0.28
Challa 2017	0.11	(0.06, 0.18)	0.27
Chana 2021	0.32	(0.19, 0.49)	0.26
Chang 2018	0.54	(0.45, 0.62)	0.29
Chen 2022	0.17	(0.13, 0.21)	0.30
Cheng 2018	0.22	(0.19, 0.25)	0.30
Cheng 2022	0.18	(0.15, 0.20)	0.31
Cho 2017	0.82	(0.73, 0.88)	0.28
Choi 2018	0.15	(0.09, 0.23)	0.28
Chytas 2023	0.59	(0.50, 0.66)	0.30
Civelek 2022	0.23	(0.14, 0.34)	0.27
Cody 2019	0.46	(0.42, 0.50)	0.30
Conway 2020	0.43	(0.37, 0.49)	0.30
Corriger 2022	0.38	(0.32, 0.44)	0.30
Costa 2023	0.49	(0.39, 0.58)	0.29
DaLuz 2018	0.63	(0.53, 0.72)	0.29
Damci 2022	0.23	(0.21, 0.25)	0.31
Davis 2014	0.13	(0.05, 0.28)	0.23
Davis 2014_B	0.37	(0.27, 0.49)	0.28
Dear 2023	0.60	(0.57, 0.62)	0.31
deHeer 2017	0.42	(0.36, 0.48)	0.30
DeLaRosa 2023	0.19	(0.18, 0.20)	0.31
DelPozo-Cruz 2017	0.62	(0.52, 0.71)	0.29
deMoraesVieira 2014	0.33	(0.27, 0.39)	0.30
Depintor 2016	0.33	(0.27, 0.40)	0.30
DeRoa 2018	0.57	(0.42, 0.71)	0.27
Doherty 2017	0.57	(0.47, 0.66)	0.29
Domenech 2013	0.16	(0.10, 0.25)	0.28
DosSantosProença 2023	0.27	(0.19, 0.36)	0.29
Dougados 2017	0.16	(0.12, 0.22)	0.29
Drummond 2013	0.43	(0.25, 0.63)	0.24
Drummond 2013_B	0.38	(0.18, 0.63)	0.22
D'Souza 2020	0.71	(0.67, 0.74)	0.30
Dubois-Mendes 2021	0.28	(0.19, 0.39)	0.28
Dumolard 2023	0.41	(0.35, 0.47)	0.30

© 2025 Aaron RV et al. *JAMA Network Open.*

Study ID	Event Rate	95% CI	Weight
Ecija 2022	0.26	(0.21, 0.32)	0.30
Edit 2013	0.28	(0.20, 0.37)	0.29
Elrashidi 2018	0.15	(0.13, 0.18)	0.30
Englbrecht 2019	0.21	(0.18, 0.24)	0.31
Eriksen 2021	0.46	(0.29, 0.65)	0.25
Falling 2021	0.14	(0.04, 0.35)	0.19
Farin 2015	0.17	(0.13, 0.22)	0.30
Farzad 2021	0.17	(0.10, 0.27)	0.27
Feingold 2017	0.42	(0.39, 0.45)	0.31
Feingold 2018	0.56	(0.52, 0.60)	0.31
Feingold 2020	0.20	(0.15, 0.26)	0.29
Feliu-Soler 2017	0.84	(0.78, 0.89)	0.29
Fiegl 2019	0.66	(0.62, 0.70)	0.30
Finn 2018	0.62	(0.57, 0.68)	0.30
Flores-Curiel 2023	0.39	(0.30, 0.49)	0.29
Foti 2022	0.24	(0.18, 0.31)	0.29
Foti 2022_B	0.13	(0.08, 0.20)	0.28
Frede 2023	0.25	(0.19, 0.33)	0.29
Freo 2021	0.27	(0.20, 0.34)	0.29
Garaigordobil 2016	0.64	(0.53, 0.73)	0.29
Garbi 2014	0.62	(0.49, 0.73)	0.28
GarciaCarrasco 2013	0.41	(0.29, 0.55)	0.27
Garnæs 2022	0.11	(0.09, 0.14)	0.30
Gavilan-Carrera 2022	0.41	(0.36, 0.46)	0.30
Gebauer 2019	0.56	(0.51, 0.61)	0.30
Gelonch 2017	0.68	(0.58, 0.76)	0.29
Gelonch 2018	0.86	(0.78, 0.91)	0.28
Gerdle 2019	0.35	(0.35, 0.36)	0.31
Gerra 2021	0.79	(0.75, 0.83)	0.30
Gisev 2019	0.44	(0.42, 0.47)	0.31
Giummarra 2020	0.65	(0.65, 0.66)	0.31
Goesling 2015	0.22	(0.20, 0.25)	0.31
Goesling 2015_B	0.59	(0.54, 0.63)	0.31
Goesling 2015b	0.36	(0.34, 0.38)	0.31
Goesling 2018	0.32	(0.25, 0.40)	0.29
Gomes 2023	0.10	(0.08, 0.12)	0.30
Gorpynchenko 2021	0.16	(0.09, 0.26)	0.27
Gota 2017	0.60	(0.54, 0.65)	0.30
Govind 2020	0.30	(0.21, 0.41)	0.28
Greenberg 2022	0.67	(0.62, 0.72)	0.30
Gupta 2014	0.87	(0.59, 0.97)	0.16
Gupta 2016	0.92	(0.83, 0.96)	0.24
Hållstam 2017	0.46	(0.40, 0.51)	0.30
Hamdi 2021	0.23	(0.15, 0.35)	0.27
Hamdi 2021_B	0.63	(0.47, 0.76)	0.26
Han 2019	0.17	(0.14, 0.21)	0.30
Hansdorfer-Korzon 2016	0.28	(0.18, 0.42)	0.27
Hardy 2022	0.18	(0.14, 0.23)	0.30
Harrison 2016	0.56	(0.49, 0.62)	0.30

Study ID	Event Rate	95% CI	Weight
Hasuo 2020	0.47	(0.39, 0.55)	0.30
Häuser 2014	0.73	(0.66, 0.79)	0.29
Häuser 2015b	0.46	(0.38, 0.55)	0.30
Hoban 2015	0.44	(0.41, 0.47)	0.31
Hochman 2013	0.14	(0.06, 0.29)	0.23
Hogestol 2017	0.07	(0.02, 0.19)	0.20
Holloway 2017	0.40	(0.36, 0.45)	0.30
Honda 2022	0.49	(0.35, 0.63)	0.27
Hong 2018	0.55	(0.47, 0.62)	0.30
Hughes 2019	0.46	(0.41, 0.52)	0.30
Iannuccelli 2021	0.50	(0.39, 0.61)	0.28
Iannuccelli 2021_B	0.17	(0.10, 0.27)	0.27
Inanc 2014	0.35	(0.24, 0.48)	0.27
Ingegnoli 2022	0.49	(0.40, 0.57)	0.29
Islam 2023	0.36	(0.28, 0.44)	0.29
Islam 2023_B	0.35	(0.24, 0.48)	0.28
Jaiswal 2016	0.36	(0.25, 0.49)	0.28
Jaiswal 2016_B	0.34	(0.24, 0.47)	0.28
Janela 2022	0.27	(0.22, 0.32)	0.30
Järemo 2022	0.32	(0.25, 0.40)	0.29
Jedel 2020	0.25	(0.15, 0.38)	0.27
Jegan 2017	0.22	(0.18, 0.26)	0.30
Jensen 2016	0.29	(0.26, 0.32)	0.31
Jiao 2021	0.53	(0.45, 0.62)	0.29
Jimenez-Rodríguez 2014	0.91	(0.78, 0.97)	0.21
Jimenez-Rodríguez 2014_B	0.41	(0.26, 0.58)	0.26
Jin 2021	0.13	(0.12, 0.15)	0.31
Jin 2021_B	0.16	(0.14, 0.17)	0.31
Jindal 2021	0.34	(0.26, 0.44)	0.29
John 2022	0.26	(0.23, 0.30)	0.30
John 2022_B	0.70	(0.62, 0.77)	0.29
John 2022_C	0.27	(0.20, 0.36)	0.29
Jouini 2014	0.24	(0.20, 0.28)	0.30
Joyce 2021	0.35	(0.30, 0.41)	0.30
Kadimpati 2015	0.69	(0.65, 0.73)	0.30
Kallusky 2023	0.19	(0.09, 0.37)	0.23
Karafin 2018	0.36	(0.25, 0.48)	0.28
Karas 2020	0.72	(0.60, 0.82)	0.28
Kazemi 2013	0.38	(0.18, 0.62)	0.22
Kazemi 2013_B	0.50	(0.31, 0.69)	0.24
Kec 2022	0.36	(0.31, 0.41)	0.30
Khazen 2021	0.25	(0.16, 0.36)	0.27
KibuneNagasako 2016	0.58	(0.47, 0.67)	0.29
Kim 2021	0.48	(0.36, 0.60)	0.28
Koh 2014	0.11	(0.06, 0.20)	0.26
Kosson 2018	0.18	(0.16, 0.20)	0.31
Lamerato 2023	0.43	(0.37, 0.50)	0.30
Landefeld 2017	0.62	(0.54, 0.69)	0.30
Larance 2019	0.44	(0.41, 0.47)	0.31
Laurent 2014	0.42	(0.32, 0.52)	0.29
Laurent 2022	0.22	(0.13, 0.36)	0.26
Lee 2020	0.43	(0.30, 0.57)	0.27

© 2025 Aaron RV et al. *JAMA Network Open.*

Study ID	Event Rate	95% CI	Weight
León-Suárez 2023	0.33	(0.26, 0.40)	0.30
Lerman 2015	0.66	(0.61, 0.70)	0.30
Lin 2017	0.34	(0.28, 0.41)	0.30
Lindemann 2023	0.44	(0.35, 0.54)	0.29
Liu 2018	0.21	(0.19, 0.22)	0.31
Longo 2022	0.61	(0.50, 0.70)	0.29
Lopez 2013	0.57	(0.52, 0.62)	0.30
Luciano 2014	0.25	(0.20, 0.31)	0.30
Luque-Reca 2021	0.40	(0.34, 0.46)	0.30
Mahendru 2021	0.54	(0.44, 0.63)	0.29
Mahlich 2019	0.11	(0.09, 0.14)	0.30
Mann 2017	0.20	(0.17, 0.23)	0.30
Mantyselka 2017	0.26	(0.17, 0.37)	0.28
Marcum 2014	0.32	(0.25, 0.39)	0.30
Marino 2021	0.37	(0.22, 0.55)	0.25
Mattila-Rautiainen 2023	0.32	(0.16, 0.53)	0.23
McCracken 2023	0.43	(0.39, 0.47)	0.31
McWilliams 2017	0.77	(0.71, 0.82)	0.30
Merlin 2015	0.30	(0.16, 0.48)	0.25
Midbari 2016	0.61	(0.44, 0.75)	0.26
Miller-Matero 2016	0.26	(0.18, 0.35)	0.29
Moraes 2021	0.63	(0.52, 0.73)	0.28
Morlà 2021_B	0.43	(0.31, 0.55)	0.28
Morlà 2021	0.32	(0.24, 0.42)	0.29
Murata 2019	0.19	(0.16, 0.23)	0.30
MurMartí 2017	0.53	(0.47, 0.59)	0.30
Mustonen 2019	0.45	(0.37, 0.53)	0.29
Neblett 2016	0.79	(0.76, 0.82)	0.31
Nguyen 2021	0.57	(0.53, 0.60)	0.31
Norman-Nott 2022	0.33	(0.04, 0.85)	0.09
Offenbaecher 2013	0.44	(0.36, 0.52)	0.30
Ojeda 2018	0.28	(0.20, 0.37)	0.29
Ojeda 2018_B	0.25	(0.18, 0.35)	0.28
Ojeda 2018_C	0.39	(0.27, 0.53)	0.27
Peilot 2018	0.45	(0.31, 0.60)	0.27
Penacoba 2023	0.19	(0.15, 0.24)	0.30
Phillips 2014	0.21	(0.14, 0.31)	0.28
PicchiantiDiamanti 2020	0.42	(0.33, 0.52)	0.29
Pinto 2022	0.24	(0.16, 0.34)	0.28
Plinsinga 2020	0.03	(0.01, 0.16)	0.14
Plinsinga 2023	0.23	(0.20, 0.26)	0.30
Porru 2023	0.19	(0.10, 0.34)	0.25
Porru 2023_B	0.07	(0.02, 0.25)	0.17
Poulin 2016	0.57	(0.44, 0.69)	0.28
Prateepavanich 2018	0.13	(0.07, 0.23)	0.26
Preti 2019	0.82	(0.69, 0.90)	0.26

Study ID	Event Rate	95% CI	Weight
Preti 2019_B	0.49	(0.39, 0.60)	0.29
Preti 2019_C	0.49	(0.39, 0.59)	0.29
Priol 2023	0.20	(0.12, 0.31)	0.27
Puto 2023	0.64	(0.57, 0.71)	0.30
Quidé 2022	0.12	(0.04, 0.31)	0.20
Quinlan 2021	0.82	(0.70, 0.90)	0.26
Racine 2017	0.50	(0.46, 0.54)	0.31
Rayner 2016	0.55	(0.52, 0.58)	0.31
Reiter 2017	0.54	(0.46, 0.62)	0.30
Reiter 2017_B	0.18	(0.13, 0.25)	0.29
Reiter 2018	0.20	(0.15, 0.27)	0.29
Rexelius 2020	0.26	(0.15, 0.41)	0.26
Rezaei 2014	0.66	(0.56, 0.75)	0.29
Rice 2016	0.56	(0.49, 0.64)	0.30
Rice 2016_B	0.12	(0.08, 0.18)	0.28
Robinson 2013	0.46	(0.36, 0.56)	0.29
Rogal 2015	0.66	(0.52, 0.77)	0.27
Rogers 2021	0.06	(0.04, 0.11)	0.28
Rojas 2021	0.19	(0.08, 0.38)	0.22
Rometsch 2022	0.50	(0.35, 0.65)	0.27
Rometsch 2023	0.59	(0.49, 0.68)	0.29
Rouch 2021	0.16	(0.12, 0.21)	0.29
Rovner 2017	0.31	(0.29, 0.34)	0.31
Roy 2022	0.35	(0.30, 0.40)	0.30
RusMakovec 2015	0.33	(0.24, 0.44)	0.28
Rusu 2016	0.26	(0.17, 0.36)	0.28
Saariaho 2015	0.31	(0.26, 0.37)	0.30
Sachau 2023	0.25	(0.16, 0.37)	0.27
Sagheer 2013	0.12	(0.08, 0.19)	0.28
Saglam 2022	0.41	(0.28, 0.56)	0.27
Scherrer 2015	0.58	(0.53, 0.63)	0.30
Schmukler 2023	0.26	(0.22, 0.31)	0.30
Schmukler 2023_B	0.37	(0.28, 0.46)	0.29
Schmukler 2023_C	0.32	(0.22, 0.44)	0.28
Schwab 2022	0.47	(0.41, 0.53)	0.30
Schwarm 2021	0.54	(0.38, 0.69)	0.27
Seekatz 2016	0.38	(0.33, 0.43)	0.30
Sener 2013	0.36	(0.23, 0.52)	0.26
Sengupta 2023	0.74	(0.66, 0.80)	0.29
Shamji 2016	0.63	(0.55, 0.70)	0.30
Sharma 2022	0.82	(0.75, 0.88)	0.28
Shmagel 2016	0.22	(0.19, 0.25)	0.30
Si 2019	0.17	(0.14, 0.20)	0.30
Silva 2021	0.54	(0.44, 0.63)	0.29
Siqueira-Campos 2019	0.63	(0.53, 0.72)	0.29
Siqueira-Campos 2022	0.73	(0.65, 0.80)	0.29
Sitges 2018	0.51	(0.35, 0.67)	0.26
Slawek 2021	0.46	(0.39, 0.53)	0.30
Song 2015	0.22	(0.16, 0.29)	0.29
Soriano-Maldonado 2015	0.70	(0.66, 0.74)	0.30
Souza 2021	0.97	(0.80, 1.00)	0.12

© 2025 Aaron RV et al. *JAMA Network Open.*

Study ID	Event Rate	95% CI	Weight
Sparkes 2015	0.27	(0.17, 0.40)	0.27
Stefani 2019	0.81	(0.71, 0.88)	0.28
Stefani 2019_B	0.61	(0.45, 0.75)	0.26
Stefani 2019_C	0.32	(0.25, 0.41)	0.29
Stehlik 2018	0.19	(0.08, 0.36)	0.23
Stein 2015	0.28	(0.21, 0.35)	0.29
Steiner 2017	0.97	(0.93, 0.98)	0.25
Stokholm 2022	0.30	(0.19, 0.44)	0.27
SundaraRajan 2017	0.19	(0.11, 0.32)	0.26
Sundstrom 2023	0.71	(0.66, 0.75)	0.30
Tanaka 2022	0.35	(0.26, 0.45)	0.29
Tardif 2023	0.42	(0.41, 0.43)	0.31
Tatebe 2016	0.50	(0.35, 0.65)	0.26
Taylor 2021	0.51	(0.47, 0.54)	0.31
Teixido-Abiol 2022	0.48	(0.36, 0.60)	0.28
Teixido-Abiol 2022_B	0.43	(0.32, 0.55)	0.28
Terassi 2020	0.05	(0.03, 0.10)	0.26
Tetsunaga 2013	0.77	(0.69, 0.84)	0.29
Teychenne 2019	0.43	(0.28, 0.58)	0.27
Thakral 2018	0.34	(0.31, 0.36)	0.31
ThiNguy 2022	0.32	(0.16, 0.53)	0.23
Thomas 2022	0.32	(0.23, 0.42)	0.29
Thompson 2023	0.47	(0.43, 0.52)	0.30
Tocchetto 2023	0.58	(0.47, 0.68)	0.29
Tsuji 2016	0.16	(0.13, 0.20)	0.30
Uebelacker 2015	0.68	(0.59, 0.75)	0.29
Uniyal 2017_B	0.55	(0.41, 0.67)	0.28
Upadhyaya 2023	0.32	(0.25, 0.40)	0.29
Upadhyaya 2023_B	0.95	(0.86, 0.98)	0.20
Vance 2018	0.31	(0.24, 0.39)	0.29

Study ID	Event Rate	95% CI	Weight
vandenBerk-Clark 2017	0.24	(0.14, 0.40)	0.26
vanEeden 2023	0.47	(0.30, 0.64)	0.25
VanOvermeire 2022	0.45	(0.35, 0.54)	0.29
VanRyckeghem 2013	0.23	(0.15, 0.34)	0.28
Varinen 2019	0.09	(0.07, 0.12)	0.30
Vilalta-Abella 2015	0.40	(0.21, 0.62)	0.23
Villafaina 2019	0.57	(0.39, 0.74)	0.25
Voute 2023	0.38	(0.33, 0.43)	0.30
Vukojevi? 2022	0.28	(0.18, 0.40)	0.28
Wadley 2019	0.48	(0.35, 0.62)	0.27
Wadley 2020	0.11	(0.05, 0.21)	0.25
Wahlman 2014	0.34	(0.28, 0.40)	0.30
Weingarten 2016	0.40	(0.27, 0.54)	0.27
Whitlock 2017	0.50	(0.47, 0.53)	0.31
Widenka 2021	0.10	(0.05, 0.21)	0.24
Wildes 2020	0.33	(0.26, 0.41)	0.29
Wilson 2015	0.37	(0.28, 0.47)	0.29
Wilson 2022	0.27	(0.19, 0.37)	0.28
Wolfe 2013	0.64	(0.50, 0.75)	0.27
Wong 2017	0.50	(0.42, 0.58)	0.29
Wright 2017	0.62	(0.40, 0.80)	0.23
Yadav 2023	0.36	(0.30, 0.42)	0.30
Yamada 2022	0.31	(0.23, 0.41)	0.29
Yin 2023	0.51	(0.40, 0.62)	0.29
Yu 2021	0.80	(0.76, 0.83)	0.30
Zaidel 2021	0.08	(0.08, 0.09)	0.31
Zakrzewska 2017	0.31	(0.25, 0.37)	0.30
Zambelli 2021	0.36	(0.33, 0.39)	0.31
Zubatsky 2020	0.58	(0.52, 0.63)	0.30
**Overall**	0.39	(0.37, 0.41)	

© 2025 Aaron RV et al. *JAMA Network Open.*

eTable 4. Pooled Prevalence of Clinical Symptoms of Anxiety

Study ID	Event Rate	95% CI	Weight
Penacoba 2023	0.32	(0.27, 0.38)	0.59
Campos 2021	0.90	(0.83, 0.94)	0.50
DeRoa 2018	0.82	(0.68, 0.91)	0.44
Sener 2013	0.64	(0.48, 0.77)	0.48
Vance 2018	0.39	(0.31, 0.47)	0.58
Barjandi 2021	0.38	(0.31, 0.46)	0.58
Karas 2020	0.66	(0.53, 0.76)	0.52
Luque-Reca 2021	0.67	(0.61, 0.73)	0.59
Barbosa 2016	0.63	(0.50, 0.75)	0.51
Cappe 2021	0.73	(0.62, 0.82)	0.52
Siqueira-Campos 2019	0.66	(0.56, 0.75)	0.55
Thomas 2022	0.35	(0.26, 0.45)	0.55
Mustonen 2019	0.25	(0.18, 0.33)	0.56
Schwab 2022	0.48	(0.42, 0.54)	0.60
Bayram 2014	0.63	(0.45, 0.78)	0.45
Bayram 2014_B	0.37	(0.22, 0.55)	0.45
Bryant 2016	0.53	(0.46, 0.60)	0.59
DaLuz 2018	0.66	(0.56, 0.75)	0.55
Hansdorfer-Korzon 2016	0.66	(0.52, 0.77)	0.51
Weingarten 2016	0.44	(0.31, 0.58)	0.51
Gelonch 2018	0.68	(0.59, 0.76)	0.56
vanEeden 2023	0.53	(0.36, 0.70)	0.45
Miller-Matero 2016	0.39	(0.30, 0.48)	0.56
Barjandi 2021_B	0.27	(0.18, 0.37)	0.53
Ecija 2022	0.37	(0.31, 0.43)	0.59
Stehlik 2018	0.33	(0.19, 0.51)	0.44
Jedel 2020	0.46	(0.34, 0.59)	0.52
Marino 2021	0.63	(0.45, 0.78)	0.45
Allaire 2018	0.24	(0.19, 0.29)	0.59
Siqueira-Campos 2022	0.80	(0.72, 0.86)	0.55
MurMartí 2017	0.64	(0.57, 0.70)	0.59
Bravo 2019	0.82	(0.67, 0.91)	0.42
Ojeda 2018_C	0.63	(0.49, 0.75)	0.51
Iannuccelli 2021	0.37	(0.26, 0.48)	0.53
Häuser 2015b	0.45	(0.37, 0.53)	0.58
ThiNguy 2022	0.46	(0.27, 0.66)	0.41
Luciano 2014	0.54	(0.48, 0.60)	0.60
deHeer 2017	0.50	(0.44, 0.56)	0.60
Offenbaecher 2013	0.58	(0.50, 0.66)	0.58
Plinsinga 2020	0.25	(0.14, 0.41)	0.46
Taylor 2021	0.58	(0.55, 0.61)	0.62
McCracken 2023	0.26	(0.23, 0.30)	0.61
Argoff 2016	0.37	(0.35, 0.39)	0.62
Drummond 2013	0.35	(0.19, 0.56)	0.41
Rapariz-González 2014	0.75	(0.71, 0.79)	0.60

Study ID	Event Rate	95% CI	Weight
Dubois-Mendes 2021	0.43	(0.32, 0.54)	0.54
Järemo 2022	0.33	(0.26, 0.41)	0.57
Dumolard 2023	0.60	(0.54, 0.66)	0.60
Aloush 2021	0.77	(0.71, 0.82)	0.58
D'Souza 2020	0.45	(0.41, 0.49)	0.61
Upadhyaya 2023_B	0.85	(0.74, 0.92)	0.46
Aşçıbaşı 2022	0.34	(0.23, 0.46)	0.52
Mahendru 2021	0.50	(0.40, 0.60)	0.56
Chana 2021	0.22	(0.11, 0.38)	0.43
Greenberg 2022	0.56	(0.50, 0.61)	0.60
Åkerblom 2020	0.49	(0.41, 0.57)	0.58
Cho 2017	0.88	(0.80, 0.93)	0.49
Iannuccelli 2021_B	0.15	(0.08, 0.24)	0.49
Pasin 2023	0.50	(0.39, 0.61)	0.55
KibuneNagasako 2016	0.78	(0.68, 0.85)	0.53
Domenech 2013	0.30	(0.22, 0.40)	0.55
DosSantosProença 2023	0.36	(0.27, 0.45)	0.56
Hogestol 2017	0.06	(0.02, 0.18)	0.31
Gentili 2019	0.25	(0.20, 0.31)	0.59
Chahal-Kummen 2023	0.24	(0.17, 0.33)	0.55
Hasuo 2020	0.45	(0.37, 0.53)	0.58
Finn 2018	0.70	(0.65, 0.75)	0.59
Reiter 2017	0.51	(0.43, 0.59)	0.58
Andersen 2016	0.60	(0.51, 0.68)	0.57
Rometsch 2023	0.37	(0.28, 0.47)	0.55
Botros 2022	0.65	(0.60, 0.69)	0.60
Peilot 2018	0.44	(0.30, 0.59)	0.49
Hamdi 2021	0.16	(0.09, 0.27)	0.48
Islam 2023	0.41	(0.33, 0.50)	0.57
Dear 2023	0.41	(0.38, 0.43)	0.62
Clark 2019	0.26	(0.20, 0.33)	0.57
Rice 2016_B	0.15	(0.10, 0.21)	0.55
Clark 2018	0.52	(0.32, 0.72)	0.41
Corriger 2022	0.46	(0.40, 0.52)	0.60
Voute 2023	0.50	(0.45, 0.56)	0.60
Reiter 2018	0.13	(0.09, 0.19)	0.54
Birch 2022	0.38	(0.18, 0.62)	0.36
Kallusky 2023	0.52	(0.35, 0.68)	0.46
Upadhyaya 2023	0.17	(0.12, 0.24)	0.55
Chahal-Kummen 2019	0.33	(0.22, 0.46)	0.51
Ardigo 2016	0.32	(0.21, 0.46)	0.50
Reiter 2017_B	0.10	(0.06, 0.16)	0.52
Costa 2023	0.51	(0.42, 0.61)	0.56
Priol 2023	0.31	(0.21, 0.43)	0.52
Gerdle 2019	0.40	(0.39, 0.40)	0.62
PicchiantiDiamanti 2020	0.38	(0.29, 0.48)	0.56

© 2025 Aaron RV et al. *JAMA Network Open.*

Study ID	Event Rate	95% CI	Weight
RusMakovec 2015	0.20	(0.12, 0.30)	0.51
Cheng 2022	0.25	(0.23, 0.28)	0.62
Hamdi 2021_B	0.20	(0.10, 0.35)	0.44
Teixido-Abiol 2022	0.41	(0.30, 0.53)	0.53
Teixido-Abiol 2022_B	0.31	(0.21, 0.43)	0.52
Damci 2022	0.27	(0.24, 0.29)	0.62
BromleyMilton 2013	0.31	(0.29, 0.32)	0.62
Depintor 2016	0.38	(0.31, 0.45)	0.58
Wildes 2020	0.19	(0.14, 0.26)	0.56
Farzad 2021	0.32	(0.23, 0.43)	0.54
Garnæs 2022	0.28	(0.24, 0.32)	0.61
Rovner 2017	0.35	(0.33, 0.38)	0.62
Plinsinga 2023	0.21	(0.18, 0.24)	0.61
Jouini 2014	0.40	(0.36, 0.44)	0.61
Chen 2022	0.17	(0.13, 0.22)	0.58
Karafin 2018	0.29	(0.19, 0.41)	0.52
Larance 2019	0.21	(0.19, 0.24)	0.61
Elrashidi 2018	0.14	(0.12, 0.17)	0.61
Poulin 2016	0.43	(0.31, 0.56)	0.52
Hardy 2022	0.04	(0.02, 0.07)	0.49
Rice 2016	0.58	(0.50, 0.65)	0.58
Jensen 2016	0.39	(0.36, 0.42)	0.61
Joyce 2021	0.30	(0.26, 0.36)	0.60
Hållstam 2017	0.51	(0.45, 0.56)	0.60
Kosson 2018	0.32	(0.30, 0.35)	0.62
Inanc 2014	0.24	(0.15, 0.37)	0.49
Zakrzewska 2017	0.45	(0.39, 0.52)	0.59
Lee 2020	0.31	(0.20, 0.45)	0.50
Alamam 2019	0.39	(0.31, 0.48)	0.57
Drummond 2013_B	0.19	(0.06, 0.45)	0.29
Farin 2015	0.26	(0.21, 0.32)	0.59
vandenBerk-Clark 2017	0.59	(0.43, 0.72)	0.49
Wolfe 2013	0.50	(0.37, 0.63)	0.51
Calderón 2016	0.38	(0.26, 0.51)	0.51
Laurent 2022	0.47	(0.34, 0.61)	0.51
Pinto 2022	0.24	(0.16, 0.34)	0.52
Ojeda 2018_B	0.39	(0.30, 0.49)	0.56
Shebeshi 2023	0.59	(0.59, 0.60)	0.62
Laurent 2014	0.38	(0.28, 0.49)	0.55
Giummarra 2020	0.59	(0.59, 0.60)	0.62
Gisev 2019	0.21	(0.19, 0.24)	0.61
Shamji 2016	0.23	(0.17, 0.30)	0.56
AlvesRodrigues 2022	0.57	(0.42, 0.70)	0.50
Hoban 2015	0.22	(0.19, 0.25)	0.61
Yamada 2022	0.57	(0.48, 0.66)	0.56

Study ID	Event Rate	95% CI	Weight
Lindemann 2023	0.16	(0.10, 0.24)	0.52
Phillips 2014	0.31	(0.22, 0.40)	0.55
Tardif 2023	0.43	(0.43, 0.44)	0.62
Campbell 2015b	0.23	(0.21, 0.25)	0.62
Stokholm 2022	0.38	(0.26, 0.52)	0.51
Sparkes 2015	0.36	(0.24, 0.49)	0.51
Slawek 2021	0.35	(0.28, 0.42)	0.58
Falling 2021	0.45	(0.26, 0.66)	0.41
Chang 2018	0.35	(0.28, 0.44)	0.57
Calpin 2017	0.29	(0.21, 0.39)	0.55
Alberts 2020	0.42	(0.30, 0.54)	0.53
Rometsch 2022	0.41	(0.27, 0.56)	0.49
Ojeda 2018	0.38	(0.29, 0.47)	0.56
Janela 2022	0.29	(0.24, 0.35)	0.60
Areias 2023	0.26	(0.22, 0.30)	0.61
Rogal 2015	0.77	(0.64, 0.86)	0.48
Yadav 2023	0.35	(0.29, 0.41)	0.59
Moraes 2021	0.62	(0.51, 0.72)	0.54
Bailey 2020	0.28	(0.27, 0.29)	0.62
Bayrak 2020	0.40	(0.34, 0.45)	0.60
Feingold 2018	0.47	(0.43, 0.51)	0.61
SundaraRajan 2017	0.28	(0.18, 0.41)	0.50
Bilen 2022	0.11	(0.06, 0.18)	0.50
Sagheer 2013	0.16	(0.11, 0.23)	0.54
Schwarm 2021	0.44	(0.29, 0.59)	0.48
Bapir 2023	0.57	(0.54, 0.59)	0.62
Vukojevi? 2022	0.25	(0.16, 0.37)	0.51
Kec 2022	0.38	(0.33, 0.43)	0.60
Islam 2023_B	0.37	(0.25, 0.50)	0.52
Feingold 2017	0.37	(0.34, 0.40)	0.61
Sachau 2023	0.33	(0.23, 0.46)	0.52
Uniyal 2017_B	0.40	(0.28, 0.53)	0.52
Tanaka 2022	0.25	(0.17, 0.35)	0.54
Carleton 2022	0.58	(0.56, 0.60)	0.62
Freo 2021	0.10	(0.06, 0.16)	0.52
Boukhira 2021	0.83	(0.79, 0.87)	0.59
Akui 2022	0.75	(0.52, 0.89)	0.36
Feingold 2020	0.22	(0.17, 0.29)	0.58
Kim 2021	0.21	(0.13, 0.33)	0.50
Conway 2020	0.33	(0.27, 0.39)	0.59
Ajo 2017	0.39	(0.31, 0.48)	0.57
Gorpynchenko 2021	0.20	(0.13, 0.31)	0.51
Kazemi 2013_B	0.58	(0.38, 0.76)	0.42
Chaabouni 2022	0.02	(0.00, 0.13)	0.16
Kazemi 2013	0.25	(0.10, 0.51)	0.32
Sharma 2022	0.57	(0.48, 0.65)	0.57
Silva 2021	0.76	(0.67, 0.83)	0.55
León-Suárez 2023	0.55	(0.48, 0.62)	0.58
DeLaRosa 2023	0.15	(0.14, 0.16)	0.62
**Overall**	0.40	(0.38, 0.42)	

© 2025 Aaron RV et al. *JAMA Network Open.*

eTable 5. Pooled Prevalence of Depressive Disorders

Study ID	Event Rate	95% CI	Weight
**Major Depressive Disorder**			
Aagaard 2023	0.50	(0.34, 0.66)	2.32
Alciati 2018	0.05	(0.02, 0.12)	2.11
Annagür 2014	0.49	(0.40, 0.58)	2.47
Asih 2016	0.63	(0.59, 0.67)	2.53
Blakey 2018	0.25	(0.22, 0.29)	2.53
Bucourt 2021	0.48	(0.34, 0.62)	2.38
Bucourt 2021_B	0.21	(0.12, 0.35)	2.31
Campbell 2015	0.11	(0.10, 0.12)	2.54
Caumo 2022	0.69	(0.54, 0.80)	2.36
Ciaramella 2015	0.33	(0.28, 0.37)	2.52
Ciaramella 2015_B	0.28	(0.24, 0.32)	2.52
Durán 2021	0.28	(0.25, 0.31)	2.53
Emery 2014	0.55	(0.42, 0.67)	2.41
Fiegl 2019	0.61	(0.56, 0.65)	2.53
Filippon 2013	0.57	(0.48, 0.66)	2.47
Fischer-Jbali 2022	0.31	(0.16, 0.51)	2.22
Gündüz 2018	0.46	(0.33, 0.60)	2.40
Gündüz 2019	0.14	(0.07, 0.25)	2.31
Gündüz 2019b	0.43	(0.31, 0.55)	2.43
Hudak 2022	0.72	(0.64, 0.78)	2.48
Jain 2013	0.41	(0.32, 0.51)	2.46
Kha 2020	0.26	(0.19, 0.36)	2.44
Knaster 2016	0.20	(0.13, 0.29)	2.42
Kroenke 2020	0.44	(0.37, 0.52)	2.49
Kroenke 2020_B	0.15	(0.11, 0.20)	2.48
Lee 2014	0.49	(0.34, 0.64)	2.35
Mayer 2013	0.50	(0.44, 0.55)	2.52
Mayer 2013_B	0.55	(0.52, 0.57)	2.54
Mehraban 2014	0.62	(0.51, 0.71)	2.45
Muharam 2022	0.28	(0.22, 0.36)	2.48
Nacak 2021	0.56	(0.43, 0.67)	2.42
Neikrug 2017	0.35	(0.29, 0.40)	2.51
Osório 2016	0.28	(0.17, 0.42)	2.36
Parisi 2022	0.72	(0.66, 0.77)	2.50
Proctor 2013	0.44	(0.38, 0.51)	2.51
Radat 2013	0.17	(0.12, 0.23)	2.46
Rouch 2023	0.09	(0.08, 0.10)	2.53
Rusu 2016	0.35	(0.26, 0.47)	2.43
Sohn 2016	0.59	(0.42, 0.75)	2.31
Tocchetto 2023	0.54	(0.43, 0.65)	2.45
Xu 2020	0.06	(0.05, 0.07)	2.53
**Overall**	0.37	(0.29, 0.45)	
**Persistent Depressive Disorder**			
Annagür 2014	0.05	(0.02, 0.11)	10.43
Caumo 2022	0.69	(0.54, 0.80)	11.33
Ciaramella 2015	0.09	(0.07, 0.12)	11.94
Ciaramella 2015_B	0.05	(0.04, 0.08)	11.82
Gündüz 2019b	0.09	(0.04, 0.18)	10.63
Muharam 2022	0.00	(0.00, 0.05)	4.64
Neikrug 2017	0.08	(0.05, 0.11)	11.75
Osório 2016	0.02	(0.00, 0.13)	6.67
Radat 2013	0.01	(0.00, 0.04)	8.66
Xu 2020	0.04	(0.03, 0.04)	12.13
**Overall**	0.06	(0.03, 0.13)	

© 2025 Aaron RV et al. *JAMA Network Open.*

eTable 6. Pooled Prevalence of Anxiety Disorders

Study ID	Event Rate	95% CI	Weight
**Generalized Anxiety Disorder**			
Annagür 2014	0.21	(0.15, 0.30)	4.31
Asih 2016	0.15	(0.12, 0.18)	4.49
Bucourt 2021	0.40	(0.27, 0.54)	4.17
Bucourt 2021_B	0.04	(0.01, 0.16)	2.94
Bucourt 2021_C	0.06	(0.02, 0.18)	3.31
Campbell 2015	0.09	(0.08, 0.10)	4.54
Caumo 2022	0.35	(0.23, 0.50)	4.16
Ciaramella 2015	0.17	(0.13, 0.20)	4.48
Ciaramella 2015_B	0.14	(0.11, 0.17)	4.47
Csupak 2018	0.04	(0.04, 0.05)	4.54
Csupak 2018_B	0.06	(0.05, 0.07)	4.54
Emery 2014	0.38	(0.27, 0.51)	4.24
Gündüz 2018	0.62	(0.48, 0.74)	4.20
Gündüz 2018_B	0.29	(0.16, 0.45)	3.97
Gündüz 2019	0.09	(0.04, 0.19)	3.81
Gündüz 2019b	0.07	(0.03, 0.17)	3.71
Häuser 2015	0.90	(0.84, 0.94)	4.24
Muharam 2022	0.21	(0.15, 0.28)	4.38
Neikrug 2017	0.17	(0.13, 0.22)	4.45
Parisi 2022	0.17	(0.12, 0.22)	4.42
Radat 2013	0.12	(0.08, 0.18)	4.32
Sohn 2016	0.16	(0.07, 0.32)	3.64
Tocchetto 2023	0.28	(0.19, 0.38)	4.29
Xu 2020	0.01	(0.01, 0.01)	4.37
**Overall**	0.17	(0.12, 0.23)	
**Panic Disorder**			
Annagür 2014	0.08	(0.04, 0.15)	7.26
Asih 2016	0.09	(0.07, 0.12)	7.98
Ciaramella 2015	0.16	(0.13, 0.20)	8.02
Ciaramella 2015_B	0.16	(0.13, 0.20)	8.03
Emery 2014	0.22	(0.13, 0.34)	7.41
Gündüz 2019	0.06	(0.02, 0.15)	6.41
Gündüz 2019b	0.06	(0.02, 0.15)	6.41
Lee 2014	0.03	(0.00, 0.16)	4.00
Muharam 2022	0.04	(0.02, 0.09)	7.08
Neikrug 2017	0.02	(0.01, 0.05)	6.97
Osório 2016	0.16	(0.08, 0.29)	7.08
Proctor 2013	0.09	(0.06, 0.14)	7.73
Radat 2013	0.14	(0.10, 0.20)	7.80
Xu 2020	0.01	(0.01, 0.01)	7.83
**Overall**	0.07	(0.04, 0.12)	
**Social Anxiety Disorder**			
Osório 2016	0.20	(0.11, 0.33)	14.77
Ciaramella 2015	0.04	(0.02, 0.06)	15.29
Gündüz 2019b	0.03	(0.01, 0.11)	12.06
Ciaramella 2015_B	0.02	(0.01, 0.04)	15.04
Radat 2013	0.02	(0.01, 0.05)	13.11
Xu 2020	0.01	(0.00, 0.01)	15.45
Muharam 2022	0.00	(0.00, 0.05)	7.15
Annagür 2014	0.00	(0.00, 0.07)	7.14
**Overall**	0.02	(0.01, 0.06)	

© 2025 Aaron RV et al. *JAMA Network Open.*

eTable 7. Sensitivity Analysis by Measure Type

Moderator	K	Prevalence	95% CI	Q	p	I2
**Clinical Symptoms**						
**Depression Symptoms**						
Zung SDS	8	44.72%	(31.51%, 58.73%)	75.96	< .001	90.78%
BDI	58	44.48%	(38.46%, 50.66%)	1435.69	< .001	96.03%
PHQ	105	41.62%	(37.63%, 45.72%)	8154.81	< .001	98.72%
CES-D	25	41.38%	(31.49%, 52.01%)	2184.25	< .001	98.90%
DASS	11	39.28%	(29.63%, 49.85%)	3513.19	< .001	99.72%
GDS	15	36.80%	(25.31%, 50.02%)	590.86	< .001	97.63%
HAM-D	7	35.93%	(26.69%, 46.34%)	25.97	< .001	76.90%
HADS-D	108	35.22%	(32.98%, 37.52%)	2173.18	< .001	95.08%
HDRS	4	35.21%	(12.76%, 66.88%)	99.87	< .001	97.00%
PROMIS	5	34.48%	(17.27%, 57.02%)	251.37	< .001	98.41%
Total Between Group Variance				***14.90***	***< .001***	
**Anxiety Symptoms**						
HADS-A	95	44.53%	(41.99%, 47.09%)	1763.11	< .001	94.67%
DASS	12	43.13%	(37.93%, 48.50%)	2225.10	< .001	99.51%
BAI	12	39.13%	(28.95%, 50.37%)	107.39	< .001	89.76%
PROMIS	4	38.23%	(24.31%, 54.39%)	101.63	< .001	97.05%
HAM-A	5	37.56%	(28.17%, 47.98%)	13.63	< .001	70.66%
GAD	48	33.58%	(29.18%, 38.28%)	2970.58	< .001	98.42%
STAI-T	4	30.79%	(8.54%, 67.94%)	69.48	< .001	95.68%
Total Between Group Variance				***17.44***	***0.01***	
**Clinical Diagnoses**						
**Major Depressive Disorder**						
MINI	11	44.34%	(31.99%, 57.43%)	265.32	0	96.23%
SCID	20	37.70%	(30.57%, 45.40%)	417.94	0	95.45%
Total Between Group Variance				***0.77***	***0.381***	
**Persistent Depressive Disorder**						
MINI	5	6.81%	(1.59%, 24.90%)	119.28	<.001	96.65%
SCID	4	6.87%	(4.91%, 9.53%)	3.05	0.385	1.49%
Total Between Group Variance				***0.00***	***0.991***	
**Generalized Anxiety Disorder**						
CIDI	4	3.93%	(2.29%, 6.66%)	180.54	<.001	98.34%
MINI	10	18.54%	(14.10%, 23.99%)	48.00	<.001	81.25%
SCID	8	19.68%	(12.64%, 29.34%)	63.04	<.001	88.90%
Total Between Group Variance				***30.03***	***<.001***	
**Panic Disorder**						
MINI	4	13.01%	(9.33%, 17.85%)	12.79	<.001	76.54%
SCID	7	6.70%	(4.13%, 10.71%)	18.72	<.001	67.95%
Total Between Group Variance				***5.14***	***<.001***	

© 2025 Aaron RV et al. *JAMA Network Open.*

eFigure 3. Risk of Bias Overview

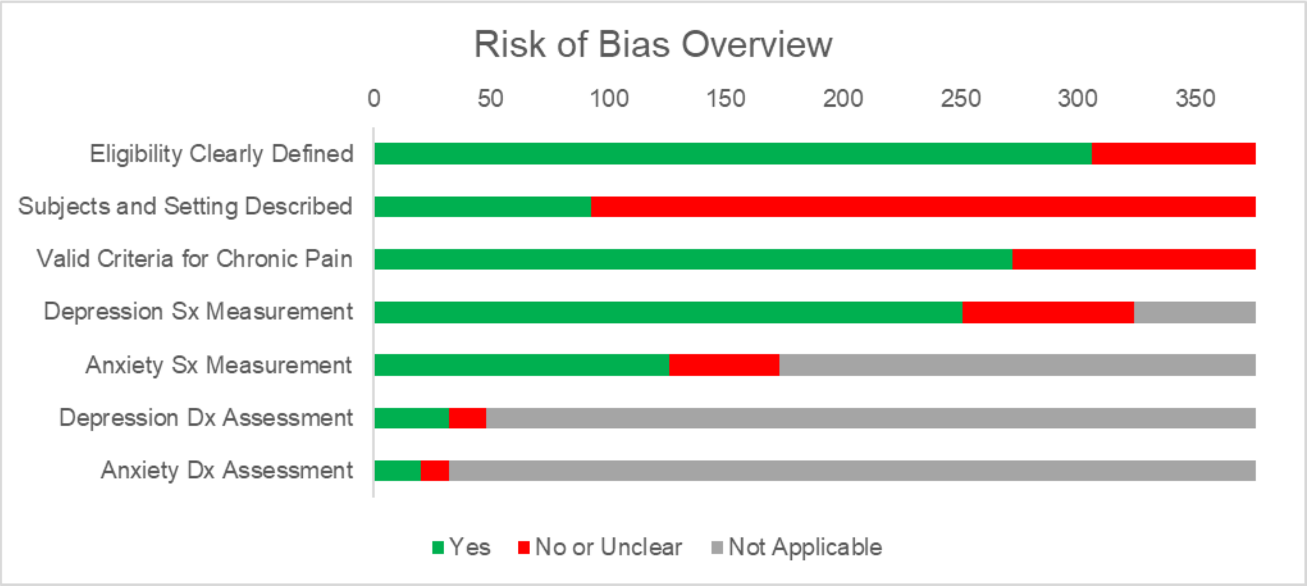

Note. Sx = symptoms; Dx = Diagnosis.

© 2025 Aaron RV et al. *JAMA Network Open.*

eTable 8. Individual Study Risk of Bias Score

Study ID	ROB	Study ID	ROB	Study ID	ROB	Study ID	ROB
Aagaard 2023	80%	Andersen 2016	0%	Barton 2021	75%	Campbell 2015b	60%
Abrams 2013	75%	Annagür 2014	80%	Bayrak 2020	50%	Campos 2021	80%
Ahmad 2023	0%	Antaky 2017	75%	Bayram 2014	80%	Cappe 2021	40%
Ajo 2017	40%	Antunes 2013	75%	Bell 2016	100%	Carleton 2022	40%
Åkerblom 2020	50%	Ardigo 2016	80%	Bilen 2022	80%	Carrillo-de-la-Peña 2015	50%
Akui 2022	40%	Areias 2023	75%	Birch 2022	80%	Carrillo-Izquierdo 2018	50%
Alamam 2019	100%	Argoff 2016	40%	Blakey 2018	50%	Carta 2018	80%
Alberts 2020	100%	Asada 2022	25%	Bohn 2013	100%	CasteloAzevedo 2021	75%
Alciati 2018	100%	Aşçıbaşı 2022	40%	Botros 2022	40%	Caumo 2022	40%
Alciati 2020	60%	Asih 2014	75%	Boukhira 2021	100%	Chaabouni 2022	75%
Alhalal 2021	75%	Asih 2016	80%	Bravo 2019	80%	Chahal-Kummen 2019	40%
Allaire 2018	60%	Asseyer 2021	25%	BromleyMilton 2013	0%	Chahal-Kummen 2023	20%
Al-Maharbi 2018	75%	Ataoglu 2013	50%	Bruce 2021	50%	Challa 2017	75%
Aloush 2021	60%	Avluk 2014	75%	Brunner 2023	50%	Chana 2021	60%
AlShukaili 2022	75%	Bagnato 2014	75%	Bryant 2016	80%	Chang 2018	75%
AlvesRodrigues 2022	40%	Bailey 2020	75%	Bucourt 2021	80%	Cheatle 2023	100%
Amatsu 2022	75%	Bapir 2023	33%	Calandre 2022	75%		
Amital 2014	25%	Barbosa 2016	80%	Calderón 2016	40%		
Ammitzbøll 2021	50%	Barjandi 2021	75%	Calpin 2017	80%		
		Barth 2014	25%	Campbell 2015	80%		

© 2025 Aaron RV et al. *JAMA Network Open.*

Study ID	ROB	Study ID	ROB	Study ID	ROB	Study ID	ROB
Chen 2022	75%	DelPozo-Cruz 2017	75%	Falling 2021	100%	Gavilan-Carrera 2022	50%
Cheng 2018	100%	deMoraesVieira 2014	75%	Farin 2015	60%	Gebauer 2019	50%
Cheng 2022	100%	Depintor 2016	40%	Farzad 2021	80%	Gelonch 2017	75%
Cho 2017	80%	DeRoa 2018	20%	Feingold 2017	40%	Gelonch 2018	75%
Choi 2018	50%	Doherty 2017	25%	Feingold 2018	60%	Gentili 2019	100%
Chytas 2023	50%	Domenech 2013	80%	Feingold 2020	60%	Gerdle 2019	50%
Ciaramella 2015	40%	DosSantosProença 2023	80%	Feliu-Soler 2017	75%	Gerra 2021	25%
Civelek 2022	75%	Dougados 2017	50%	Fiegl 2019	40%	Gisev 2019	80%
Clark 2018	75%	Drummond 2013	20%	Filippon 2013	100%	Giummarra 2020	100%
Clark 2019	50%	D'Souza 2020	75%	Finn 2018	80%	Goesling 2015	75%
Cody 2019	75%	Dubois-Mendes 2021	80%	Fischer-Jbali 2022	80%	Goesling 2015b	50%
Conway 2020	100%	Dumolard 2023	40%	Flores-Curiel 2023	75%	Goesling 2018	75%
Corriger 2022	40%	Durán 2021	75%	Foti 2022	0%	Gomes 2023	50%
Costa 2023	40%	Ecija 2022	40%	Frede 2023	50%	Gorpynchenko 2021	40%
Csupak 2018	75%	Edit 2013	75%	Freo 2021	40%	Gota 2017	100%
DaLuz 2018	100%	Elrashidi 2018	75%	Garaigordobil 2016	0%	Govind 2020	80%
Damci 2022	80%	Emery 2014	40%	Garbi 2014	75%	Greenberg 2022	75%
Davis 2014	75%	Englbrecht 2019	50%	GarciaCarrasco 2013	50%	Gündüz 2018	40%
Dear 2023	80%	Eriksen 2021	75%	Garnæs 2022	80%	Gündüz 2019	40%
deHeer 2017	80%					Gündüz 2019b	40%
DeLaRosa 2023	75%						

© 2025 Aaron RV et al. *JAMA Network Open.*

Study ID	ROB	Study ID	ROB	Study ID	ROB	Study ID	ROB
Gupta 2014	50%	Iannuccelli 2021	60%	Karafin 2018	50%	Lerman 2015	40%
Gupta 2016	75%	Inanc 2014	0%	Karas 2020	75%	Lin 2017	25%
Hållstam 2017	100%	Ingegnoli 2022	0%	Kazemi 2013	60%	Lindemann 2023	80%
Hamdi 2021	50%	Islam 2023	80%	Kec 2022	100%	Liu 2018	75%
Han 2019	0%	Jain 2013	25%	Kha 2020	50%	Longo 2022	50%
Hansdorfer-Korzon 2016	80%	Jaiswal 2016	50%	Khazen 2021	50%	Lopez 2013	25%
Hardy 2022	20%	Janela 2022	75%	KibuneNagasako 2016	40%	Luciano 2014	75%
Harrison 2016	25%	Järemo 2022	100%	Kim 2021	60%	Luque-Reca 2021	75%
Hasuo 2020	80%	Jedel 2020	75%	Knaster 2016	50%	Mahendru 2021	80%
Häuser 2014	25%	Jegan 2017	75%	Koh 2014	75%	Mahlich 2019	75%
Häuser 2015	60%	Jensen 2016	20%	Kosson 2018	60%	Mameli 2014	40%
Häuser 2015b	100%	Jiao 2021	75%	Kroenke 2020	25%	Mann 2017	100%
Hoban 2015	80%	Jimenez-Rodríguez 2014	75%	Lamerato 2023	100%	Mantyselka 2017	25%
Hochman 2013	75%	Jin 2021	50%	Landefeld 2017	100%	Marcum 2014	100%
Hogestol 2017	80%	Jindal 2021	75%	Larance 2019	60%	Marino 2021	20%
Holloway 2017	50%	John 2022	0%	Laurent 2014	40%	Mattila-Rautiainen 2023	50%
Honda 2022	75%	Jouini 2014	40%	Laurent 2022	60%	Mayer 2013	100%
Hong 2018	50%	Joyce 2021	100%	Lee 2014	80%	McCracken 2023	50%
Hudak 2022	50%	Kadimpati 2015	75%	Lee 2020	75%	McWilliams 2017	50%
Hughes 2019	25%	Kallusky 2023	80%	León-Suárez 2023	40%	Mehraban 2014	60%

© 2025 Aaron RV et al. *JAMA Network Open.*

Study ID	ROB	Study ID	ROB	Study ID	ROB	Study ID	ROB
Merlin 2015	50%	Phillips 2014	80%	Reiter 2017	80%	Santos 2017	80%
Midbari 2016	50%	PicchiantiDiamanti 2020	80%	Reiter 2018	100%	Scherrer 2015	75%
Miller-Matero 2016	60%	Pinto 2022	80%	Rexelius 2020	25%	Schmukler 2023	0%
Moraes 2021	80%	Plinsinga 2020	80%	Rezaei 2014	75%	Schroeter 2015	40%
Morlà 2021	75%	Plinsinga 2023	80%	Rice 2016	80%	Schwab 2022	80%
Muharam 2022	40%	Porru 2023	25%	Robinson 2013	100%	Schwarm 2021	40%
Murata 2019	50%	Poulin 2016	100%	Rogal 2015	100%	Seed 2015	75%
MurMartí 2017	100%	Prateepavanich 2018	75%	Rogers 2021	75%	Seekatz 2016	25%
Mustonen 2019	60%	Preti 2019	75%	Rojas 2021	75%	Sener 2013	75%
Nacak 2021	75%	Priol 2023	40%	Rometsch 2022	100%	Sengupta 2023	75%
Neblett 2016	75%	Proctor 2013	60%	Rometsch 2023	60%	Shamji 2016	40%
Neikrug 2017	20%	Puto 2023	75%	Rouch 2021	75%	Sharma 2022	60%
Nguyen 2021	75%	Quidé 2022	75%	Rouch 2023	80%	Shebeshi 2023	100%
Norman-Nott 2022	75%	Quinlan 2021	25%	Rovner 2017	75%	Shmagel 2016	75%
Offenbaecher 2013	80%	Racine 2014	75%	Roy 2022	50%	Si 2019	75%
Ojeda 2018	80%	Racine 2017	75%	RusMakovec 2015	20%	Silva 2021	80%
Osório 2016	80%	Radat 2013	80%	Rusu 2016	80%	Siqueira-Campos 2019	80%
Parisi 2022	60%	Rapariz-González 2014	75%	Saariaho 2015	75%	Siqueira-Campos 2022	100%
Pasin 2023	75%	Rayner 2016	50%	Sachau 2023	80%	Sitges 2018	75%
Peilot 2018	50%			Sagheer 2013	40%		
Penacoba 2023	75%			Saglam 2022	75%		

© 2025 Aaron RV et al. *JAMA Network Open.*

Study ID	ROB	Study ID	ROB	Study ID	ROB	Study ID	ROB
Slawek 2021	75%	Tanaka 2022	80%	Vance 2018	75%	Widenka 2021	25%
Sleurs 2020	80%	Tardif 2023	60%	vandenBerk-Clark 2017	75%	Wildes 2020	50%
Sohn 2016	40%	Tatebe 2016	50%	vanEeden 2023	80%	Wilson 2015	75%
Song 2015	75%	Taylor 2021	80%	VanOvermeire 2022	50%	Wilson 2022	100%
Soriano-Maldonado 2015	50%	Teixido-Abiol 2022	75%	VanRyckeghem 2013	75%	Wolfe 2013	60%
Souza 2021	100%	Terassi 2020	75%	Varinen 2019	50%	Wong 2017	75%
Sparkes 2015	50%	Tetsunaga 2013	50%	Vilalta-Abella 2015	75%	Wright 2017	75%
Stefani 2019	75%	Teychenne 2019	75%	Villafaina 2019	75%	Xu 2020	40%
Stehlik 2018	50%	Thakral 2018	75%	Voute 2023	40%	Yadav 2023	75%
Stein 2015	100%	ThiNguy 2022	75%	Vukojević 2022	20%	Yamada 2022	60%
Steiner 2017	75%	Thomas 2022	50%	Wadley 2019	100%	Yin 2023	75%
Stokholm 2022	80%	Thompson 2023	75%	Wadley 2020	100%	Yu 2021	100%
Subramaniam 2013	40%	Tocchetto 2023	83%	Wahlman 2014	50%	Zaidel 2021	50%
SundaraRajan 2017	60%	Tsuji 2016	50%	Weingarten 2016	75%	Zakrzewska 2017	75%
Sundstrom 2023	75%	Uebelacker 2015	100%	Whitlock 2017	25%	Zambelli 2021	100%
		Upadhyaya 2023	80%			Zubatsky 2020	75%

Note. Risk of Bias (ROB) is interpreted as high (0-49%), medium (50-74%), or low (75-100%).

© 2025 Aaron RV et al. *JAMA Network Open.*

eReferences.

Note that the number of referenced papers is greater than the number of studies included, due to several papers being published from the same study. When multiple papers were identified from the same study, the papers were merged in Covidence to reflect a single record (or “study ID”), such that the data from individual publications was preserved and extracted under a single record.

1. Aagaard A, Ravn SL, Andersen TE, Vaegter HB. Interpretation of the Patient Health Questionnaire 9 in High-Impact Chronic Pain: Do We Measure Depressive Symptoms the Way We Think? *Clin J Pain*. 2023;39(10):501-515. doi:https://dx.doi.org/10.1097/AJP.0000000000001142
2. Abrams DI, Dolor R, Roberts R, et al. The BraveNet prospective observational study on integrative medicine treatment approaches for pain. *BMC Altern Med*. 2013;13:146. doi:https://dx.doi.org/10.1186/1472-6882-13-146
3. Ahmad H, Gul Y, Dawar SA, Riaz Q, Ullah K, Kamal A. Association of Depression in Patients with Fibromyalgia Syndrome. *Pakistan Journal of Medical and Health Sciences*. 2023;17(2):762-765. doi:10.53350/pjmhs2023172762
4. Ajo R, Segura A, Inda MD, et al. Erectile dysfunction in patients with chronic pain treated with opioids. *Med Clin (Barc)*. 2017;149(2):49-54. doi:https://dx.doi.org/10.1016/j.medcli.2016.12.038
5. Akerblom S, Perrin S, Fischer MR, McCracken LM. Treatment outcomes in group-based cognitive behavioural therapy for chronic pain: An examination of PTSD symptoms. *Eur J Pain*. 2020;24(4):807-817. doi:https://dx.doi.org/10.1002/ejp.1530
6. Akui C, Kimura T, Hirose M. Associations between insomnia and central sensitization in cancer survivors undergoing opioid therapy for chronic cancer pain: A STROBE-compliant prospective cohort study. *Medicine (United States)*. 2022;101(38):E30845. doi:10.1097/MD.0000000000030845
7. Al Shukaili M, Al Alawi M, Al Huseini S, et al. Exploring Factors Associated With Depressive Symptoms Among Patients With Chronic Pain: A Cross-Sectional Multicenter Study. *J Nerv Ment Dis*. 2022;210(1):45-53. doi:https://dx.doi.org/10.1097/NMD.0000000000001409
8. Al-Maharbi S, Abolkhair AB, Al Ghamdi H, et al. Prevalence of depression and its association with sociodemographic factors in patients with chronic pain: A cross-sectional study in a tertiary care hospital in Saudi Arabia. *Saudi J Anaesth*. 2018;12(3):419-425. doi:https://dx.doi.org/10.4103/sja.SJA_771_17
9. Alamam DM, Moloney N, Leaver A, Alsobayel HI, Mackey MG. Pain Intensity and Fear Avoidance Explain Disability Related to Chronic Low Back Pain in a Saudi Arabian Population. *Spine*. 2019;44(15):E889-E898. doi:https://dx.doi.org/10.1097/BRS.0000000000003002
10. Alberts NM, Leisenring WM, Flynn JS, et al. Wearable Respiratory Monitoring and Feedback for Chronic Pain in Adult Survivors of Childhood Cancer: A Feasibility Randomized Controlled Trial From the Childhood Cancer Survivor Study. *JCO Clin Cancer Inform*. 2020;4:1014-1026. doi:https://dx.doi.org/10.1200/CCI.20.00070
11. Alciati A, Atzeni F, Caldirola D, Perna G, Sarzi-Puttini P. The Co-Morbidity between Bipolar and Panic Disorder in Fibromyalgia Syndrome. *J*. 2020;9(11):10. doi:https://dx.doi.org/10.3390/jcm9113619
12. Alciati A, Atzeni F, Grassi M, et al. Features of mood associated with high body weight in females with fibromyalgia. *Compr Psychiatry*. 2018;80:57-64. doi:https://dx.doi.org/10.1016/j.comppsych.2017.08.006
13. Alhalal EA, Alhalal IA, Alaida AM, Alhweity SM, Alshojaa AY, Alfaori AT. Effects of chronic pain on sleep quality and depression: A cross-sectional study. *Saudi Med J*. 2021;42(3):315-323. doi:https://dx.doi.org/10.15537/smj.42.3.20200768
14. Allaire C, Williams C, Bodmer-Roy S, et al. Chronic pelvic pain in an interdisciplinary setting: 1-year prospective cohort. *Am J Obstet Gynecol*. 2018;218(1):114.e1-114.e12. doi:https://dx.doi.org/10.1016/j.ajog.2017.10.002
15. Aloush V, Gurfinkel A, Shachar N, Ablin JN, Elkana O. Physical and mental impact of COVID-19 outbreak on fibromyalgia patients. *Clin Exp Rheumatol*. 2021;39 Suppl 130(3):108-114. doi:https://dx.doi.org/10.55563/clinexprheumatol/rxk6s4
16. Alves Rodrigues T, de Oliveira E, Morais Costa B, Tajra Mualem Araujo RL, Batista Santos Garcia J. Is There a Difference in Fear-Avoidance, Beliefs, Anxiety and Depression Between Post-Surgery and Non-Surgical Persistent Spinal Pain Syndrome Patients? *J Pain Res*. 2022;15:1707-1717. doi:https://dx.doi.org/10.2147/JPR.S348146
17. Amatsu T, Tsujiguchi H, Hara A, et al. Relationship between Alcohol Intake and Chronic Pain with Depressive Symptoms: A Cross-Sectional Analysis of the Shika Study. *Int J Environ Res Public Health*. 2022;19(4):11. doi:https://dx.doi.org/10.3390/ijerph19042024
18. Amital H, Agmon-Levin N, Shoenfeld N, et al. Olfactory impairment in patients with the fibromyalgia syndrome and systemic sclerosis. *Immunol Res*. 2014;60(2-3):201-7. doi:https://dx.doi.org/10.1007/s12026-014-8573-5
19. Ammitzbøll C, Andersen JB, Vils SR, et al. Isolation, Behavioral Changes, and Low Seroprevalence of SARS-CoV-2 Antibodies in Patients With Systemic Lupus Erythematosus or Rheumatoid Arthritis. *Arthritis Care Res (Hoboken)*. 2021;doi:10.1002/acr.24716
20. Andersen TE, Vaegter HB. A 13-Weeks Mindfulness Based Pain Management Program Improves Psychological Distress in Patients with Chronic Pain Compared with Waiting List Controls. *Clin Pract Epidemol Ment Health*. 2016;12:49-

© 2025 Aaron RV et al. *JAMA Network Open.*

58.
21. Annagur BB, Uguz F, Apiliogullari S, Kara I, Gunduz S. Psychiatric disorders and association with quality of sleep and quality of life in patients with chronic pain: a SCID-based study. *Pain Med*. 2014;15(5):772-81. doi:https://dx.doi.org/10.1111/pme.12390
22. Antaky E, Lalonde L, Schnitzer ME, et al. Identifying heavy health care users among primary care patients with chronic non-cancer pain. *Can J Pain*. 2017;1(1):22-36. doi:https://dx.doi.org/10.1080/24740527.2017.1326088
23. Antunes RS, de Macedo BG, Amaral Tda S, Gomes Hde A, Pereira LS, Rocha FL. Pain, kinesiophobia and quality of life in chronic low back pain and depression. 2013;1(1):27-9.
24. Ardigo S, Herrmann FR, Moret V, et al. Hypnosis can reduce pain in hospitalized older patients: a randomized controlled study. *BMC geriatr*. 2016;16:14. doi:https://dx.doi.org/10.1186/s12877-016-0180-y
25. Areias AC, Costa F, Janela D, et al. Impact on productivity impairment of a digital care program for chronic low back pain: A prospective longitudinal cohort study. *Musculoskelet Sci Pract*. 2023;63:102709. doi:https://dx.doi.org/10.1016/j.msksp.2022.102709
26. Argoff CE, Clair A, Emir B, Whalen E, Ortiz M, Pauer L. Prior Opioid Use Does Not Impact the Response to Pregabalin in Patients With Fibromyalgia. *Clin J Pain*. 2016;32(7):555-61. doi:https://dx.doi.org/10.1097/AJP.0000000000000232
27. Asada M, Shibata M, Hirabayashi N, et al. Association between chronic low back pain and regional brain atrophy in a Japanese older population: the Hisayama Study. *Pain*. 2022;15:15. doi:https://dx.doi.org/10.1097/j.pain.0000000000002612
28. Aşçıbaşı K, Özçete ZA. Evaluation of psychiatric symptoms in patients diagnosed with fibromyalgia during COVID-19 pandemic: a cross-sectional study. *European Research Journal*. 2022;8(6):762-770. doi:10.18621/eurj.1125754
29. Asih S, Mayer TG, Bradford EM, et al. The Potential Utility of the Patient Health Questionnaire as a Screener for Psychiatric Comorbidity in a Chronic Disabling Occupational Musculoskeletal Disorder Population. *Pain pract*. 2016;16(2):168-74. doi:https://dx.doi.org/10.1111/papr.12275
30. Asih S, Neblett R, Mayer TG, Brede E, Gatchel RJ. Insomnia in a chronic musculoskeletal pain with disability population is independent of pain and depression. *Spine J*. 2014;14(9):2000-7. doi:https://dx.doi.org/10.1016/j.spinee.2013.11.052
31. Asseyer S, Henke E, Trebst C, et al. Pain, depression, and quality of life in adults with MOG-antibody–associated disease. *European Journal of Neurology*. 2021;28(5):1645-1658. doi:10.1111/ene.14729
32. Ataoglu E, Tiftik T, Kara M, Tunc H, Ersoz M, Akkus S. Effects of chronic pain on quality of life and depression in patients with spinal cord injury. *Spinal Cord*. 2013;51(1):23-6. doi:https://dx.doi.org/10.1038/sc.2012.51
33. Avluk OC, Gurcay E, Gurcay AG, Karaahmet OZ, Tamkan U, Cakci A. Effects of chronic pain on function, depression, and sleep among patients with traumatic spinal cord injury. *Ann Saudi Med*. 2014;34(3):211-6. doi:https://dx.doi.org/10.5144/0256-4947.2014.211
34. Bagnato G, De Andres I, Sorbara S, et al. Pain threshold and intensity in rheumatic patients: correlations with the Hamilton Depression Rating scale. *Clinical Rheumatology*. 2014:1-7. doi:10.1007/s10067-013-2477-y
35. Bailey JF, Agarwal V, Zheng P, et al. Digital Care for Chronic Musculoskeletal Pain: 10,000 Participant Longitudinal Cohort Study. *J Med Internet Res*. 2020;22(5):e18250. doi:https://dx.doi.org/10.2196/18250
36. Bapir L, Erridge S, Nicholas M, et al. Comparing the effects of medical cannabis for chronic pain patients with and without co-morbid anxiety: A cohort study. *Expert rev*. 2023;23(3):281-295. doi:https://dx.doi.org/10.1080/14737175.2023.2181696
37. Barbosa Hde F, Nogueira AA, e Silva JC, Poli Neto OB, dos Reis FJ. The Influence of Education and Depression on Autonomy of Women with Chronic Pelvic Pain: A Cross-sectional Study. *Rev*. 2016;38(1):47-52. doi:https://dx.doi.org/10.1055/s-0035-1570107
38. Barjandi G, Kosek E, Hedenberg-Magnusson B, Velly AM, Ernberg M. Comorbid Conditions in Temporomandibular Disorders Myalgia and Myofascial Pain Compared to Fibromyalgia. *J*. 2021;10(14):16. doi:https://dx.doi.org/10.3390/jcm10143138
39. Barth KS, Balliet W, Pelic CM, et al. Screening for current opioid misuse and associated risk factors among patients with chronic nonalcoholic pancreatitis pain. *Pain Med*. 2014;15(8):1359-64. doi:https://dx.doi.org/10.1111/pme.12403
40. Barton JL, Markwardt S, Niederhausen M, et al. Are We on the Same Page?: A Cross-Sectional Study of Patient-Clinician Goal Concordance in Rheumatoid Arthritis. *Arthritis Care Res (Hoboken)*. 2021;doi:10.1002/acr.24794
41. Bayrak M. Metabolic syndrome, depression, and fibromyalgia syndrome prevalence in patients with irritable bowel syndrome: A case-control study. *Medicine (Baltimore)*. 2020;99(23):e20577. doi:https://dx.doi.org/10.1097/MD.0000000000020577
42. Bayram K, Erol A. Childhood Traumatic Experiences, Anxiety, and Depression Levels in Fibromyalgia and Rheumatoid Arthritis. 2014;1(4):344-349.
43. Bell JA, daCosta DiBonaventura M, Witt EA, Ben-Joseph R, Reeve BB. Use of the SF-36v2 Health Survey as a

Screen for Risk of Major Depressive Disorder in a US Population-based Sample and Subgroup With Chronic Pain. *Medical Care*. 2016;doi:10.1097/MLR.0000000000000617
44. Bell JA, daCosta DiBonaventura M, Witt EA, Ben-Joseph R, Reeve BB. Use of the SF-36v2 Health Survey as a Screen for Risk of Major Depressive Disorder in a US Population-based Sample and Subgroup With Chronic Pain. *Med Care*. 2017;55(2):111-116. doi:https://dx.doi.org/10.1097/MLR.0000000000000617
45. Bilen A, Kucukkepeci H. Pain Intensity, Depression, and Anxiety Levels Among Patients With Chronic Pain During COVID-19 Pandemic. *J Nerv Ment Dis*. 2022;210(4):270-275. doi:https://dx.doi.org/10.1097/NMD.0000000000001466
46. Birch N, Graham J, Ozolins C, Kumarasinghe K, Almesfer F. Home-Based EEG Neurofeedback Intervention for the Management of Chronic Pain. *Front*. 2022;3:855493. doi:https://dx.doi.org/10.3389/fpain.2022.855493
47. Blakey SM, Wagner HR, Naylor J, et al. Chronic Pain, TBI, and PTSD in Military Veterans: A Link to Suicidal Ideation and Violent Impulses? *J Pain*. 2018;19(7):797-806. doi:https://dx.doi.org/10.1016/j.jpain.2018.02.012
48. Bohn D, Bernardy K, Wolfe F, Hauser W. The association among childhood maltreatment, somatic symptom intensity, depression, and somatoform dissociative symptoms in patients with fibromyalgia syndrome: a single-center cohort study. *J Trauma Dissociation*. 2013;14(3):342-58. doi:https://dx.doi.org/10.1080/15299732.2012.736930
49. Botros J, Gornitsky M, Samim F, der Khatchadourian Z, Velly AM. Back and neck pain: A comparison between acute and chronic pain-related Temporomandibular Disorders. *Can J Pain*. 2022;6(1):112-120. doi:https://dx.doi.org/10.1080/24740527.2022.2067032
50. Boukhira I, Jidane S, Kharbach A, Belyamani L. Chronic pain assessment in moroccan hemodialysis population. *Electronic Journal of General Medicine*. 2021;18(6)doi:10.29333/ejgm/11312
51. Bravo C, Skjaerven LH, Espart A, Guitard Sein-Echaluce L, Catalan-Matamoros D. Basic Body Awareness Therapy in patients suffering from fibromyalgia: A randomized clinical trial. *Physiother*. 2019;35(10):919-929. doi:https://dx.doi.org/10.1080/09593985.2018.1467520
52. Bromley Milton M, Borsbo B, Rovner G, Lundgren-Nilsson A, Stibrant-Sunnerhagen K, Gerdle B. Is Pain Intensity Really That Important to Assess in Chronic Pain Patients? A Study Based on the Swedish Quality Registry for Pain Rehabilitation (SQRP). *PLoS ONE*. 2013;8(6):e65483. doi:https://dx.doi.org/10.1371/journal.pone.0065483
53. Bruce BK, Allman ME, Rivera FA, et al. Opioid Use in Fibromyalgia Continues Despite Guidelines That Do Not Support Its Efficacy or Risk. *J*. 2021;27(5):187-193. doi:https://dx.doi.org/10.1097/RHU.0000000000001273
54. Brunner WM, Pullyblank K, Scribani MB, Krupa N, Wyckoff L. Remote delivery of self-management education workshops for adults with chronic pain, 2020-2021. *Chronic Illn*. 2023:17423953231181408. doi:https://dx.doi.org/10.1177/17423953231181408
55. Bryant C, Cockburn R, Plante AF, Chia A. The psychological profile of women presenting to a multidisciplinary clinic for chronic pelvic pain: high levels of psychological dysfunction and implications for practice. *J Pain Res*. 2016;9:1049-1056.
56. Bucourt E, Martaille V, Goupille P, et al. A Comparative Study of Fibromyalgia, Rheumatoid Arthritis, Spondyloarthritis, and Sjogren's Syndrome; Impact of the Disease on Quality of Life, Psychological Adjustment, and Use of Coping Strategies. *Pain Med*. 2021;22(2):372-381. doi:https://dx.doi.org/10.1093/pm/pnz255
57. Calandre EP, Garcia-Leiva JM, Ordonez-Carrasco JL. Psychosocial Variables and Healthcare Resources in Patients with Fibromyalgia, Migraine and Comorbid Fibromyalgia and Migraine: A Cross-Sectional Study. *Int J Environ Res Public Health*. 2022;19(15):23. doi:https://dx.doi.org/10.3390/ijerph19158964
58. Calderon E, Calderon-Seoane ME, Garcia-Hernandez R, Torres LM. 5% Lidocaine-medicated plaster for the treatment of chronic peripheral neuropathic pain: complex regional pain syndrome and other neuropathic conditions. *J Pain Res*. 2016;9:763-770.
59. Calpin P, Imran A, Harmon D. A Comparison of Expectations of Physicians and Patients with Chronic Pain for Pain Clinic Visits. *Pain pract*. 2017;17(3):305-311. doi:https://dx.doi.org/10.1111/papr.12428
60. Campbell G, Darke S, Bruno R, Degenhardt L. The prevalence and correlates of chronic pain and suicidality in a nationally representative sample. *Aust N Z J Psychiatry*. 2015;49(9):803-11. doi:https://dx.doi.org/10.1177/0004867415569795
61. Campbell G, Nielsen S, Bruno R, et al. The Pain and Opioids IN Treatment study: characteristics of a cohort using opioids to manage chronic non-cancer pain. *Pain*. 2015;156(2):231-242. doi:https://dx.doi.org/10.1097/01.j.pain.0000460303.63948.8e
62. Campbell G, Nielsen S, Larance B, et al. Pharmaceutical Opioid Use and Dependence among People Living with Chronic Pain: Associations Observed within the Pain and Opioids in Treatment (POINT) Cohort. *Pain Medicine (United States)*. 2015;16(9):1745-1758. doi:10.1111/pme.12773
63. Campos RP, Vazquez I, Vilhena E. Clinical, psychological and quality of life differences in fibromyalgia patients from secondary and tertiary healthcare. *Eur J Pain*. 2021;25(3):558-572. doi:https://dx.doi.org/10.1002/ejp.1694
64. Cappe E, Bolduc M, Vilcoque A, et al. Perceived dyadic coping, anxiety, depression and satisfaction with life of women diagnosed with fibromyalgia. *Psychologie Française*. 2021;66(3):259-271. doi:10.1016/j.psfr.2020.09.002
65. Carrillo-de-la-Pena MT, Trinanes Y, Gonzalez-Villar A, Gomez-Perretta C, Garcia-Larrea L. Filtering out repetitive auditory stimuli in fibromyalgia: a study of P50 sensory gating. *Eur J Pain*. 2015;19(4):576-84.

© 2025 Aaron RV et al. *JAMA Network Open.*

doi:https://dx.doi.org/10.1002/ejp.627
66. Carrillo-Izquierdo MD, Slim M, Hidalgo-Tallon J, Calandre EP. Pelvic floor dysfunction in women with fibromyalgia and control subjects: Prevalence and impact on overall symptomatology and psychosocial function. *Neurourol Urodyn*. 2018;37(8):2702-2709. doi:https://dx.doi.org/10.1002/nau.23723
67. Carta MG, Moro MF, Pinna FL, et al. The impact of fibromyalgia syndrome and the role of comorbidity with mood and post-traumatic stress disorder in worsening the quality of life. *Int J Soc Psychiatry*. 2018;64(7):647-655. doi:https://dx.doi.org/10.1177/0020764018795211
68. Castelo Azevedo D, Carneiro Machado LA, Giatti L, Harter Griep R, Telles RW, Barreto SM. Different Components of Subjective Well-being Are Associated With Chronic Nondisabling and Disabling Knee Pain: ELSA-Brasil Musculoskeletal Cohort. *J*. 2021;27(6S):S301-S307. doi:https://dx.doi.org/10.1097/RHU.0000000000001472
69. Caumo W, Alves RL, Vicuna P, et al. Impact of Bifrontal Home-Based Transcranial Direct Current Stimulation in Pain Catastrophizing and Disability due to Pain in Fibromyalgia: A Randomized, Double-Blind Sham-Controlled Study. *J Pain*. 2022;23(4):641-656. doi:https://dx.doi.org/10.1016/j.jpain.2021.11.002
70. Chaabouni M, Feki W, Moussa N, Bahloul N, Kammoun S. Chronic Pain in Patients with Chronic Obstructive Pulmonary Disease: A Cross Sectional Study. *Tanaffos*. 2022;21(2):167-178.
71. Chahal-Kummen M, Blom-Hogestol IK, Eribe I, Klungsoyr O, Kristinsson J, Mala T. Abdominal pain and symptoms before and after Roux-en-Y gastric bypass. *BJS open*. 2019;3(3):317-326. doi:https://dx.doi.org/10.1002/bjs5.50148
72. Chahal-Kummen M, Vage V, Kristinsson JA, Mala T. Chronic abdominal pain and quality of life after Roux-en-Y gastric bypass and sleeve gastrectomy - a cross-cohort analysis of two prospective longitudinal observational studies. *Surg*. 2023;19(8):819-829. doi:https://dx.doi.org/10.1016/j.soard.2023.01.020
73. Challa DN, Kvrgic Z, Cheville AL, et al. Patient-provider discordance between global assessments of disease activity in rheumatoid arthritis: a comprehensive clinical evaluation. *Arthritis Res Ther*. 2017;19(1):212. doi:https://dx.doi.org/10.1186/s13075-017-1419-5
74. Chana P, Smith JG, Karamat A, Simpson A, Renton T. Catastrophising, pain self-efficacy and acceptance in patients with Burning Mouth Syndrome. *J Oral Rehabil*. 2021;48(4):458-468. doi:https://dx.doi.org/10.1111/joor.13136
75. Chang YP. Factors associated with prescription opioid misuse in adults aged 50 or older. *Nurs Outlook*. 2018;66(2):112-120. doi:https://dx.doi.org/10.1016/j.outlook.2017.10.007
76. Cheatle MD, Giordano NA, Themelis K, Tang NKY. Suicidal thoughts and behaviors in patients with chronic pain, with and without co-occurring opioid use disorder. *Pain Med*. 2023;24(8):941-948. doi:https://dx.doi.org/10.1093/pm/pnad043
77. Chen JA, Anderson ML, Cherkin DC, et al. Moderators and Nonspecific Predictors of Treatment Benefits in a Randomized Trial of Mindfulness-Based Stress Reduction vs. Cognitive-Behavioral Therapy vs. Usual Care for Chronic Low Back Pain. *J Pain*. 2022;27:27. doi:https://dx.doi.org/10.1016/j.jpain.2022.09.014
78. Cheng AL, Bradley EC, Brady BK, et al. The Influence of Race, Sex, and Social Disadvantage on Self-reported Health in Patients Presenting With Chronic Musculoskeletal Pain. *Am J Phys Med Rehabil*. 2022;101(3):211-216. doi:https://dx.doi.org/10.1097/PHM.0000000000001774
79. Cheng AL, Brady BK, Bradley EC, et al. Opioid use and social disadvantage in patients with chronic musculoskeletal pain. *Pm R*. 2022;14(3):309-319. doi:https://dx.doi.org/10.1002/pmrj.12596
80. Cheng ST, Leung CMC, Chan KL, et al. The relationship of self-efficacy to catastrophizing and depressive symptoms in community-dwelling older adults with chronic pain: A moderated mediation model. *PLoS ONE*. 2018;13(9):e0203964. doi:https://dx.doi.org/10.1371/journal.pone.0203964
81. Choi JH, Lee SH, Kim HR, Lee KA. Association of neuropathic-like pain characteristics with clinical and radiographic features in patients with ankylosing spondylitis. *Clin Rheumatol*. 2018;37(11):3077-3086. doi:https://dx.doi.org/10.1007/s10067-018-4125-z
82. Chytas V, Costanza A, Mazzola V, Luthy C, Bondolfi G, Cedraschi C. Demoralization and Suicidal Ideation in Chronic Pain Patients. *Psychol*. 2023;16:611-617. doi:https://dx.doi.org/10.2147/PRBM.S367461
83. Ciaramella A, Poli P. Chronic Low Back Pain: Perception and Coping With Pain in the Presence of Psychiatric Comorbidity. *J Nerv Ment Dis*. 2015;203(8):632-40. doi:https://dx.doi.org/10.1097/NMD.0000000000000340
84. Civelek GM, Kiliç M. RELATION OF FEAR-AVOIDANCE BEHAVIOURS, OBESITY, DEPRESSION, AND DISABILITY IN GERIATRIC WOMEN WITH CHRONIC LOW BACK PAIN. *Turk Geriatri Dergisi*. 2022;25(2):236-245. doi:10.31086/tjgeri.2022.281
85. Clark JR, Nijs J, Yeowell G, Holmes P, Goodwin PC. Trait Sensitivity, Anxiety, and Personality Are Predictive of Central Sensitization Symptoms in Patients with Chronic Low Back Pain. *Pain pract*. 2019;19(8):800-810. doi:https://dx.doi.org/10.1111/papr.12809
86. Clark JR, Yeowell G, Goodwin PC. Trait anxiety and sensory processing profile characteristics in patients with non-specific chronic low back pain and central sensitisation - A pilot observational study. *J Bodywork Mov Ther*. 2018;22(4):909-916. doi:https://dx.doi.org/10.1016/j.jbmt.2017.11.007
87. Cody GR, Wang B, Link AR, Sherman SE. Characteristics of Urban Inpatient Smokers With and Without Chronic

Pain: Foundations for Targeted Cessation Programs. *Subst Use Misuse*. 2019;54(7):1138-1145. doi:https://dx.doi.org/10.1080/10826084.2018.1563186
88. Conway D, Ladlow P, Ferreira J, Mani-Babu S, Bennett AN. Cognitive functional therapy (CFT)-based rehabilitation improves clinical outcomes in UK military personnel with persistent low back pain. *BMJ Mil Health*. 2020;166(5):336-341. doi:https://dx.doi.org/10.1136/jramc-2018-001136
89. Corriger A, Voute M, Lambert C, Pereira B, Pickering G, Consortium O. Ketamine for refractory chronic pain: a 1-year follow-up study. *Pain*. 2022;163(4):690-701. doi:https://dx.doi.org/10.1097/j.pain.0000000000002403
90. Costa MDSS, Gomez RS. Quality of life and functionality in patients suffering from chronic pain, anxiety and depression. *Archives of Psychiatry and Psychotherapy*. 2023;25(1):79-93. doi:10.12740/APP/155142
91. Csupak B, Sommer JL, Jacobsohn E, El-Gabalawy R. A population-based examination of the co-occurrence and functional correlates of chronic pain and generalized anxiety disorder. *J Anxiety Disord*. 2018;56:74-80. doi:https://dx.doi.org/10.1016/j.janxdis.2018.04.005
92. D'Souza RS, Lin G, Oh T, et al. Fibromyalgia Symptom Severity and Psychosocial Outcomes in Fibromyalgia Patients with Hypovitaminosis D: A Prospective Questionnaire Study. *Pain Med*. 2020;21(12):3470-3478. doi:https://dx.doi.org/10.1093/pm/pnz377
93. Da Luz RA, de Deus JM, Conde DM. Quality of life and associated factors in Brazilian women with chronic pelvic pain. *J Pain Res*. 2018;11:1367-1374. doi:https://dx.doi.org/10.2147/JPR.S168402
94. Da Luz RA, de Deus JM, Valadares AL, Conde DM. Evaluation of sexual function in Brazilian women with and without chronic pelvic pain. *J Pain Res*. 2018;11:2761-2767. doi:https://dx.doi.org/10.2147/JPR.S176851
95. Damci A, Schruers KRJ, Leue C, Faber CG, Hoeijmakers JGJ. Anxiety and depression in small fiber neuropathy. *J Peripher Nerv Syst*. 2022;28:28. doi:https://dx.doi.org/10.1111/jns.12514
96. Davis MC, Thummala K, Zautra AJ. Stress-related clinical pain and mood in women with chronic pain: moderating effects of depression and positive mood induction. *Ann Behav Med*. 2014;48(1):61-70. doi:https://dx.doi.org/10.1007/s12160-013-9583-6
97. de Heer EW, Vriezekolk JE, van der Feltz-Cornelis CM. Poor Illness Perceptions Are a Risk Factor for Depressive and Anxious Symptomatology in Fibromyalgia Syndrome: A Longitudinal Cohort Study. *Front Psychiatr*. 2017;8:217. doi:https://dx.doi.org/10.3389/fpsyt.2017.00217
98. De La Rosa JS, Brady BR, Ibrahim MM, et al. Co-occurrence of chronic pain and anxiety/depression symptoms in U.S. adults: prevalence, functional impacts, and opportunities. *Pain*. 2023;21:21. doi:https://dx.doi.org/10.1097/j.pain.0000000000003056
99. de Moraes Vieira EB, de Goes Salvetti M, Damiani LP, de Mattos Pimenta CA. Self-efficacy and fear avoidance beliefs in chronic low back pain patients: coexistence and associated factors. *Pain Manag Nurs*. 2014;15(3):593-602. doi:https://dx.doi.org/10.1016/j.pmn.2013.04.004
100. De Roa P, Paris P, Poindessous JL, Maillet O, Heron A. Subjective Experiences and Sensitivities in Women with Fibromyalgia: A Quantitative and Comparative Study. *Pain Res Manag*. 2018;2018:8269564. doi:https://dx.doi.org/10.1155/2018/8269564
101. Dear BF, Walker J, Karin E, et al. Evaluation of a therapist-guided virtual psychological pain management program when provided as routine care: A prospective pragmatic cohort study. *Pain Med*. 2023;04:04. doi:https://dx.doi.org/10.1093/pm/pnad102
102. Del Pozo-Cruz J, Alfonso-Rosa RM, Castillo-Cuerva A, Sanudo B, Nolan P, Del Pozo-Cruz B. Depression symptoms are associated with key health outcomes in women with fibromyalgia: a cross-sectional study. *Int J Rheum Dis*. 2017;20(7):798-808. doi:https://dx.doi.org/10.1111/1756-185X.12564
103. Depintor JD, Bracher ES, Cabral DM, Eluf-Neto J. Prevalence of chronic spinal pain and identification of associated factors in a sample of the population of Sao Paulo, Brazil: cross-sectional study. *Sao Paulo Med J*. 2016;134(5):375-384. doi:https://dx.doi.org/10.1590/1516-3180.2016.0091310516
104. Doherty EM, Walsh R, Andrews L, McPherson S. Measuring Emotional Intelligence Enhances the Psychological Evaluation of Chronic Pain. *J Clin Psychol Med Settings*. 2017;24(3-4):365-375. doi:https://dx.doi.org/10.1007/s10880-017-9515-x
105. Domenech J, Sanchis-Alfonso V, Lopez L, Espejo B. Influence of kinesiophobia and catastrophizing on pain and disability in anterior knee pain patients. *Knee Surg Sports Traumatol Arthrosc*. 2013;21(7):1562-8. doi:https://dx.doi.org/10.1007/s00167-012-2238-5
106. Dos Santos Proenca J, Baad-Hansen L, do Vale Braido GV, Campi LB, de Godoi Goncalves DA. Clinical features of chronic primary pain in individuals presenting painful temporomandibular disorder and comorbidities. *J Oral Rehabil*. 2023;19:19. doi:https://dx.doi.org/10.1111/joor.13598
107. Dougados M, Logeart I, Szumski A, Coindreau J, Jones H. Evaluation of whether extremely high enthesitis or Bath Ankylosing Spondylitis Disease Activity Index (BASDAI) scores suggest fibromyalgia and confound the anti-TNF response in early non-radiographic axial spondyloarthritis. *Clin Exp Rheumatol*. 2017;35 Suppl 105(3):50-53.
108. Drummond PD, Willox M. Painful effects of auditory startle, forehead cooling and psychological stress in patients with fibromyalgia or rheumatoid arthritis. *J Psychosom Res*. 2013;74(5):378-83.

© 2025 Aaron RV et al. *JAMA Network Open.*

doi:https://dx.doi.org/10.1016/j.jpsychores.2013.01.011
109. Dubois-Mendes SM, Sá KN, Meneses FM, De Andrade DC, Baptista AF. Neuropathic pain in rheumatoid arthritis and its association with Afro-descendant ethnicity: a hierarchical analysis. *Psychology, health & medicine*. 2021;26(3):278-288. doi:10.1080/13548506.2020.1749677
110. Dumolard A, Lefaucheur JP, Hodaj E, Liateni Z, Payen JF, Hodaj H. Central Sensitization and Small-fiber Neuropathy Are Associated in Patients With Fibromyalgia. *Clin J Pain*. 2023;39(1):8-14. doi:https://dx.doi.org/10.1097/AJP.0000000000001085
111. Duran J, Zitko P, Barrios P, Margozzini P. Chronic Musculoskeletal Pain and Chronic Widespread Pain in Chile: Prevalence Study Performed as Part of the National Health Survey. *J*. 2021;27(6S):S294-S300. doi:https://dx.doi.org/10.1097/RHU.0000000000001642
112. Ecija C, Catala P, Lopez-Gomez I, Bedmar D, Penacoba C. What Does the Psychological Flexibility Model Contribute to the Relationship Between Depression and Disability in Chronic Pain? The Role of Cognitive Fusion and Pain Acceptance. *Clin Nurs Res*. 2022;31(2):217-229. doi:https://dx.doi.org/10.1177/10547738211034307
113. Edit V, Eva S, Maria K, et al. Psychosocial, educational, and somatic factors in chronic nonspecific low back pain. *Rheumatology International*. 2013;33(3):587-592. doi:10.1007/s00296-012-2398-0
114. Elrashidi MY, Philpot LM, Ramar P, Leasure WB, Ebbert JO. Depression and Anxiety Among Patients on Chronic Opioid Therapy. *Health serv*. 2018;5:2333392818771243. doi:https://dx.doi.org/10.1177/2333392818771243
115. Emery PC, Wilson KG, Kowal J. Major depressive disorder and sleep disturbance in patients with chronic pain. *Pain Res Manag*. 2014;19(1):35-41.
116. Englbrecht M, Alten R, Aringer M, et al. New insights into the prevalence of depressive symptoms and depression in rheumatoid arthritis - Implications from the prospective multicenter VADERA II study. *PLoS ONE*. 2019;14(5):e0217412. doi:https://dx.doi.org/10.1371/journal.pone.0217412
117. Eriksen LE, Terkelsen AJ, Blichfeldt-Eckhardt MR, Sørensen JCH, Meier K. Spinal cord stimulation in severe cases of complex regional pain syndrome: A retrospective cohort study with long-term follow-up. *European Journal of Pain (United Kingdom)*. 2021;25(10):2212-2225. doi:10.1002/ejp.1834
118. Falling C, Stebbings S, Baxter GD, et al. Symptoms of central sensitization in patients with inflammatory bowel diseases: a case-control study examining the role of musculoskeletal pain and psychological factors. *Scand J Pain*. 2021;21(2):283-295. doi:https://dx.doi.org/10.1515/sjpain-2020-0109
119. Farin E. The reciprocal effect of pain catastrophizing and satisfaction with participation in the multidisciplinary treatment of patients with chronic back pain. *Health Qual Life Outcomes*. 2015;13:163. doi:https://dx.doi.org/10.1186/s12955-015-0359-5
120. Farzad M, MacDermid JC, Packham T, Khodabandeh B, Vahedi M, Shafiee E. Factors associated with disability and pain intensity in patients with complex regional pain syndrome. *Disabil Rehabil*. 2021:1-9. doi:https://dx.doi.org/10.1080/09638288.2021.2009045
121. Feingold D, Brill S, Goor-Aryeh I, Delayahu Y, Lev-Ran S. Depression and anxiety among chronic pain patients receiving prescription opioids and medical marijuana. *J Affect Disord*. 2017;218:1-7. doi:https://dx.doi.org/10.1016/j.jad.2017.04.026
122. Feingold D, Brill S, Goor-Aryeh I, Delayahu Y, Lev-Ran S. The association between severity of depression and prescription opioid misuse among chronic pain patients with and without anxiety: A cross-sectional study. *J Affect Disord*. 2018;235:293-302. doi:https://dx.doi.org/10.1016/j.jad.2018.04.058
123. Feingold D, Brill S, Goor-Aryeh I, Delayahu Y, Lev-Ran S. Depression level, not pain severity, is associated with smoked medical marijuana dosage among chronic pain patients. *J Psychosom Res*. 2020;135:110130. doi:https://dx.doi.org/10.1016/j.jpsychores.2020.110130
124. Feingold D, Goor-Aryeh I, Bril S, Delayahu Y, Lev-Ran S. Problematic Use of Prescription Opioids and Medicinal Cannabis Among Patients Suffering from Chronic Pain. *Pain Med*. 2017;18(2):294-306. doi:https://dx.doi.org/10.1093/pm/pnw134
125. Feliu-Soler A, Reche-Camba E, Borras X, et al. Psychometric Properties of the Cognitive Emotion Regulation Questionnaire (CERQ) in Patients with Fibromyalgia Syndrome. *Front Psychol*. 2017;8:2075. doi:https://dx.doi.org/10.3389/fpsyg.2017.02075
126. Fiegl S, Lahmann C, O'Rourke T, Probst T, Pieh C. Depression According to ICD-10 Clinical Interview vs. Depression According to the Epidemiologic Studies Depression Scale to Predict Pain Therapy Outcomes. *Front Psychol*. 2019;10:1862. doi:https://dx.doi.org/10.3389/fpsyg.2019.01862
127. Filippon APM, Bassani DG, de Aguiar RW, Ceitlin LHF. Association between childhood trauma and loss of functionality in adult women with fibromyalgia. *Trends in Psychiatry and Psychotherapy*. 2013;35(1):46-54. doi:10.1590/S2237-60892013000100006
128. Finn E, Morrison TG, McGuire BE. Correlates of Sexual Functioning and Relationship Satisfaction Among Men and Women Experiencing Chronic Pain. *Pain Med*. 2018;19(5):942-954. doi:https://dx.doi.org/10.1093/pm/pnx056
129. Fischer-Jbali LR, Montoro CI, Montoya P, Halder W, Duschek S. Central nervous activity during a dot probe task with facial expressions in fibromyalgia. *Biol Psychol*. 2022;172:108361.

© 2025 Aaron RV et al. *JAMA Network Open.*

doi:https://dx.doi.org/10.1016/j.biopsycho.2022.108361
130. Flores-Curiel WA, Carrazco-Pena KB, Mendoza-Cano O. Frequency of Spinal Segmental Sensitization Syndrome and Associated Social Determinants of Health. *Arch Phys Med Rehabil*. 2023;24:24. doi:https://dx.doi.org/10.1016/j.apmr.2023.04.025
131. Foti R, Amato G, Dal Bosco Y, et al. Telemedicine in the Management of Patients with Rheumatic Disease during COVID-19 Pandemic: Incidence of Psychiatric Disorders and Fibromyalgia in Patients with Rheumatoid Arthritis and Psoriatic Arthritis. *Int J Environ Res Public Health*. 2022;19(6):08. doi:https://dx.doi.org/10.3390/ijerph19063161
132. Frede N, Hiestand S, Endres D, et al. Burden of disease and impact on quality of life in chronic back pain - a comparative cross-sectional study of 150 axial spondyloarthritis and 150 orthopedic back pain patients. *Front Med (Lausanne)*. 2023;10:1221087. doi:https://dx.doi.org/10.3389/fmed.2023.1221087
133. Freo U, Furnari M, Ambrosio F, Navalesi P. Efficacy and tolerability of tapentadol for the treatment of chronic low back pain in elderly patients. *Aging Clin Exp Res*. 2021;33(4):973-982. doi:https://dx.doi.org/10.1007/s40520-020-01586-0
134. Garaigordobil M, Govillard L. Fibromyalgia: effect of a cognitive behavioral treatment with and without biofeedback on psychopathological symptoms. *Behavioral Psychology / Psicología Conductual: Revista Internacional Clínica y de la Salud*. 2016;24(3):439-457.
135. Garbi Mde O, Hortense P, Gomez RR, da Silva Tde C, Castanho AC, Sousa FA. Pain intensity, disability and depression in individuals with chronic back pain. *Rev Lat Am Enfermagem*. 2014;22(4):569-75.
136. Garcia Carrasco M, Mendoza Pinto C, Lopez Colombo A, et al. Irritable bowel syndrome-type symptoms in female patients with mild systemic lupus erythematosus: frequency, related factors and quality of life. *Neurogastroenterol Motil*. 2013;25(12):958-66. doi:https://dx.doi.org/10.1111/nmo.12230
137. Garnaes KK, Morkved S, Tonne T, Furan L, Vasseljen O, Johannessen HH. Mental health among patients with chronic musculoskeletal pain and its relation to number of pain sites and pain intensity, a cross-sectional study among primary health care patients. *BMC Musculoskelet Disord*. 2022;23(1):1115. doi:https://dx.doi.org/10.1186/s12891-022-06051-9
138. Gavilan-Carrera B, Delgado-Fernandez M, Sierra-Nieto E, et al. Sedentary time is associated with depressive symptoms and state anxiety in women with fibromyalgia. Could physical activity and fitness modify this association? The al-Andalus project. *Disabil Rehabil*. 2022:1-9. doi:https://dx.doi.org/10.1080/09638288.2022.2122602
139. Gebauer S, Salas J, Scherrer JF, Burge S, Schneider FD. Disability Benefits and Change in Prescription Opioid Dose. *Popul Health Manag*. 2019;22(6):503-510. doi:https://dx.doi.org/10.1089/pop.2018.0210
140. Gelonch O, Garolera M, Valls J, et al. The effect of depressive symptoms on cognition in patients with fibromyalgia. *PLoS ONE*. 2018;13(7):e0200057. doi:https://dx.doi.org/10.1371/journal.pone.0200057
141. Gelonch O, Garolera M, Valls J, Rossello L, Pifarre J. Cognitive complaints in women with fibromyalgia: Are they due to depression or to objective cognitive dysfunction? *J Clin Exp Neuropsychol*. 2017;39(10):1013-1025. doi:https://dx.doi.org/10.1080/13803395.2017.1301391
142. Gentili C, Rickardsson J, Zetterqvist V, Simons LE, Lekander M, Wicksell RK. Psychological Flexibility as a Resilience Factor in Individuals With Chronic Pain. *Front Psychol*. 2019;10:2016. doi:https://dx.doi.org/10.3389/fpsyg.2019.02016
143. Gerdle B, Åkerblom S, Jansen G, et al. Who benefits from multimodal rehabilitation – an exploration of pain, psychological distress, and life impacts in over 35,000 chronic pain patients identified in the swedish quality registry for pain rehabilitation. *Journal of Pain Research*. 2019;12:891-908. doi:10.2147/JPR.S190003
144. Gerra MC, Gonzalez-Villar A, Arendt-Nielsen L, et al. A family-based study to identify genetic biomarkers of fibromyalgia: consideration of patients' subgroups. *Clin Exp Rheumatol*. 2021;39 Suppl 130(3):144-152. doi:https://dx.doi.org/10.55563/clinexprheumatol/bq7x9n
145. Gisev N, Nielsen S, Campbell G, et al. Antidepressant Use Among People Prescribed Opioids for Chronic Noncancer Pain. *Pain Med*. 2019;20(12):2450-2458. doi:https://dx.doi.org/10.1093/pm/pnz009
146. Giummarra MJ, Tardif H, Blanchard M, Tonkin A, Arnold CA. Hypertension prevalence in patients attending tertiary pain management services, a registry-based Australian cohort study. *PLoS ONE*. 2020;15(1):e0228173. doi:https://dx.doi.org/10.1371/journal.pone.0228173
147. Goesling J, Brummett CM, Meraj TS, Moser SE, Hassett AL, Ditre JW. Associations Between Pain, Current Tobacco Smoking, Depression, and Fibromyalgia Status Among Treatment-Seeking Chronic Pain Patients. *Pain Med*. 2015;16(7):1433-42. doi:https://dx.doi.org/10.1111/pme.12747
148. Goesling J, Henry MJ, Moser SE, et al. Symptoms of Depression Are Associated With Opioid Use Regardless of Pain Severity and Physical Functioning Among Treatment-Seeking Patients With Chronic Pain. *J Pain*. 2015;16(9):844-51. doi:https://dx.doi.org/10.1016/j.jpain.2015.05.010
149. Goesling J, Moser SE, Lin LA, Hassett AL, Wasserman RA, Brummett CM. Discrepancies Between Perceived Benefit of Opioids and Self-Reported Patient Outcomes. *Pain Med*. 2018;19(2):297-306. doi:https://dx.doi.org/10.1093/pm/pnw263
150. Gomes LA, Rodrigues AM, Branco JC, Canhao H, Cruz EB. Clinical courses, impact and prognostic indicators for a persistent course of low back pain: Results from a population-based cohort study. *PLoS ONE*. 2023;18(3):e0265104.

© 2025 Aaron RV et al. *JAMA Network Open.*

doi:https://dx.doi.org/10.1371/journal.pone.0265104
151. Gorpynchenko I, Nurimanov K, Poroshina T, Savchenko V, Drannik G, Shulyak A. Antibiotic therapy effectiveness as an outcome predictor of complex treatment in chronic prostatitis/chronic pelvic pain syndrome. *Cent*. 2021;74(2):241-248. doi:https://dx.doi.org/10.5173/ceju.2021.0195.R2
152. Gota CE, Kaouk S, Wilke WS. The impact of depressive and bipolar symptoms on socioeconomic status, core symptoms, function and severity of fibromyalgia. *Int J Rheum Dis*. 2017;20(3):326-339. doi:https://dx.doi.org/10.1111/1756-185X.12603
153. Govind V, Krapf JM, Mitchell L, et al. Exploring Pain-Related Anxiety and Depression in Female Patients With Provoked Vulvodynia With Associated Overactive Pelvic Floor Muscle Dysfunction. *Sex*. 2020;8(3):517-524. doi:https://dx.doi.org/10.1016/j.esxm.2020.05.009
154. Greenberg J, Bakhshaie J, Lovette BC, Vranceanu AM. Association Between Coping Strategies and Pain-Related Outcomes Among Individuals with Chronic Orofacial Pain. *J Pain Res*. 2022;15:431-442. doi:https://dx.doi.org/10.2147/JPR.S350024
155. Gunduz N, Erzincan E, Polat A. The Relationship of Intimate Partner Violence With Psychiatric Disorders and Severity of Pain Among Female Patients With Fibromyalgia. *Arch*. 2019;34(3):245-252. doi:https://dx.doi.org/10.5606/ArchRheumatol.2019.7090
156. Gunduz N, Polat A, Erzincan E, Turan H, Sade I, Tural U. Psychiatric comorbidity and childhood trauma in fibromyalgia syndrome. *Turk J Phys Med Rehabil*. 2018;64(2):91-99. doi:https://dx.doi.org/10.5606/tftrd.2018.1470
157. Gunduz N, Usen A, Aydin Atar E. The Impact of Perceived Social Support on Anxiety, Depression and Severity of Pain and Burnout Among Turkish Females With Fibromyalgia. *Arch*. 2019;34(2):186-195. doi:https://dx.doi.org/10.5606/ArchRheumatol.2019.7018
158. Gupta P, Singh V, Sethi S, Kumar A. A Comparative Pilot Study to Evaluate the Adjunctive Role of Levosulpride with Trigger Point Injection Therapy in the Management of Myofascial Pain Syndrome of Orofacial Region. 2014;1(4):599-602.
159. Gupta P, Singh V, Sethi S, Kumar A. A Comparative Study of Trigger Point Therapy with Local Anaesthetic (0.5 % Bupivacaine) Versus Combined Trigger Point Injection Therapy and Levosulpiride in the Management of Myofascial Pain Syndrome in the Orofacial Region. *J*. 2016;15(3):376-383.
160. Hallstam A, Lofgren M, Benson L, Svensen C, Stalnacke BM. Assessment and treatment at a pain clinic: A one-year follow-up of patients with chronic pain. *Scand J Pain*. 2017;17:233-242. doi:https://dx.doi.org/10.1016/j.sjpain.2016.08.004
161. Hamdi W, Souissi MA, Lassoued Ferjani H, Maatallah K, Cherif I, Kaffel D. Sleep disturbances in chronic rheumatic diseases: Is disease activity the major determinant factor? *Tunis Med*. 2021;99(8):890-897.
162. Han SB, Lee SH, Ha IH, Kim EJ. Association between severity of depressive symptoms and chronic knee pain in Korean adults aged over 50 years: a cross-sectional study using nationally representative data. *BMJ Open*. 2019;9(12):e032451. doi:https://dx.doi.org/10.1136/bmjopen-2019-032451
163. Hansdorfer-Korzon R, Chojnacka-Szawłowska G, Landowski J, et al. Relationships of anxiety and depressive symptoms with pain perception in post-mastectomy women. An intragroup analysis. *Revista de Psiquiatria Clinica*. 2016;43(4):74-78. doi:10.1590/0101-60830000000088
164. Hardy A, Sandiford MH, Menigaux C, Bauer T, Klouche S, Hardy P. Pain catastrophizing and pre-operative psychological state are predictive of chronic pain after joint arthroplasty of the hip, knee or shoulder: results of a prospective, comparative study at one year follow-up. *Int Orthop*. 2022;46(11):2461-2469. doi:https://dx.doi.org/10.1007/s00264-022-05542-7
165. Harrison L, Wilson S, Heron J, Stannard C, Munafo MR. Exploring the associations shared by mood, pain-related attention and pain outcomes related to sleep disturbance in a chronic pain sample. *Psychol Health*. 2016;31(5):565-77. doi:https://dx.doi.org/10.1080/08870446.2015.1124106
166. Hasuo H, Sakuma H, Fukunaga M. Alexithymia in Family Caregivers of Advanced Cancer Patients Is Associated with High Personalized Pain Goal Scores: A Pilot Study. *J Palliat Med*. 2020;23(7):930-936. doi:https://dx.doi.org/10.1089/jpm.2019.0257
167. Hauser W, Bialas P, Welsch K, Wolfe F. Construct validity and clinical utility of current research criteria of DSM-5 somatic symptom disorder diagnosis in patients with fibromyalgia syndrome. *J Psychosom Res*. 2015;78(6):546-52. doi:https://dx.doi.org/10.1016/j.jpsychores.2015.03.151
168. Hauser W, Brahler E, Wolfe F, Henningsen P. Patient Health Questionnaire 15 as a generic measure of severity in fibromyalgia syndrome: surveys with patients of three different settings. *J Psychosom Res*. 2014;76(4):307-11. doi:https://dx.doi.org/10.1016/j.jpsychores.2014.01.009
169. Hauser W, Hoffmann EM, Wolfe F, et al. Self-reported childhood maltreatment, lifelong traumatic events and mental disorders in fibromyalgia syndrome: a comparison of US and German outpatients. *Clin Exp Rheumatol*. 2015;33(1 Suppl 88):S86-92.
170. Hoban B, Larance B, Gisev N, et al. The use of paracetamol (acetaminophen) among a community sample of people with chronic non-cancer pain prescribed opioids. *Int J Clin Pract*. 2015;69(11):1366-76.

© 2025 Aaron RV et al. *JAMA Network Open.*

doi:https://dx.doi.org/10.1111/ijcp.12716
171. Hochman JR, Davis AM, Elkayam J, Gagliese L, Hawker GA. Neuropathic pain symptoms on the modified painDETECT correlate with signs of central sensitization in knee osteoarthritis. *Osteoarthritis Cartilage*. 2013;21(9):1236-42. doi:https://dx.doi.org/10.1016/j.joca.2013.06.023
172. Hogestol IK, Chahal-Kummen M, Eribe I, et al. Chronic Abdominal Pain and Symptoms 5 Years After Gastric Bypass for Morbid Obesity. *Obes Surg*. 2017;27(6):1438-1445. doi:https://dx.doi.org/10.1007/s11695-016-2499-z
173. Holloway BM, Santoro MS, Cronan TA. Smoking, depression, & stress: predictors of fibromyalgia health status. *Psychol Health Med*. 2017;22(1):87-93.
174. Honda H, Ashizawa R, Kiriyama K, Take K, Yoshimoto Y. Depression Is Associated with Chronic Pain in Disabled Older Adults. *Exp Aging Res*. 2022;48(3):287-294. doi:https://dx.doi.org/10.1080/0361073X.2021.1979346
175. Hong S, Petrov V, Rana H. Under-treatment of depression at an academic chronic pain management clinic. *Journal of Pain Management*. 2018;11(4):411-416.
176. Hudak J, Bernat EM, Fix ST, Prince KC, Froeliger B, Garland EL. Neurophysiological Deficits During Reappraisal of Negative Emotional Stimuli in Opioid Misuse. *Biol Psychiatry*. 2022;91(12):1070-1078. doi:https://dx.doi.org/10.1016/j.biopsych.2022.01.019
177. Hughes JM, Seemann EA, George JM, Willis KD. The Effects of Pre-treatment Depressive Symptoms on Quality of Life Across Cognitive Behavioral Therapy for Chronic Pain. *J Clin Psychol Med Settings*. 2019;26(1):97-105. doi:https://dx.doi.org/10.1007/s10880-018-9568-5
178. Iannuccelli C, Lucchino B, Gioia C, et al. Mental health and well-being during the COVID-19 pandemic: stress vulnerability, resilience and mood disturbances in fibromyalgia and rheumatoid arthritis. *Clin Exp Rheumatol*. 2021;39 Suppl 130(3):153-160. doi:https://dx.doi.org/10.55563/clinexprheumatol/4nb0ku
179. Inanc N, Yilmaz-Oner S, Can M, Sokka T, Direskeneli H. The role of depression, anxiety, fatigue, and fibromyalgia on the evaluation of the remission status in patients with rheumatoid arthritis. *J Rheumatol*. 2014;41(9):1755-60. doi:https://dx.doi.org/10.3899/jrheum.131171
180. Ingegnoli F, Schioppo T, Ubiali T, et al. Patient Perception of Depressive Symptoms in Rheumatic Diseases: A Cross-sectional Survey. *J*. 2022;28(1):e18-e22. doi:https://dx.doi.org/10.1097/RHU.0000000000001564
181. Islam ST, Descallar J, Martens D, Hassett G, Gibson KA. Screening for anxiety in patients with inflammatory arthritis using the multidimensional health assessment questionnaire (MDHAQ). *The Journal of rheumatology*. 2023;doi:10.3899/jrheum.2022-1261
182. Jain A, Bhadauria D. Evaluation of efficacy of fluoxetine in the management of major depression and arthritis in patients of Rheumatoid Arthritis. *Indian Journal of Rheumatology*. 2013;8(4):165-169. doi:10.1016/j.injr.2013.08.001
183. Jaiswal A, Scherrer JF, Salas J, van den Berk-Clark C, Fernando S, Herndon CM. Differences in the Association between Depression and Opioid Misuse in Chronic Low Back Pain versus Chronic Pain at Other Locations. *Healthcare (Basel)*. 2016;4(2):16. doi:https://dx.doi.org/10.3390/healthcare4020034
184. Janela D, Costa F, Molinos M, et al. Asynchronous and Tailored Digital Rehabilitation of Chronic Shoulder Pain: A Prospective Longitudinal Cohort Study. *J Pain Res*. 2022;15:53-66. doi:https://dx.doi.org/10.2147/JPR.S343308
185. Jaremo P, Arman M, Gerdle B, Gottberg K. Facing Invalidation: A Further Challenge when Living with Chronic Widespread Pain. *J Rehabil Med*. 2022;54:jrm00280. doi:https://dx.doi.org/10.2340/jrm.v54.67
186. Jaremo P, Arman M, Gerdle B, Larsson B, Gottberg K. Illness beliefs among patients with chronic widespread pain - associations with self-reported health status, anxiety and depressive symptoms and impact of pain. *BMC Psychol*. 2017;5(1):24. doi:https://dx.doi.org/10.1186/s40359-017-0192-1
187. Jedel E, Elfström ML, Hägglin C. Health-related quality of life in burning mouth syndrome - a case-control study. *Scandinavian Journal of Pain*. 2020;20(4):829-836. doi:10.1515/sjpain-2020-0047
188. Jegan NR, Brugger M, Viniol A, et al. Psychological risk and protective factors for disability in chronic low back pain - a longitudinal analysis in primary care. *BMC Musculoskelet Disord*. 2017;18(1):114. doi:https://dx.doi.org/10.1186/s12891-017-1482-8
189. Jensen HI, Plesner K, Kvorning N, Krogh BL, Kimper-Karl A. Associations between demographics and health-related quality of life for chronic non-malignant pain patients treated at a multidisciplinary pain centre: a cohort study. *Int J Qual Health Care*. 2016;28(1):86-91. doi:https://dx.doi.org/10.1093/intqhc/mzv108
190. Jiao J, Cheng Z, Wang W, Zhao Y, Jiang Q. Demographic Characteristics and Clinical Features of Fibromyalgia in China: A Cross-Sectional Study. *Rheumatol*. 2021;8(2):817-831. doi:https://dx.doi.org/10.1007/s40744-021-00303-1
191. Jimenez-Rodriguez I, Garcia-Leiva JM, Jimenez-Rodriguez BM, Condes-Moreno E, Rico-Villademoros F, Calandre EP. Suicidal ideation and the risk of suicide in patients with fibromyalgia: a comparison with non-pain controls and patients suffering from low-back pain. 2014;1:625-30.
192. Jin W, Liu Z, Zhang Y, Che Z, Gao M. The Effect of Individual Musculoskeletal Conditions on Depression: Updated Insights From an Irish Longitudinal Study on Aging. *Front Med (Lausanne)*. 2021;8:697649. doi:https://dx.doi.org/10.3389/fmed.2021.697649
193. Jindal R, Rudol G, Okafor B, Rambani R. Role of psychological distress screening in predicting the outcomes of epidural steroid injection in chronic low back pain. *J*. 2021;19:26-33. doi:https://dx.doi.org/10.1016/j.jcot.2021.04.027

© 2025 Aaron RV et al. *JAMA Network Open.*

194. John H, Lim YH, Hong SJ, et al. Impact of coronavirus disease 2019 on patients with chronic pain: multicenter study in Korea. *Korean J Pain*. 2022;35(2):209-223. doi:https://dx.doi.org/10.3344/kjp.2022.35.2.209
195. Jouini G, Choiniere M, Martin E, et al. Pharmacotherapeutic management of chronic noncancer pain in primary care: lessons for pharmacists. 2014;1:163-73.
196. Joyce C, Roseen EJ, Keysor JJ, Gross KD, Culpepper L, Saper RB. Can Yoga or Physical Therapy for Chronic Low Back Pain Improve Depression and Anxiety Among Adults From a Racially Diverse, Low-Income Community? A Secondary Analysis of a Randomized Controlled Trial. *Arch Phys Med Rehabil*. 2021;102(6):1049-1058. doi:https://dx.doi.org/10.1016/j.apmr.2021.01.072
197. Kadimpati S, Zale EL, Hooten MW, Ditre JW, Warner DO. Associations between Neuroticism and Depression in Relation to Catastrophizing and Pain-Related Anxiety in Chronic Pain Patients. *PLoS ONE*. 2015;10(4):e0126351. doi:https://dx.doi.org/10.1371/journal.pone.0126351
198. Kallusky N, Assel C, Grosbach M, et al. Development and Interrelation of Pain, Depression, and Anxiety in Music Students: Does Successful Treatment of Pain Have an Impact on Mental Health? *Med Probl Perform Art*. 2023;38(1):43-55. doi:https://dx.doi.org/10.21091/mppa.2023.1006
199. Karafin MS, Singavi A, Hussain J, et al. Predictive factors of daily opioid use and quality of life in adults with sickle cell disease. *Hematol*. 2018;23(10):856-863. doi:https://dx.doi.org/10.1080/10245332.2018.1479997
200. Karas H, Cetingok H, Iliser R, Carpar E, Kaser M. Childhood and adult attention deficit hyperactivity disorder symptoms in fibromyalgia: associations with depression, anxiety and disease impact. *Int*. 2020;24(3):257-263. doi:https://dx.doi.org/10.1080/13651501.2020.1764585
201. Kazemi H, Ghassemi S, Feres SM, Amini A, Kolivand PH, Doroudi T. Anxiety and depression in patients with amputated limbs suffering from phantom pain: A comparative study with non-phantom chronic pain. *International Journal of Preventive Medicine*. 2013;4(2):218-225.
202. Kec D, Rajdova A, Raputova J, et al. Risk factors for depression and anxiety in painful and painless diabetic polyneuropathy: A multicentre observational cross-sectional study. *Eur J Pain*. 2022;26(2):370-389. doi:https://dx.doi.org/10.1002/ejp.1865
203. Kha TV, Stenager E, Hoang H, et al. Preliminary validity and test-retest reliability of two depression questionnaires compared with a diagnostic interview in 99 patients with chronic pain seeking specialist pain treatment. *Scand J Pain*. 2020;20(4):717-726. doi:https://dx.doi.org/10.1515/sjpain-2020-0042
204. Khazen O, Rosoklija G, Custozzo A, et al. Correlation Between Aspects of Perceived Patient Loneliness and Spinal Cord Stimulation Outcomes. *Neuromodulation*. 2021;24(1):150-155. doi:https://dx.doi.org/10.1111/ner.13299
205. Kibune Nagasako C, Garcia Montes C, Silva Lorena SL, Mesquita MA. Irritable bowel syndrome subtypes: Clinical and psychological features, body mass index and comorbidities. *Rev Esp Enferm Dig*. 2016;108(2):59-64.
206. Kim KH, Lee CK, Kim SH, et al. Prevalence of chronic post-thoracotomy pain in patients with traumatic multiple rib fractures in South Korea: a cross-sectional study. *Sci*. 2021;11(1):2615. doi:https://dx.doi.org/10.1038/s41598-021-82273-6
207. Knaster P, Estlander AM, Karlsson H, Kaprio J, Kalso E. Diagnosing Depression in Chronic Pain Patients: DSM-IV Major Depressive Disorder vs. Beck Depression Inventory (BDI). *PLoS ONE*. 2016;11(3):e0151982. doi:https://dx.doi.org/10.1371/journal.pone.0151982
208. Koh JS, Ko HJ, Wang SM, et al. The impact of depression and somatic symptoms on treatment outcomes in patients with chronic prostatitis/chronic pelvic pain syndrome: a preliminary study in a naturalistic treatment setting. *Int J Clin Pract*. 2014;68(4):478-85. doi:https://dx.doi.org/10.1111/ijcp.12340
209. Koh JS, Ko HJ, Wang SM, et al. Depression and somatic symptoms may influence on chronic prostatitis/chronic pelvic pain syndrome: a preliminary study. 2014;1(4):495-8.
210. Kosson D, Malec-Milewska M, Galazkowski R, Rzonca P. Analysis of Anxiety, Depression and Aggression in Patients Attending Pain Clinics. *Int J Environ Res Public Health*. 2018;15(12):18. doi:https://dx.doi.org/10.3390/ijerph15122898
211. Kroenke K, Stump TE, Chen CX, et al. Minimally important differences and severity thresholds are estimated for the PROMIS depression scales from three randomized clinical trials. *J Affect Disord*. 2020;266:100-108. doi:https://dx.doi.org/10.1016/j.jad.2020.01.101
212. Kroenke K, Stump TE, Kean J, et al. Diagnostic operating characteristics of PROMIS scales in screening for depression. *J Psychosom Res*. 2021;147:110532. doi:https://dx.doi.org/10.1016/j.jpsychores.2021.110532
213. Lamerato L, Shah V, Abraham L, et al. Impact of Electronic Chronic Pain Questions on patient-reported outcomes and healthcare utilization, and attitudes toward eCPQ use among patients and physicians: prospective pragmatic study in a US general practice setting. *Frontiers in Medicine*. 2023;10doi:10.3389/fmed.2023.933975
214. Landefeld JC, Miaskowski C, Tieu L, et al. Characteristics and Factors Associated With Pain in Older Homeless Individuals: Results From the Health Outcomes in People Experiencing Homelessness in Older Middle Age (HOPE HOME) Study. *J Pain*. 2017;18(9):1036-1045. doi:https://dx.doi.org/10.1016/j.jpain.2017.03.011
215. Larance B, Campbell G, Moore T, et al. Concerns and Help-Seeking Among Patients Using Opioids for Management of Chronic Noncancer Pain. *Pain Med*. 2019;20(4):758-769. doi:https://dx.doi.org/10.1093/pm/pny078

© 2025 Aaron RV et al. *JAMA Network Open.*

216. Laurent B, Vicaut E, Leplege A, Bloch K, Leutenegger E. Prevalence and impact on quality of life of post-herpetic neuralgia in French medical centers specialized in chronic pain management: the ZOCAD study. *Med Mal Infect*. 2014;44(11-12):515-24. doi:https://dx.doi.org/10.1016/j.medmal.2014.10.004
217. Laurent Q, Michel-Cherqui M, Szekely B, et al. Prevalence, Characteristics and Preoperative Predictors of Chronic Pain After Double-Lung Transplantation: A Prospective Cohort Study. *J Cardiothorac Vasc Anesth*. 2022;36(2):500-509. doi:https://dx.doi.org/10.1053/j.jvca.2021.07.041
218. Lee DH, Noh EC, Kim YC, et al. Risk Factors for Suicidal Ideation among Patients with Complex Regional Pain Syndrome. 2014;1(1):32-8.
219. Lee SJ, Koussa M, Gelberg L, Heinzerling K, Young SD. Somatization, mental health and pain catastrophizing factors associated with risk of opioid misuse among patients with chronic non-cancer pain. *Journal of Substance Use*. 2020;25(4):357-362. doi:10.1080/14659891.2019.1704079
220. Leon-Suarez P, Rua-Figueroa I, Gonzalez Martin J, et al. Depression and anxiety in systemic lupus erythematosus: A case-control study on prevalence and associated factors in a single-center cohort. *Lupus*. 2023;32(7):827-832. doi:https://dx.doi.org/10.1177/09612033231173510
221. Lerman SF, Rudich Z, Brill S, Shalev H, Shahar G. Longitudinal associations between depression, anxiety, pain, and pain-related disability in chronic pain patients. *Psychosom Med*. 2015;77(3):333-41. doi:https://dx.doi.org/10.1097/PSY.0000000000000158
222. Lin TC, Ger LP, Pergolizzi JV, Jr., Raffa RB, Wang JO, Ho ST. Long-term use of opioids in 210 officially registered patients with chronic noncancer pain in Taiwan: A cross-sectional study. *J Formos Med Assoc*. 2017;116(4):257-265. doi:https://dx.doi.org/10.1016/j.jfma.2016.10.015
223. Lindemann C, Holzl A, Bohle S, Zippelius T, Strube P. How Does Anxiety and Depression Affect the Outcome after Periradicular Infiltration Therapy?-A Retrospective Analysis of Patients Undergoing CT-Guided Single-Level Nerve Root Infiltration Due to Chronic Monoradicular Pain. *Diagnostics (Basel)*. 2023;13(18):08. doi:https://dx.doi.org/10.3390/diagnostics13182882
224. Liu M, McCurry SM, Belza B, et al. Effects of Pain, Insomnia, and Depression on Psychoactive Medication Supply in Older Adults With Osteoarthritis. *Med Care*. 2018;56(12):1024-1031. doi:https://dx.doi.org/10.1097/MLR.0000000000000982
225. Liu M, McCurry SM, Belza B, et al. Effects of Osteoarthritis Pain and Concurrent Insomnia and Depression on Health Care Use in a Primary Care Population of Older Adults. *Arthritis Care Res (Hoboken)*. 2019;71(6):748-757. doi:https://dx.doi.org/10.1002/acr.23695
226. Longo UG, De Salvatore S, Greco A, et al. Influence of Depression and Sleep Quality on Postoperative Outcomes after Total Hip Arthroplasty: A Prospective Study. *Journal of Clinical Medicine*. 2022;11(13)doi:10.3390/jcm11133845
227. Lopez MN, Pierce RS, Gardner RD, Hanson RW. Standardized Beck Depression Inventory-II scores for male veterans coping with chronic pain. *Psychol Serv*. 2013;10(2):257-63. doi:https://dx.doi.org/10.1037/a0027920
228. Luciano JV, Barrada JR, Aguado J, Osma J, Garcia-Campayo J. Bifactor analysis and construct validity of the HADS: a cross-sectional and longitudinal study in fibromyalgia patients. *Psychol Assess*. 2014;26(2):395-406. doi:https://dx.doi.org/10.1037/a0035284
229. Luque-Reca O, Gillanders D, Catala P, Peñacoba C. Psychometric properties of the cognitive fusion questionnaire in females with fibromyalgia. *Current Psychology: A Journal for Diverse Perspectives on Diverse Psychological Issues*. 2021;doi:10.1007/s12144-021-02214-4
230. Mahendru K, Gupta N, Soneja M, et al. Need for Palliative Care in Patient with Rheumatoid Arthritis: A Cross-sectional Observational Study. *Indian J Palliat Care*. 2021;27(2):275-280. doi:10.25259/ijpc_395_20
231. Mahlich J, Schaede U, Sruamsiri R. Shared Decision-Making and Patient Satisfaction in Japanese Rheumatoid Arthritis Patients: A New "Preference Fit" Framework for Treatment Assessment. *Rheumatol*. 2019;6(2):269-283. doi:https://dx.doi.org/10.1007/s40744-019-0156-4
232. Mameli S, Pisanu GM, Sardo S, et al. Oxytocin nasal spray in fibromyalgic patients. *Rheumatol Int*. 2014;34(8):1047-52. doi:https://dx.doi.org/10.1007/s00296-014-2953-y
233. Mann EG, Harrison MB, LeFort S, VanDenKerkhof EG. What Are the Barriers and Facilitators for the Self-Management of Chronic Pain with and without Neuropathic Characteristics? *Pain Manag Nurs*. 2017;18(5):295-308. doi:https://dx.doi.org/10.1016/j.pmn.2017.04.006
234. Mantyselka P, Ali-Sisto T, Kautiainen H, et al. The Association Between Musculoskeletal Pain and Circulating Ornithine: A Population-Based Study. *Pain Med*. 2017;18(6):1145-1151. doi:https://dx.doi.org/10.1093/pm/pnw285
235. Marcum ZA, Zhan HL, Perera S, Moore CG, Fitzgerald GK, Weiner DK. Correlates of gait speed in advanced knee osteoarthritis. *Pain Med*. 2014;15(8):1334-42. doi:https://dx.doi.org/10.1111/pme.12478
236. Marino C, Grimaldi M, Sabatini P, et al. Fibromyalgia and Depression in Women: An 1H-NMR Metabolomic Study. *Metabolites*. 2021;11(7):30. doi:https://dx.doi.org/10.3390/metabo11070429
237. Mattila-Rautiainen S, Venojarvi M, Rautiainen H, Keski-Valkama A. The impact on physical performance, pain and psychological wellbeing of chronic low back pain patients during 12-weeks of equine- facilitated therapy intervention. *Front*. 2023;10:1085768. doi:https://dx.doi.org/10.3389/fvets.2023.1085768

© 2025 Aaron RV et al. *JAMA Network Open.*

238. Mayer TG, Choi Y, Howard KJ, Gatchel RJ. Evaluation of functional restoration outcomes for chronic disabling occupational lower extremity disorders. *J Occup Environ Med*. 2013;55(12):1489-94. doi:https://dx.doi.org/10.1097/JOM.0000000000000013
239. McCracken LM, Buhrman M, Brocki K. COVID-19 and processes of adjustment in people with persistent pain: the role of psychological flexibility. *Scand J Pain*. 2023;23(3):539-545. doi:https://dx.doi.org/10.1515/sjpain-2022-0138
240. McWilliams LA, Sarty G, Kowal J, Wilson KG. A Network Analysis of Depressive Symptoms in Individuals Seeking Treatment for Chronic Pain. *Clin J Pain*. 2017;33(10):899-904. doi:https://dx.doi.org/10.1097/AJP.0000000000000477
241. Mehraban A, Shams J, Moamenzade S, Samimi SM, Rafiee S, Zademohamadi F. The high prevalence of obsessive-compulsive disorder in patients with chronic pain. 2014;1(4):203-8.
242. Merlin JS, Westfall AO, Chamot E, et al. Quantitative Evaluation of an Instrument to Identify Chronic Pain in HIV-Infected Individuals. *AIDS Res Hum Retroviruses*. 2015;31(6):623-7. doi:https://dx.doi.org/10.1089/AID.2014.0362
243. Midbari A, Suzan E, Eisenberg E, et al. Amputation in patients with complex regional pain syndrome: A comparative study between amputees and nonamputees with intractable disease. *Bone and Joint Journal*. 2016;98B(4):548-554. doi:10.1302/0301-620X.98B4.36422
244. Miller-Matero LR, Saulino C, Clark S, Bugenski M, Eshelman A, Eisenstein D. When treating the pain is not enough: a multidisciplinary approach for chronic pelvic pain. *Arch Women Ment Health*. 2016;19(2):349-54. doi:https://dx.doi.org/10.1007/s00737-015-0537-9
245. Moraes EB, Martins Junior FF, Silva LBD, Garcia JBS, Mattos-Pimenta CA. Self-efficacy and fear of pain to movement in chronic low back pain: an intervention developed by nurses. *Rev Gaucha Enferm*. 2021;42:e20200180. doi:https://dx.doi.org/10.1590/1983-1447.2021.20200180
246. Morlà RM, Li T, Castrejon I, Luta G, Pincus T. Multidimensional Health Assessment Questionnaire as an Effective Tool to Screen for Depression in Routine Rheumatology Care. *Arthritis Care and Research*. 2021;73(1):120-129. doi:10.1002/acr.24467
247. Muharam R, Amalia T, Pratama G, et al. Chronic Pelvic Pain in Women with Endometriosis is Associated with Psychiatric Disorder and Quality of Life Deterioration. *Int J Women Health*. 2022;14:131-138. doi:https://dx.doi.org/10.2147/IJWH.S345186
248. Mur Marti T, Llordes Llordes M, Custal Jorda M, Lopez Juan G, Martinez Pardo S. Profile of patients with fibromyalgia being treated in primary care centers in Terrassa, a city in northeastern Spain. *Reumatol*. 2017;13(5):252-257. doi:https://dx.doi.org/10.1016/j.reuma.2016.05.008
249. Murata S, Ono R, Omata J, Endo T, Otani K. Coexistence of Chronic Musculoskeletal Pain and Depressive Symptoms and Their Combined and Individual Effects on Onset of Disability in Older Adults: A Cohort Study. *J Am Med Dir Assoc*. 2019;20(10):1263-1267.e3. doi:https://dx.doi.org/10.1016/j.jamda.2019.04.013
250. Mustonen L, Aho T, Harno H, Sipila R, Meretoja T, Kalso E. What makes surgical nerve injury painful? A 4-year to 9-year follow-up of patients with intercostobrachial nerve resection in women treated for breast cancer. *Pain*. 2019;160(1):246-256. doi:https://dx.doi.org/10.1097/j.pain.0000000000001398
251. Nacak Y, Morawa E, Erim Y. High Rejection Sensitivity in Patients With Somatoform Pain Disorder. *Frontiers in Psychiatry*. 2021;12doi:10.3389/fpsyt.2021.602981
252. Neblett R, Hartzell MM, Mayer TG, Bradford EM, Gatchel RJ. Establishing clinically meaningful severity levels for the Tampa Scale for Kinesiophobia (TSK-13). *Eur J Pain*. 2016;20(5):701-10. doi:https://dx.doi.org/10.1002/ejp.795
253. Neikrug AB, Donaldson G, Iacob E, Williams SL, Hamilton CA, Okifuji A. Activity rhythms and clinical correlates in fibromyalgia. *Pain*. 2017;158(8):1417-1429. doi:https://dx.doi.org/10.1097/j.pain.0000000000000906
254. Nguyen AT, Nguyen THT, Nguyen TTH, et al. Chronic Pain and Associated Factors Related to Depression among Older Patients in Hanoi, Vietnam. *Int J Environ Res Public Health*. 2021;18(17):31. doi:https://dx.doi.org/10.3390/ijerph18179192
255. Norman-Nott N, Wilks CR, Hesam-Shariati N, et al. The No Worries Trial: Efficacy of Online Dialectical Behaviour Therapy Skills Training for Chronic Pain (iDBT-Pain) Using a Single Case Experimental Design. *J Pain*. 2022;23(4):558-576. doi:https://dx.doi.org/10.1016/j.jpain.2021.10.003
256. Offenbaecher M, Kohls N, Toussaint LL, et al. Spiritual needs in patients suffering from fibromyalgia. 2013;1:178547.
257. Ojeda B, Salazar A, Calahorro MJ, et al. Understanding the different relationships between mood and sleep disorders in several groups of non-oncological patients with chronic pain. *Curr Med Res Opin*. 2018;34(4):669-676. doi:https://dx.doi.org/10.1080/03007995.2017.1384372
258. Osorio FL, Carvalho AC, Donadon MF, Moreno AL, Polli-Neto O. Chronic pelvic pain, psychiatric disorders and early emotional traumas: Results of a cross sectional case-control study. *World j*. 2016;6(3):339-44. doi:https://dx.doi.org/10.5498/wjp.v6.i3.339
259. Parisi A, Landicho HL, Hudak J, Leknes S, Froeliger B, Garland EL. Emotional distress and pain catastrophizing predict cue-elicited opioid craving among chronic pain patients on long-term opioid therapy. *Drug Alcohol Depend*. 2022;233:109361. doi:https://dx.doi.org/10.1016/j.drugalcdep.2022.109361
260. Pasin T, Dogruoz Karatekin B, Pasin O. Pain, Anxiety, and Quality of Life of COVID-19 Survivors with Myofascial

© 2025 Aaron RV et al. *JAMA Network Open.*

Pain Syndrome: A cross sectional study. *Pain Manag Nurs*. 2023;24(4):400-405. doi:https://dx.doi.org/10.1016/j.pmn.2022.12.011
261. Peilot B, Andrell P, Gottfries J, Sundler AJ, Mannheimer C. Vulnerability and Resilience in Patients with Chronic Pain in Occupational Healthcare: A Pilot Study with a Patient-Centered Approach. *Pain Res Treat*. 2018;2018:9451313. doi:https://dx.doi.org/10.1155/2018/9451313
262. Penacoba C, Ecija C, Gutierrez L, Catala P. Does Pain Acceptance Contribute to Improved Functionality through Walking in Women with Fibromyalgia? Looking at Depressive Comorbidity. *Int J Environ Res Public Health*. 2023;20(6):12. doi:https://dx.doi.org/10.3390/ijerph20065005
263. Phillips JRA, Hopwood B, Arthur C, Stroud R, Toms AD. The natural history of pain and neuropathic pain after knee replacement: A prospective cohort study of the point prevalence of pain and neuropathic pain to a minimum three-Year follow-up. *Bone and Joint Journal*. 2014;69B(9):1227-1233. doi:10.1302/0301-620X.96B9.33756$2.00
264. Picchianti Diamanti A, Cattaruzza MS, Di Rosa R, et al. Psychological Distress in Patients with Autoimmune Arthritis during the COVID-19 Induced Lockdown in Italy. *Microorganisms*. 2020;8(11)doi:10.3390/microorganisms8111818
265. Pinto SM, Cheung JPY, Samartzis D, et al. Are Morphometric and Biomechanical Characteristics of Lumbar Multifidus Related to Pain Intensity or Disability in People With Chronic Low Back Pain After Considering Psychological Factors or Insomnia? *Front Psychiatr*. 2022;13:809891. doi:https://dx.doi.org/10.3389/fpsyt.2022.809891
266. Plinsinga ML, Coombes BK, Mellor R, Vicenzino B. Individuals with Persistent Greater Trochanteric Pain Syndrome Exhibit Impaired Pain Modulation, as well as Poorer Physical and Psychological Health, Compared with Pain-Free Individuals: A Cross-Sectional Study. *Pain Med*. 2020;21(11):2964-2974. doi:https://dx.doi.org/10.1093/pm/pnaa047
267. Plinsinga ML, Vuvan V, Maclachlan L, et al. Pain-related cognitions and emotional distress are not associated with conditioned pain modulation: an explorative analysis of 1142 participants with acute, subacute, and chronic pain. *Pain*. 2023;164(7):1593-1599. doi:https://dx.doi.org/10.1097/j.pain.0000000000002864
268. Porru D, Anselmo D, Magliano A, et al. Quality of life analysis in bladder pain syndrome/interstitial cystitis: implications for a multimodal integrated treatment. *Minerva Urol Nephrol*. 2023;75(5):634-641. doi:https://dx.doi.org/10.23736/S2724-6051.23.05292-8
269. Poulin PA, Nelli J, Tremblay S, et al. Chronic Pain in the Emergency Department: A Pilot Mixed-Methods Cross-Sectional Study Examining Patient Characteristics and Reasons for Presentations. *Pain Res Manag*. 2016;2016:3092391.
270. Prateepavanich P, Yeephu S, Suttiruksa S, Suthisisang C, Saisavoey N. Demographic and clinical characteristics of thai patients with fibromyalgia syndrome. *Journal of the Medical Association of Thailand*. 2018;101(1):S164-S170.
271. Preti A, Stocchino S, Pinna F, et al. BEEP-Bodily and Emotional Perception of Pain. A Questionnaire to Measure Reaction to Pain in Chronic Pain Disorders. *Front Psychol*. 2019;10:480. doi:https://dx.doi.org/10.3389/fpsyg.2019.00480
272. Priol R, Pasquier G, Putman S, Migaud H, Dartus J, Wattier JM. Trajectory of chronic and neuropathic pain, anxiety and depressive symptoms and pain catastrophizing after total knee replacement. Results of a prospective, single-center study at a mean follow-up of 7.5 years. *Orthop Traumatol Surg Res*. 2023;109(5):103543. doi:https://dx.doi.org/10.1016/j.otsr.2022.103543
273. Proctor SL, Estroff TW, Empting LD, Shearer-Williams S, Hoffmann NG. Prevalence of substance use and psychiatric disorders in a highly select chronic pain population. *J Addict Med*. 2013;7(1):17-24. doi:https://dx.doi.org/10.1097/ADM.0b013e3182738655
274. Puto G, Repka I, Muszalik M. Factors Correlating with Functional Capacity in Older People with Chronic Pain. *Int J Environ Res Public Health*. 2023;20(3):03. doi:https://dx.doi.org/10.3390/ijerph20032748
275. Quidé Y, Norman-Nott N, Hesam-Shariati N, McAuley JH, Gustin SM. Is chronic pain an affective disorder? Moderation of resting-state functional connectivity within the emotional brain by depressive symptoms. 2022.
276. Quinlan J, Willson H, Grange K. Hopes and fears before opioid tapering: a quantitative and qualitative study of patients with chronic pain and long-term opioids. *Br*. 2021;15(2):120-128. doi:https://dx.doi.org/10.1177/2049463720974053
277. Racine M, Choiniere M, Nielson WR. Predictors of suicidal ideation in chronic pain patients: an exploratory study. *Clin J Pain*. 2014;30(5):371-8. doi:https://dx.doi.org/10.1097/AJP.0b013e31829e9d4d
278. Racine M, Sanchez-Rodriguez E, Galan S, et al. Factors Associated with Suicidal Ideation in Patients with Chronic Non-Cancer Pain. *Pain Med*. 2017;18(2):283-293. doi:https://dx.doi.org/10.1093/pm/pnw115
279. Radat F, Margot-Duclot A, Attal N. Psychiatric co-morbidities in patients with chronic peripheral neuropathic pain: a multicentre cohort study. *Eur J Pain*. 2013;17(10):1547-57. doi:https://dx.doi.org/10.1002/j.1532-2149.2013.00334.x
280. Rapariz-Gonzalez M, Castro-Diaz D, Mejia-Rendon D, Eurcis. Evaluation of the impact of the urinary symptoms on quality of life of patients with painful bladder syndrome/chronic pelvic pain and radiation cystitis: EURCIS study. *Actas Urol Esp*. 2014;38(4):224-31. doi:https://dx.doi.org/10.1016/j.acuro.2013.09.016
281. Rayner L, Hotopf M, Petkova H, Matcham F, Simpson A, McCracken LM. Depression in patients with chronic pain attending a specialised pain treatment centre: prevalence and impact on health care costs. *Pain*. 2016;157(7):1472-9. doi:https://dx.doi.org/10.1097/j.pain.0000000000000542
282. Reiter S, Eli I, Friedman-Rubin P, Emodi-Perlman A, Ziv-Baran T, Winocur E. Comparing Axis II Scores

© 2025 Aaron RV et al. *JAMA Network Open.*

According to the RDC/TMD and DC/TMD in Israeli Patients. *J Oral Facial Pain Headache*. 2017;31(4):323-330. doi:https://dx.doi.org/10.11607/ofph.1771
283. Reiter S, Eli I, Mahameed M, et al. Pain Catastrophizing and Pain Persistence in Temporomandibular Disorder Patients. *J Oral Facial Pain Headache*. 2018;32(3):309-320. doi:https://dx.doi.org/10.11607/ofph.1968
284. Rexelius N, Lindgren A, Torstensson T, Kristiansson P, Turkmen S. Sexuality and mood changes in women with persistent pelvic girdle pain after childbirth: a case-control study. *BMC Womens Health*. 2020;20(1):201. doi:https://dx.doi.org/10.1186/s12905-020-01058-7
285. Rezaei F, Neshat Doost HT, Molavi H, Abedi MR, Karimifar M. Depression and pain in patients with rheumatoid arthritis: Mediating role of illness perception. *Egyptian Rheumatologist*. 2014;36(2):57-64. doi:10.1016/j.ejr.2013.12.007
286. Rice D, Mehta S, Shapiro A, et al. Psychological Distress in Out-Patients Assessed for Chronic Pain Compared to Those with Rheumatoid Arthritis. *Pain Res Manag*. 2016;2016:7071907. doi:https://dx.doi.org/10.1155/2016/7071907
287. Robinson KM, Monsivais JJ. Depression, Depressive Somatic or Nonsomatic Symptoms, and Function in a Primarily Hispanic Chronic Pain Population. 2013;1:401732.
288. Rogal SS, Bielefeldt K, Wasan AD, Szigethy E, Lotrich F, DiMartini AF. Fibromyalgia symptoms and cirrhosis. *Dig Dis Sci*. 2015;60(5):1482-9. doi:https://dx.doi.org/10.1007/s10620-014-3453-3
289. Rogers J, Jones G, Cook JL, Wills K, Lahham A, Winzenberg TM. Chronic Plantar Heel Pain Is Principally Associated With Waist Girth (Systemic) and Pain (Central) Factors, Not Foot Factors: A Case-Control Study. *J Orthop Sports Phys Ther*. 2021;51(9):449-458. doi:https://dx.doi.org/10.2519/jospt.2021.10018
290. Rojas AM, Worts PR, Chandler Iii GS. Feasibility and Clinical Utility of Assessing Behavioral and Psychological Risk Factors in Pain Management. *Pain physician*. 2021;24(8):E1299-E1306.
291. Rometsch C, Ott S, Festl-Wietek T, et al. Mental disorders are no predictors to determine the duration of cannabis-based treatment for chronic pain. *Front Psychiatr*. 2022;13:1033020. doi:https://dx.doi.org/10.3389/fpsyt.2022.1033020
292. Rometsch C, Teufel M, Skoda EM, et al. Depression and anxiety mediate the relationship between illness representations and perceived distress in patients with chronic pain. *Sci*. 2023;13(1):15527. doi:https://dx.doi.org/10.1038/s41598-023-42156-4
293. Roseen EJ, Gerlovin H, Felson DT, Delitto A, Sherman KJ, Saper RB. Which Chronic Low Back Pain Patients Respond Favorably to Yoga, Physical Therapy, and a Self-care Book? Responder Analyses from a Randomized Controlled Trial. *Pain Med*. 2021;22(1):165-180. doi:https://dx.doi.org/10.1093/pm/pnaa153
294. Rouch I, Edjolo A, Laurent B, Pongan E, Dartigues JF, Amieva H. Association between chronic pain and long-term cognitive decline in a population-based cohort of elderly participants. *Pain*. 2021;162(2):552-560. doi:https://dx.doi.org/10.1097/j.pain.0000000000002047
295. Rouch I, Strippoli MF, Dorey JM, et al. Psychiatric disorders, personality traits, and childhood traumatic events predicting incidence and persistence of chronic pain: results from the CoLaus|PsyCoLaus study. *Pain*. 2023;164(9):2084-2092. doi:https://dx.doi.org/10.1097/j.pain.0000000000002912
296. Rovner GS, Sunnerhagen KS, Bjorkdahl A, et al. Chronic pain and sex-differences; women accept and move, while men feel blue. *PLoS ONE*. 2017;12(4):e0175737. doi:https://dx.doi.org/10.1371/journal.pone.0175737
297. Roy R, Sommer JL, Amadeo R, et al. Demographic and clinical characteristics of free-text writers in chronic pain patient intake questionnaires. *Can J Pain*. 2022;6(1):24-32. doi:https://dx.doi.org/10.1080/24740527.2021.2016031
298. Rus Makovec M, Vintar N, Makovec S. Self - Reported Depression, Anxiety and Evaluation of Own Pain in Clinical Sample of Patients with Different Location of Chronic Pain. *Zdrav*. 2015;54(1):1-10. doi:https://dx.doi.org/10.1515/sjph-2015-0001
299. Rusu AC, Santos R, Pincus T. Pain-related distress and clinical depression in chronic pain: A comparison between two measures. *Scand J Pain*. 2016;12:62-67. doi:https://dx.doi.org/10.1016/j.sjpain.2016.04.001
300. Saariaho AS, Saariaho TH, Mattila AK, Karukivi M, Joukamaa MI. Alexithymia and Early Maladaptive Schemas in chronic pain patients. *Scand J Psychol*. 2015;56(4):428-37. doi:https://dx.doi.org/10.1111/sjop.12226
301. Saariaho AS, Saariaho TH, Mattila AK, Karukivi MR, Joukamaa MI. Alexithymia and depression in a chronic pain patient sample. *Gen Hosp Psychiatry*. 2013;35(3):239-45. doi:https://dx.doi.org/10.1016/j.genhosppsych.2012.11.011
302. Saariaho AS, Saariaho TH, Mattila AK, Ohtonen P, Joukamaa MI, Karukivi M. Alexithymia and depression in the recovery of chronic pain patients: a follow-up study. *Nord J Psychiatry*. 2017;71(4):262-269. doi:https://dx.doi.org/10.1080/08039488.2016.1275782
303. Sachau J, Appel C, Reimer M, et al. Test-retest reliability of a simple bedside-quantitative sensory testing battery for chronic neuropathic pain. *Pain rep*. 2023;8(1):e1049. doi:https://dx.doi.org/10.1097/PR9.0000000000001049
304. Sagheer MA, Khan MF, Sharif S. Association between chronic low back pain, anxiety and depression in patients at a tertiary care centre. *JPMA J Pak Med Assoc*. 2013;63(6):688-90.
305. Saglam G, Akuzum F, Cetinkaya Alisar D. Assessment of psychiatric disorders and sleep quality in chronic lateral epicondylitis. *Agri Derg*. 2022;34(3):193-199. doi:https://dx.doi.org/10.14744/agri.2021.24119
306. Santos DM, Lage LV, Jabur EK, et al. The influence of depression on personality traits in patients with fibromyalgia: a case-control study. *Clin Exp Rheumatol*. 2017;35 Suppl 105(3):13-19.

© 2025 Aaron RV et al. *JAMA Network Open.*

307. Scherrer JF, Salas J, Lustman PJ, Burge S, Schneider FD. Change in opioid dose and change in depression in a longitudinal primary care patient cohort. *Pain*. 2015;156(2):348-355. doi:https://dx.doi.org/10.1097/01.j.pain.0000460316.58110.a0
308. Schmukler J, Li T, Gibson KA, Morla RM, Luta G, Pincus T. Patient global assessment is elevated by up to 5 of 10 units in patients with inflammatory arthritis who screen positive for fibromyalgia (by FAST4) and/or depression (by MDS2) on a single MDHAQ. *Semin Arthritis Rheum*. 2023;58:152151. doi:https://dx.doi.org/10.1016/j.semarthrit.2022.152151
309. Schroeter C, Ehrenthal JC, Giulini M, et al. Attachment, symptom severity, and depression in medically unexplained musculoskeletal pain and osteoarthritis: a cross-sectional study. *PLoS ONE*. 2015;10(3):e0119052. doi:https://dx.doi.org/10.1371/journal.pone.0119052
310. Schwab R, Stewen K, Ost L, et al. Predictors of Psychological Distress in Women with Endometriosis during the COVID-19 Pandemic. *Int J Environ Res Public Health*. 2022;19(8):18. doi:https://dx.doi.org/10.3390/ijerph19084927
311. Schwarm FP, Ott M, Nagl J, et al. Preoperative Elevated Levels for Depression, Anxiety, and Subjective Mental Stress Have No Influence on Outcome Measures of Peripheral Nerve Field Stimulation for Chronic Low Back Pain-A Prospective Study. *Neuromodulation*. 2021;24(6):1042-1050. doi:https://dx.doi.org/10.1111/ner.13368
312. Seed HF, Hazli Z, Perumal M, Azlin B. Depression among chronic pain patients at Hospital Tengku Ampuan Rahimah, Klang. *Med J Malaysia*. 2015;70(5):303-6.
313. Seekatz B, Meng K, Bengel J, Faller H. Is there a role of depressive symptoms in the fear-avoidance model? A structural equation approach. *Psychol Health Med*. 2016;21(6):663-74. doi:https://dx.doi.org/10.1080/13548506.2015.1111392
314. Sener U, Ucok K, Ulasli AM, et al. Evaluation of health-related physical fitness parameters and association analysis with depression, anxiety, and quality of life in patients with fibromyalgia. *International Journal of Rheumatic Diseases*. 2013;doi:10.1111/1756-185X.12237
315. Sengupta T, Paul B, Banerjee A, Das R, Halder R. Chronic musculoskeletal pain among elderly individuals in a rural area of West Bengal: A mixed-method study. *Malays Fam Physician*. 2023;18:25. doi:https://dx.doi.org/10.51866/oa.232
316. Shamji MF, Rodriguez J, Shcharinsky A, Paul D. High Rates of Undiagnosed Psychological Distress Exist in a Referral Population for Spinal Cord Stimulation in the Management of Chronic Pain. *Neuromodulation*. 2016;19(4):414-21. doi:https://dx.doi.org/10.1111/ner.12373
317. Sharma R, Queally M, Berkovitz S, Hughes JG. 'It's helped me to learn to deal with it': a mixed methods service evaluation of a multicomponent fibromyalgia service. *Clin Exp Rheumatol*. 2022;40(6):1189-1193. doi:https://dx.doi.org/10.55563/clinexprheumatol/m1ijpc
318. Shebeshi D, Allingham S, White J, Tardif H, Holloway D. Factors associated with general practitioner visits for pain in people experiencing chronic pain. *J Prim Health Care*. 2023;15(3):199-205. doi:https://dx.doi.org/10.1071/HC23004
319. Shmagel A, Foley R, Ibrahim H. Epidemiology of Chronic Low Back Pain in US Adults: Data From the 2009-2010 National Health and Nutrition Examination Survey. *Arthritis Care Res (Hoboken)*. 2016;68(11):1688-1694. doi:https://dx.doi.org/10.1002/acr.22890
320. Si H, Wang C, Jin Y, et al. Prevalence, Factors, and Health Impacts of Chronic Pain Among Community-Dwelling Older Adults in China. *Pain Manag Nurs*. 2019;20(4):365-372. doi:https://dx.doi.org/10.1016/j.pmn.2019.01.006
321. Silva K, Castro L, Costa-Santos C, Lourenco A, Lima M. More than Ownership: The Importance of Relationships with Companion Dogs for the Psychological Adjustment of Fibromyalgia Patients. *Pain Med*. 2021;22(12):2987-2997. doi:https://dx.doi.org/10.1093/pm/pnaa438
322. Siqueira-Campos VM, Fernandes LJH, de Deus JM, Conde DM. Parenting Styles, Mental Health, and Catastrophizing in Women with Chronic Pelvic Pain: A Case-Control Study. *Int J Environ Res Public Health*. 2022;19(20):16. doi:https://dx.doi.org/10.3390/ijerph192013347
323. Siqueira-Campos VME, Da Luz RA, de Deus JM, Martinez EZ, Conde DM. Anxiety and depression in women with and without chronic pelvic pain: prevalence and associated factors. *J Pain Res*. 2019;12:1223-1233. doi:https://dx.doi.org/10.2147/JPR.S195317
324. Sitges C, Gonzalez-Roldan AM, Duschek S, Montoya P. Emotional Influences on Cognitive Processing in Fibromyalgia Patients With Different Depression Levels: An Event-related Potential Study. *Clin J Pain*. 2018;34(12):1106-1113. doi:https://dx.doi.org/10.1097/AJP.0000000000000637
325. Slawek DE, Syed M, Cunningham CO, et al. Pain catastrophizing and mental health phenotypes in adults with refractory chronic pain: A latent class analysis. *J Psychiatr Res*. 2021;145:102-110. doi:https://dx.doi.org/10.1016/j.jpsychires.2021.12.001
326. Sleurs D, Tebeka S, Scognamiglio C, Dubertret C, Le Strat Y. Comorbidities of self-reported fibromyalgia in United States adults: A cross-sectional study from The National Epidemiological Survey on Alcohol and Related Conditions (NESARC-III). *Eur J Pain*. 2020;24(8):1471-1483. doi:https://dx.doi.org/10.1002/ejp.1585
327. Sohn HS, Lee DH, Lee KJ, et al. Impaired empathic abilities among patients with complex regional pain syndrome

© 2025 Aaron RV et al. *JAMA Network Open.*

(Type I). *Psychiatry Investigation*. 2016;13(1):34-42. doi:10.4306/pi.2016.13.1.34
328. Song S, Graham-Engeland JE, Mogle J, Martire LM. The effects of daily mood and couple interactions on the sleep quality of older adults with chronic pain. *J Behav Med*. 2015;38(6):944-55. doi:https://dx.doi.org/10.1007/s10865-015-9651-4
329. Soriano-Maldonado A, Amris K, Ortega FB, et al. Association of different levels of depressive symptoms with symptomatology, overall disease severity, and quality of life in women with fibromyalgia. *Qual Life Res*. 2015;24(12):2951-7. doi:https://dx.doi.org/10.1007/s11136-015-1045-0
330. Souza RC, de Sousa ET, Sousa D, et al. Prevalence of Temporomandibular Joint Disorders in Patients with Ankylosing Spondylitis: A Cross-Sectional Study. *Clin*. 2021;13:469-478. doi:https://dx.doi.org/10.2147/CCIDE.S320537
331. Sparkes E, Duarte RV, Mann S, Lawrence TR, Raphael JH. Analysis of psychological characteristics impacting spinal cord stimulation treatment outcomes: a prospective assessment. *Pain physician*. 2015;18(3):E369-77.
332. Stefani LC, Leite FM, da Graca LTM, et al. BDNF and serum S100B levels according the spectrum of structural pathology in chronic pain patients. *Neurosci Lett*. 2019;706:105-109. doi:https://dx.doi.org/10.1016/j.neulet.2019.05.021
333. Stehlik R, Ulfberg J, Zou D, Hedner J, Grote L. Morning cortisol and fasting glucose are elevated in women with chronic widespread pain independent of comorbid restless legs syndrome. *Scand J Pain*. 2018;18(2):187-194. doi:https://dx.doi.org/10.1515/sjpain-2018-0026
334. Stein MD, Herman DS, Bailey GL, et al. Chronic pain and depression among primary care patients treated with buprenorphine. *J Gen Intern Med*. 2015;30(7):935-41. doi:https://dx.doi.org/10.1007/s11606-015-3212-y
335. Steiner JL, Bigatti SM, Slaven JE, Ang DC. The Complex Relationship between Pain Intensity and Physical Functioning in Fibromyalgia: The Mediating Role of Depression. *J*. 2017;22(4)doi:https://dx.doi.org/10.1111/jabr.12079
336. Stokholm RN, Handberg C, Knudsen LF. Prevalence of chronic pain in a national cohort of patients with limb-girdle muscular dystrophy: a cross-sectional study. *Disabil Rehabil*. 2022;44(25):7802-7810. doi:https://dx.doi.org/10.1080/09638288.2021.1998669
337. Subramaniam M, Vaingankar JA, Abdin E, Chong SA. Psychiatric morbidity in pain conditions: results from the Singapore Mental Health Study. *Pain Res Manag*. 2013;18(4):185-90.
338. Sundara Rajan R, Bhatia A, Peng PWH, Gordon AS. Perineural steroid injections around ilioinguinal, iliohypogastric, and genitofemoral nerves for treatment of chronic refractory neuropathic pain: A retrospective study. *Can J Pain*. 2017;1(1):216-225. doi:https://dx.doi.org/10.1080/24740527.2017.1403846
339. Sundstrom FT, Lavefjord A, Buhrman M, McCracken LM. Assessing Psychological Flexibility and Inflexibility in Chronic Pain Using the Multidimensional Psychological Flexibility Inventory (MPFI). *J Pain*. 2023;24(5):770-781. doi:https://dx.doi.org/10.1016/j.jpain.2022.11.010
340. Tanaka T, Okita M, Jenkins S, Kozu R. Clinical and Psychological Impact of Chronic Pain in People with Chronic Obstructive Pulmonary Disease. *Int J Chron Obstruct Pulmon Dis*. 2022;17:893-903. doi:https://dx.doi.org/10.2147/COPD.S359223
341. Tardif H, Allingham SF, Rahman M, Daly A. Patterns of patient outcomes following specialist pain management in Australasia: a latent class analysis using the electronic Persistent Pain Outcomes Collaboration database. *Pain*. 2023;164(5):967-976. doi:https://dx.doi.org/10.1097/j.pain.0000000000002799
342. Tatebe M, Iwatsuki K, Hirata H, Oguchi T, Tanaka K, Urata S. Wrist and hand effects of depression and inflammatory factors on chronic conditions of the wrist. *Bone and Joint Journal*. 2016;98B(7):961-968. doi:10.1302/0301-620x.98b7.37152
343. Taylor S, Furness P, Ashe S, Haywood-Small S, Lawson K. Comorbid Conditions, Mental Health and Cognitive Functions in Adults with Fibromyalgia. *West J Nurs Res*. 2021;43(2):115-122. doi:https://dx.doi.org/10.1177/0193945920937429
344. Teixido-Abiol L, de Arriba-Arnau A, Segui Montesinos J, Herradon Gil-Gallardo G, Sanchez-Lopez MJ, De Sanctis Briggs V. Psychopathological and Personality Pro file in Chronic Nononcologic Nociceptive and Neuropathic pain: Cross-sectional Comparative Study. *n*. 2022;15(2):51-67. doi:https://dx.doi.org/10.21500/20112084.5631
345. Terassi M, Rossetti ES, Luchesi BM, Gramani-Say K, Hortense P, Pavarini SCI. Factors associated with depressive symptoms in elderly caregivers with chronic pain. *Rev Bras Enferm*. 2020;73(1):e20170782. doi:https://dx.doi.org/10.1590/0034-7167-2017-0782
346. Tetsunaga T, Misawa H, Tanaka M, Sugimoto Y, Takigawa T, Ozaki T. The clinical manifestations of lumbar disease are correlated with self-rating depression scale scores. *J Orthop Sci*. 2013;18(3):374-9. doi:https://dx.doi.org/10.1007/s00776-013-0363-8
347. Teychenne M, Lamb KE, Main L, et al. General strength and conditioning versus motor control with manual therapy for improving depressive symptoms in chronic low back pain: A randomised feasibility trial. *PLoS ONE*. 2019;14(8):e0220442. doi:https://dx.doi.org/10.1371/journal.pone.0220442
348. Thakral M, Walker RL, Saunders K, et al. Comparing Pain and Depressive Symptoms of Chronic Opioid Therapy Patients Receiving Dose Reduction and Risk Mitigation Initiatives With Usual Care. *Journal of Pain*. 2018;19(1):111-120. doi:10.1016/j.jpain.2017.09.006
349. Thi Nguy BH, Liu WT, Chang YT, Lin CP, Kang JH. Elevated tau and beta-amyloid in the serum of fibromyalgia

© 2025 Aaron RV et al. *JAMA Network Open.*

patients. *CNS Spectr*. 2022;27(3):339-346. doi:https://dx.doi.org/10.1017/S1092852920002114
350. Thomas EBK, Stegall MS, Farley KE, Pawlak SA. A Multidisciplinary Pelvic Pain Clinic: Integrated Health Psychology in a Specialty Care Setting. *J Womens Health (Larchmt)*. 2022;14:14. doi:https://dx.doi.org/10.1089/jwh.2022.0072
351. Thompson L, Van Dyne A, Sadler M, Cronan T. The Indirect Effects of Recalled Trauma Severity on Pain Ratings among People with Fibromyalgia: a Moderated Mediation Model. *Behav Med*. 2023:1-13. doi:https://dx.doi.org/10.1080/08964289.2023.2196389
352. Tocchetto BF, Ramalho L, Zortea M, et al. Peripheral body temperature rhythm as a marker of the severity of depression symptoms in fibromyalgia. *Biol Psychol*. 2023;177:108494. doi:https://dx.doi.org/10.1016/j.biopsycho.2023.108494
353. Tsuji T, Matsudaira K, Sato H, Vietri J. The impact of depression among chronic low back pain patients in Japan. *BMC Musculoskelet Disord*. 2016;17(1):447.
354. Uebelacker LA, Weisberg RB, Herman DS, Bailey GL, Pinkston-Camp MM, Stein MD. Chronic Pain in HIV-Infected Patients: Relationship to Depression, Substance Use, and Mental Health and Pain Treatment. *Pain Med*. 2015;16(10):1870-81. doi:https://dx.doi.org/10.1111/pme.12799
355. Upadhyaya SK, Malgutte DR, Handa R, Gupta S, Kumar A, Budumuru S. Fibromyalgia and mental health in rheumatoid arthritis: a cross-sectional prevalence study from the COVID-19 pandemic. *BMJ Open*. 2023;13(6):e069014. doi:https://dx.doi.org/10.1136/bmjopen-2022-069014
356. van den Berk-Clark C, Weaver TL, Schneider FD. Three Types of Intimate Relationships among Individuals with Chronic Pain and a History of Trauma Exposure. *Healthcare (Basel)*. 2017;5(4):29. doi:https://dx.doi.org/10.3390/healthcare5040068
357. van Eeden C, Mohazab N, Redmond D, et al. Myalgic encephalomyelitis/chronic fatigue syndrome (ME/CFS) and fibromyalgia: PR3-versus MPO-ANCA-associated vasculitis, an exploratory cross-sectional study. *Lancet Reg Health Am*. 2023;20:100460. doi:https://dx.doi.org/10.1016/j.lana.2023.100460
358. Van Overmeire R, Vesentini L, Vanclooster S, Muysewinkel E, Bilsen J. Sexual Desire, Depressive Symptoms and Medication Use Among Women With Fibromyalgia in Flanders. *Sex*. 2022;10(1):100457. doi:https://dx.doi.org/10.1016/j.esxm.2021.100457
359. Van Ryckeghem DML, De Houwer J, Van Bockstaele B, Van Damme S, De Schryver M, Crombez G. Implicit associations between pain and self-schema in patients with chronic pain. *Pain*. 2013;154(12):2700-2706. doi:https://dx.doi.org/10.1016/j.pain.2013.07.055
360. Vance CG, Chimenti RL, Dailey DL, et al. Development of a method to maximize the transcutaneous electrical nerve stimulation intensity in women with fibromyalgia. *J Pain Res*. 2018;11:2269-2278. doi:https://dx.doi.org/10.2147/JPR.S168297
361. Varinen A, Kosunen E, Mattila K, Suominen S, Sillanmaki L, Sumanen M. The association between bullying victimization in childhood and fibromyalgia. Data from the nationwide Finnish health and social support (HeSSup) study based on a sample of 64,797 individuals. *J Psychosom Res*. 2019;117:48-53. doi:https://dx.doi.org/10.1016/j.jpsychores.2018.12.003
362. Vilalta-Abella F, Gutierrez-Maldonado J, Pla-Sanjuanelo J. Development Of A Virtual Environment Based On The Perceived Characteristics Of Pain In Patients With Fibromyalgia. *Stud Health Technol Inform*. 2015;219:158-62.
363. Villafaina S, Sitges C, Collado-Mateo D, Fuentes-Garcia JP, Gusi N. Influence of depressive feelings in the brain processing of women with fibromyalgia: An EEG study. *Medicine (Baltimore)*. 2019;98(19):e15564. doi:https://dx.doi.org/10.1097/MD.0000000000015564
364. Voute M, Lambert C, Pereira B, Pickering G. Assessment of Initial Depressive State and Pain Relief With Ketamine in Patients With Chronic Refractory Pain. *JAMA netw*. 2023;6(5):e2314406. doi:https://dx.doi.org/10.1001/jamanetworkopen.2023.14406
365. Vukojević Z, Kovačević AD, Perić S, et al. Assessment of the neuropathic component in a chronic low back pain syndrome. *Vojnosanitetski Pregled*. 2022;79(1):25-30. doi:10.2298/VSP181002069V
366. Wadley AL, Iacovides S, Roche J, et al. Working nights and lower leisure-time physical activity associate with chronic pain in Southern African long-distance truck drivers: A cross-sectional study. *PLoS ONE*. 2020;15(12):e0243366. doi:https://dx.doi.org/10.1371/journal.pone.0243366
367. Wadley AL, Pincus T, Evangeli M. A preliminary analysis of the association between perceived stigma and HIV-related pain in South Africans living with HIV. *Afr*. 2019;11(1):e1-e5. doi:https://dx.doi.org/10.4102/phcfm.v11i1.1647
368. Wahlman M, Hakkinen A, Dekker J, Marttinen I, Vihtonen K, Neva MH. The prevalence of depressive symptoms before and after surgery and its association with disability in patients undergoing lumbar spinal fusion. *Eur Spine J*. 2014;23(1):129-34. doi:https://dx.doi.org/10.1007/s00586-013-2896-0
369. Weingarten TN, Vincent A, Luedtke CA, et al. The Perception of Female Smokers with Fibromyalgia on the Effects of Smoking on Fibromyalgia Symptoms. *Pain pract*. 2016;16(8):1054-1063. doi:https://dx.doi.org/10.1111/papr.12402
370. Whitlock EL, Diaz-Ramirez LG, Glymour MM, Boscardin WJ, Covinsky KE, Smith AK. Association Between

© 2025 Aaron RV et al. *JAMA Network Open.*

Persistent Pain and Memory Decline and Dementia in a Longitudinal Cohort of Elders. *JAMA Intern Med*. 2017;177(8):1146-1153. doi:https://dx.doi.org/10.1001/jamainternmed.2017.1622
371. Widenka M, Leppert W. Assessment of the quality of life in elderly patients with osteoarthritis during the treatment of chronic pain with transdermal buprenorphine. *Acta Poloniae Pharmaceutica - Drug Research*. 2021;78(4):573-582. doi:10.32383/appdr/141347
372. Wildes M, Bigand TL, Layton ME, Wilson M. Cannabis Use and Cognition in Adults Prescribed Opioids for Persistent Pain. *Pain Manag Nurs*. 2020;21(1):94-99. doi:https://dx.doi.org/10.1016/j.pmn.2019.06.014
373. Wilson JM, Colebaugh CA, Flowers KM, et al. Applying the Rapid OPPERA Algorithm to Predict Persistent Pain Outcomes Among a Cohort of Women Undergoing Breast Cancer Surgery. *J Pain*. 2022;10:10. doi:https://dx.doi.org/10.1016/j.jpain.2022.07.012
374. Wilson JM, Colebaugh CA, Meints SM, Flowers KM, Edwards RR, Schreiber KL. Loneliness and Pain Catastrophizing Among Individuals with Chronic Pain: The Mediating Role of Depression. *J Pain Res*. 2022;15:2939-2948. doi:https://dx.doi.org/10.2147/JPR.S377789
375. Wilson M, Roll JM, Corbett C, Barbosa-Leiker C. Empowering Patients with Persistent Pain Using an Internet-based Self-Management Program. *Pain Manag Nurs*. 2015;16(4):503-14. doi:https://dx.doi.org/10.1016/j.pmn.2014.09.009
376. Wolfe F, Brahler E, Hinz A, Hauser W. Fibromyalgia prevalence, somatic symptom reporting, and the dimensionality of polysymptomatic distress: results from a survey of the general population. *Arthritis Care Res (Hoboken)*. 2013;65(5):777-85. doi:https://dx.doi.org/10.1002/acr.21931
377. Wong HJ, Anitescu M. The Role of Health Locus of Control in Evaluating Depression and Other Comorbidities in Patients with Chronic Pain Conditions, A Cross-Sectional Study. *Pain pract*. 2017;17(1):52-61. doi:https://dx.doi.org/10.1111/papr.12410
378. Wright E, Zarnegar R, Hermansen I, McGavin D. A clinical evaluation of a community-based rehabilitation and social intervention programme for patients with chronic pain with associated multi-morbidity. *Journal of Pain Management*. 2017;10(2):149-159.
379. Xu Y, Wang Y, Chen J, et al. The comorbidity of mental and physical disorders with self-reported chronic back or neck pain: Results from the China Mental Health Survey. *J Affect Disord*. 2020;260:334-341. doi:https://dx.doi.org/10.1016/j.jad.2019.08.089
380. Yadav D, Askew RL, Palermo T, et al. Association of Chronic Pancreatitis Pain Features With Physical, Mental, and Social Health. *Clin Gastroenterol Hepatol*. 2023;21(7):1781-1791.e4. doi:https://dx.doi.org/10.1016/j.cgh.2022.09.026
381. Yamada AS, Simon D, Antunes FTT, Say KG, Souza AHD. Psychosocial factors associated with disability in patients with non-specific chronic low back pain: A cross-sectional study. *Rehabilitacion*. 2022;19:19. doi:https://dx.doi.org/10.1016/j.rh.2022.06.002
382. Yin MXC, Chan JSM, Lau BHP, et al. A self-administered moxibustion-cum-massage intervention for older adults with chronic pain in the community: A randomized controlled trial. *Complement Ther Med*. 2023;72:102908. doi:https://dx.doi.org/10.1016/j.ctim.2022.102908
383. Yu L, Kioskli K, McCracken LM. The Psychological Functioning in the COVID-19 Pandemic and Its Association With Psychological Flexibility and Broader Functioning in People With Chronic Pain. *J Pain*. 2021;22(8):926-939. doi:https://dx.doi.org/10.1016/j.jpain.2021.02.011
384. Zaidel C, Musich S, Karl J, Kraemer S, Yeh CS. Psychosocial Factors Associated with Sleep Quality and Duration Among Older Adults with Chronic Pain. *Popul Health Manag*. 2021;24(1):101-109. doi:https://dx.doi.org/10.1089/pop.2019.0165
385. Zakrzewska JM, Wu J, Mon-Williams M, Phillips N, Pavitt SH. Evaluating the impact of trigeminal neuralgia. *Pain*. 2017;158(6):1166-1174. doi:10.1097/j.pain.0000000000000853
386. Zambelli Z, Halstead EJ, Fidalgo AR, Dimitriou D. Good Sleep Quality Improves the Relationship Between Pain and Depression Among Individuals With Chronic Pain. *Front Psychol*. 2021;12:668930. doi:https://dx.doi.org/10.3389/fpsyg.2021.668930
387. Zubatsky M, Witthaus M, Scherrer JF, et al. The association between depression and type of treatments received for chronic low back pain. *Fam Pract*. 2020;37(3):348-354. doi:https://dx.doi.org/10.1093/fampra/cmz062

© 2025 Aaron RV et al. *JAMA Network Open.*
