# Supplementary material for: Prevalence of Depression and Anxiety Among Adults With Chronic Pain: A Systematic Review and Meta-Analysis
Source: JAMA Netw Open. 2025 Mar 7;8(3):e250268. doi: 10.1001/jamanetworkopen.2025.0268 (PMC11889470; doi:10.1001/jamanetworkopen.2025.0268)
Supplement: Supplement 2. — Data Sharing Statement [file jamanetwopen-e250268-s002.pdf]

# Data Sharing Statement

Aaron. Prevalence of Depression and Anxiety Among Adults With Chronic Pain. *JAMA Netw Open*. Published March 07, 2025. doi:10.1001/jamanetworkopen.2025.0268

**Data**
**Data available:** Yes
**Data types:** Other (please specify)
**Additional Information:** All data (extracted from published records) and study forms will be available.
**How to access data:** Data is available at request to first author (Raaron4@jhmi.edu)
**When available:** With publication

**Supporting Documents**
**Document types:** Statistical/analytic code, Other (please specify)
**Additional Information:** Study forms
**How to access documents:** at first author request
**When available:** With publication

**Additional Information**
**Who can access the data:** anyone requesting the data
**Types of analyses:** any analysis
**Mechanisms of data availability:** with investigator support
**Any additional restrictions:** none
